# Fibril formation and ordering of disordered FUS LC driven by hydrophobic interactions

**DOI:** 10.1038/s41557-023-01221-1

**Published:** 2023-05-25

**Authors:** Daria Maltseva, Sayantan Chatterjee, Chun-Chieh Yu, Mateusz Brzezinski, Yuki Nagata, Grazia Gonella, Anastasia C. Murthy, Jeanne C. Stachowiak, Nicolas L. Fawzi, Sapun H. Parekh, Mischa Bonn

**Affiliations:** 1 Max Planck Institute for Polymer Research, Mainz, Germany; 2 Department of Biomedical Engineering, University of Texas at Austin, Austin, TX USA; 3 Institute of Biochemistry and Bringing Materials to Life Initiative, ETH Zurich, Zürich, Switzerland; 4 Department of Molecular Biology, Cell Biology, and Biochemistry, Brown University, Providence, RI USA

**Keywords:** Chemical physics, Intrinsically disordered proteins

## Abstract

Biomolecular condensates, protein-rich and dynamic membrane-less organelles, play critical roles in a range of subcellular processes, including membrane trafficking and transcriptional regulation. However, aberrant phase transitions of intrinsically disordered proteins in biomolecular condensates can lead to the formation of irreversible fibrils and aggregates that are linked to neurodegenerative diseases. Despite the implications, the interactions underlying such transitions remain obscure. Here we investigate the role of hydrophobic interactions by studying the low-complexity domain of the disordered ‘fused in sarcoma’ (FUS) protein at the air/water interface. Using surface-specific microscopic and spectroscopic techniques, we find that a hydrophobic interface drives fibril formation and molecular ordering of FUS, resulting in solid-like film formation. This phase transition occurs at 600-fold lower FUS concentration than required for the canonical FUS low-complexity liquid droplet formation in bulk. These observations highlight the importance of hydrophobic effects for protein phase separation and suggest that interfacial properties drive distinct protein phase-separated structures.

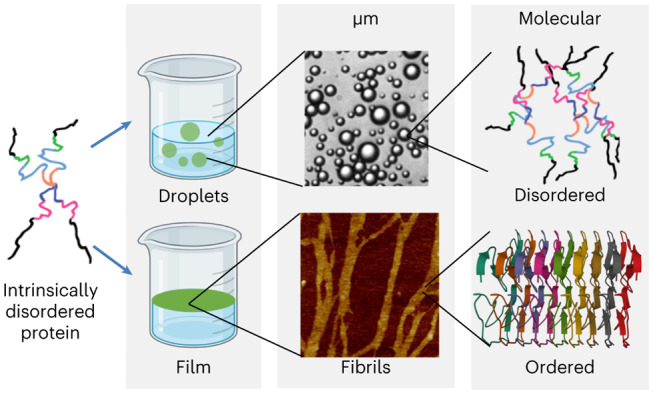

## Abstract (web summary)

Protein solutions can undergo liquid–liquid phase separation, by condensing into a dense phase that often resembles liquid droplets, which coexist with a dilute phase. Now it is shown that hydrophobic interactions, specifically at interfaces, can trigger a liquid–solid phase separation of a protein solution.

## Main

Intracellular biomolecular species (RNA, DNA, proteins) are now well known to condense into membrane-less organelles, driven in part by liquid–liquid phase separation (LLPS)^1–4^. These membrane-less organelles are also known as biomolecular condensates (BCs). Numerous studies have shown that more than 30% of the eukaryotic proteome is intrinsically disordered by nature^5–9^, and such intrinsically disordered proteins (IDPs) appear to have a propensity to localize to, or contribute to the formation of, BCs such as nucleoli, paraspeckles, Cajal bodies, P-bodies, germ granules and stress granules^10^. Moreover, these BCs play a crucial role in many subcellular processes, including membrane trafficking, transcriptional regulation, enzyme and substrate concentration for enhanced reaction kinetics, sequestration for reaction inhibition and so on^4,11,12^. However, IDPs can transform their condensates into the irreversible pathological aggregates that are responsible for neurodegenerative diseases^13,14^ such as Parkinson’s (from aggregation of α-synuclein)^15^, Alzheimer’s (from aggregation of amyloid-β and tau-protein)^16^, Huntington’s diseases (from huntingtin aggregation)^17^, and amyotrophic lateral sclerosis and frontotemporal dementia (from fused in sarcoma (FUS) aggregation)^18–20^. Despite findings demonstrating the conversion from being liquid-like to pathological solid-like aggregations, the drivers for this conversion remain unclear. Although the role of lipids in BCs and (more so) aggregate formation has been investigated in detail, hydrophobic interactions, the motivation of the current study, have been less studied^21–26^.

Among the many BC-forming IDPs we focus on FUS, as it has been independently studied in the form of aggregates, liquid droplets and hydrogels. FUS is an RNA-binding protein^27^ that forms stress granules in cells and can form BCs in vitro via LLPS^18–20,28,29^. FUS participates in various cellular processes, including transcription, micro RNA (miRNA) processing, messenger RNA (mRNA) transport and translation, and DNA repair initiation^30,31^. FUS consists of three main domains: the C-terminal RGG and zinc finger domains, the RNA-recognition motif and the N-terminal low-complexity domain enriched in polar residues (QGSY-rich), making it prion-like (that is, resembling the residue composition of yeast prion domains)^27,32^. In this Article we study the N-terminal QGSY-rich intrinsically disordered FUS low-complexity domain (FUS LC, 1–163 amino acids), which phase-separates in vitro into liquid-like BCs under physiological conditions^29,33^. Beyond its vital functionality, FUS LC has been shown to drive the aggregation of FUS into toxic protein inclusions^34^. In physiologically relevant solution conditions, purified FUS LC forms condensates via LLPS at a concentration of ~100 μM in the absence of crowding agents^33^. BCs derived from FUS LC are formed due to weak multivalent interactions, such as *π*–*π*, hydrophobic and cation–*π* interactions^33,35^. Multiple studies have been performed to investigate the bulk properties of FUS LC BCs using a range of microscopic and spectroscopic techniques^29,33,36^. However, our understanding of the role of interfaces, and in particular hydrophobic interfaces, on FUS phase behaviour is quite limited. Hydrophobic interfaces are common inside cells. There are static hydrophobic–hydrophilic patches on large protein assemblies in cells, and dynamic transient exposure of hydrophobic–hydrophilic regions in general^37–39^. Moreover, monolayer-coated lipid bodies are ubiquitous in cells and provide another source of hydrophobic substrate, with which a considerable number of proteins interact. Finally, this class of interfaces is clearly important in FUS aggregation, as rapid shaking, rocking or vortexing, that is, entraining air, induces FUS LC aggregation^40,41^.

In this Article we report the behaviour of FUS LC at the air/water interface, a common model for a hydrophobic surface^42^, under physiological buffer-solution conditions. We investigate the surface activity, organization and morphology of FUS LC, as well as the organization of interfacial water at the protein surface^43,44^. The air/water interface is used as a model system^45^, with buffer as a hydrophilic medium and air as a hydrophobic medium to study whether/how hydrophobic media can affect FUS LC molecular self-assembly. We combine several surface-sensitive techniques to elucidate the interfacial organization of FUS. Together, these techniques provide a multiscale picture of interfacial protein assembly and ordering of FUS LC driven by hydrophobic interactions from the molecular to macroscopic length scales.

## Results and discussion

### Structure and dynamics of FUS LC at the solution interface

**Fig. 1:**
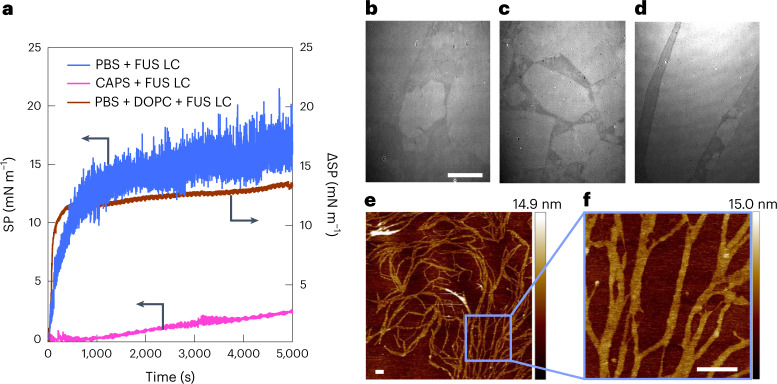
Mechanical and structural properties of FUS LC at an air/buffer solution interface. **a**, SP change versus time for samples of FUS LC in PBS and CAPS buffers (blue and magenta, resp., left axis), and a DOPC phospholipid monolayer spread at 20 mN m^−1^ at the air/PBS interface (brown, right axis), after the addition of FUS LC to a final bulk concentration of 1.5 μM (in PBS and CAPS) or 3 μM (in PBS with DOPC monolayer) at *t* = 0. The strongly fluctuating SP for PBS (absent for CAPS and DOPC) is attributed to solid domains colliding with the needle recording the surface tension. **b**–**d**, BAM images recorded 2 h (**b**), 4 h (**c**) and 5 h (**d**) after addition of 1.5 μM FUS LC in PBS (at *t* = 0), showing the appearance of plate-like domains on the surface. Scale bar, 100 μm (**b**–**d**). **e**,**f**, AFM image of the FUS LC protein film formed at the air/PBS buffer interface and deposited on a silicon wafer (**e**) and zoomed-in image (**f**). Scale bars, 250 nm (**e**,**f**). Vertical colour bars represent height in nm. Source data

The typical critical phase-separation concentration of the FUS LC segment at physiological pH in phosphate-buffered saline (PBS) solution is ~100 μM^33^. At higher pH (20 mM *N*-cyclohexyl-3-aminopropanesulfonic acid (CAPS) buffer at pH 11), FUS LC remains in its monomeric form up to millimolar concentrations due to the deprotonation of the 24 tyrosine residues resulting in a highly negatively charged peptide^29^. To understand the organization/phase transition of IDPs at the air/buffer solution interface at various pH values, we used 1.5 μM FUS LC to avoid bulk phase transitions. To probe the adsorption propensity of FUS LC towards the air/buffer (CAPS versus PBS) solution interface at different pH values, we monitored the surface pressure (SP) versus time. Figure 1a shows that the protein adsorbs more rapidly and to a greater extent at pH 7.4 than at pH 11, where FUS LC molecules are stabilized in a monomeric state^29,46^. With 20 mM CAPS at pH 11, a SP of less than 1.5 mN m^−1^ is observed, and when diluted appropriately into a PBS subphase, less than 0.2 mN m^−1^, which is negligible compared with FUS LC in PBS (Supplementary section III). Additionally, the SP change from FUS LC adsorbing to the air/buffer solution interface was similar to that for FUS LC adsorbing to a 1,2-dioleoyl-*sn*-glycero-3-phosphocholine (DOPC) lipid monolayer under similar conditions (Fig. 1a, brown line, and Supplementary Fig. 1). Although the overall SP increase was similar, we observed substantially larger SP fluctuations at the air/buffer solution interface compared to the lipid interface. Because the SP fluctuations persisted even after SP equilibration, we hypothesize that FUS LC at the air/PBS buffer interface forms a porous, solid-like film. Such a film could produce large SP fluctuations in our set-up because the tensiometer needle position is fixed, whereas the trough containing the solution is constantly rotating, and solid film particulates will transiently collide with the needle, causing large fluctuations.

To test this hypothesis and characterize the structural properties of interfacial FUS LC organization at different spatial scales, we investigated FUS LC film formation independently at different pH values using Brewster angle microscopy (BAM) and atomic force microscopy (AFM). BAM images were recorded at 1-h intervals after addition of FUS LC to PBS solution. The protein forms domains at the air/PBS interface, as shown in Fig. 1b–d. The rectangular, sharp-edged shape of the domains indicates that line tension does not control the domain boundaries; for liquid domains, one typically expects circular-like domains due to reduction of the line tension for circular shapes (at sufficiently long timescales). Domain formation at the interface becomes more pronounced with time, and eventually we observe extended homogeneous interfacial regions, spanning over hundreds of micrometres, 6 h after protein addition (Supplementary Fig. 7). The formed film was sufficiently stable that it was possible to transfer it onto a substrate for imaging at the nanometre scale by AFM (Fig. 1e,f). With AFM we observe a two-dimensional fibrillar network of FUS LC that apparently gives rise to a porous film at mesoscopic length scales, supporting our hypothesis about the origin of the SP fluctuations observed in Fig. 1a. The FUS LC fibrillar assemblies are, on average, several nanometres tall (in the direction perpendicular to the interface) and up to 100 nm wide (Supplementary Fig. 10). Remarkably, FUS LC film formation is also observed at pH 7.4, at a tenfold lower FUS LC concentration (0.15 μM; Supplementary Fig. 8). In contrast, BAM images for FUS LC at pH 11 (in 20 mM CAPS) show no evidence of film formation, even 6 h after protein addition (Supplementary Fig. 9). This observation is consistent with the SP result for FUS in pH 11 CAPS buffer (Fig. 1a, magenta curve), which looks similar to SP measurements of a classic bulk fluid and is consistent with previous work showing that FUS LC does not self-assemble at pH 11^29^.

**Fig. 2:**
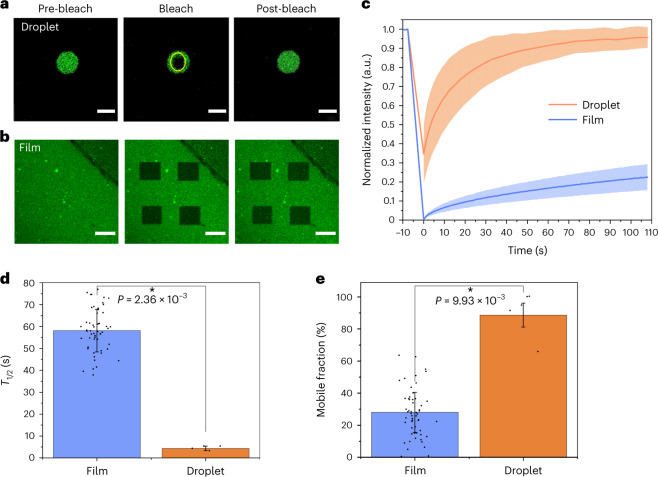
FRAP experiments reveal the liquid-like structure of FUS LC droplets formed in bulk and the solid-like structure of the FUS LC film formed at the air/PBS buffer interface. **a**,**b**, Pre-bleach, bleach and post-bleach fluorescence images for the FUS LC droplet (**a**) and FUS LC film (**b**). Scale bars, 5 μm (**a**), 20 μm (**b**). **c**, Fluorescence intensity versus time from *N* = 5 curves for droplets and *N* = 56 curves for films. The dark lines show the means, and the shaded regions are standard deviations. **d**,**e**, Half-time *T*_1/2_ of the fluorescence recovery (obtained from an exponential fit to the traces) (**d**) and mobile fraction values obtained for the FUS LC droplets and film (**e**). Bars represent the means, and error bars are standard deviations. Asterisks indicate statistical significance: *P* < 0.05 via Student’s *t*-test. Source data

To further investigate the mechanical properties of the FUS LC film in situ, we performed fluorescence recovery after photobleaching (FRAP) experiments^47^ on the protein film formed after adding 5 μM FUS LC (5% fluorescently labelled) into the PBS subphase. We compared the data with those obtained for FUS LC liquid droplets in PBS. Pre-bleach, bleach and post-bleach fluorescence images of the FUS LC droplet and FUS LC film are presented in Fig. 2a and 2b, respectively. The FRAP data show slow and limited fluorescence recovery in the film compared to the droplets (Fig. 2c). Fluorescence recovery is substantially more rapid and more complete (in terms of %) for FUS LC droplets, as expected for liquid-like condensates (Fig. 2c, blue curve)^48^. The values for the fluorescence half-time (*T*_1/2_) and the mobile fraction of FUS LC in FUS LC droplets versus film are presented in Fig. 2d and 2e, respectively. *T*_1/2_ reports on the protein mobility. The mobile fraction is ~100% if the labelled molecules are completely mobile and less with an increasingly immobile population. To compare the mobilities of the protein molecules in the droplets and film quantitatively, we calculated the diffusion coefficients *D* from the *T*_1/2_ values and bleach areas^49^; *D*_droplet_ = 0.3 ± 0.17 µm^2^ s^−1^ and *D*_film_ = 0.13 ± 0.02 µm^2^ s^−1^, which are statistically different (*P* < 0.05) from one another. The smaller mobile-fraction values for the FUS LC film organized at the air/PBS interface than for the FUS LC droplets in bulk corroborate the more solid-like protein organization in the film. The FRAP data thus indicate the formation of a new, solid-like phase that is dramatically different from the LLPS-derived phase of FUS BCs in bulk, in agreement with our SP, BAM and AFM results.

### FUS LC film and associated water molecular structure

**Fig. 3:**
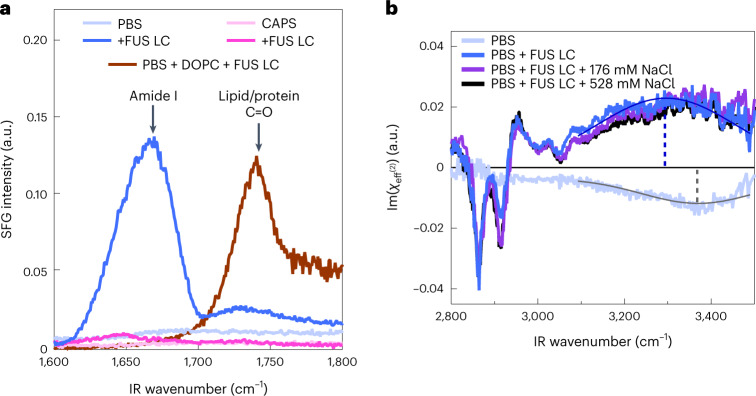
Molecular-level structure of the FUS LC film and orientation of protein-bound water molecules. **a**, SFG intensity (|*χ*^(2)^|^2^ spectra in the amide I and carbonyl stretching frequency region for 1.5 μM FUS LC in PBS (blue) and CAPS (magenta) buffers, and for 3 μM FUS LC at the DOPC monolayer spread at 20 mN m^−1^ at the air/PBS interface (brown). **b**, Im(*χ*^(2)^) spectra in the CH and OH stretching frequency region for PBS in the absence of FUS LC (light blue), PBS + 1.5 μM FUS LC (blue), PBS + 1.5 μM FUS LC + 176 mM NaCl (purple) and PBS + 1.5 μM FUS LC + 528 mM NaCl (black). Thin lines represent Gaussian fits for Im(*χ*^(2)^) spectra of the OH stretching response for PBS (grey) and PBS + FUS LC (dark blue). Vertical dashed lines are the central frequency values. Source data

Given that FUS LC forms a porous, solid-like film at the air/buffer solution interface at such low concentrations compared to liquid droplet formation, some questions logically follow. What is the nature of the protein in the fibrils? Are proteins structured in the film, or is the protein still disordered? What is the driving force for fibrillization? To answer these questions we studied the molecular-level properties of the interfacial FUS LC film by means of vibrational sum-frequency generation (SFG) spectroscopy. SFG spectroscopy provides the vibrational spectrum of molecules specifically at an interface. In addition, SFG is highly sensitive to the ordering of interfacial molecules. SFG spectra acquired after equilibration of protein organization at the air/PBS buffer solution interfaces are displayed in Fig. 3a. Throughout this Article we present steady-state SFG spectra from systems that appeared fully equilibrated. Time-dependent SFG spectra are provided in Supplementary section IX. The SFG data reveal a prominent amide I signal centred at ~1,670 cm^−1^, arising from vibrations in the protein backbone, and a C=O signal centred at ~1,730 cm^−1^, from the protein side chains^50^. In comparison, the amide I feature was almost invisible with FUS LC in pH 11 CAPS, further highlighting the difference in FUS LC molecular ordering at the air/PBS versus air/CAPS solution interface. The side-chain C=O signal, also only apparent at pH 7.4, suggests an ordered organization of C=O moieties present in the side chains of glutamine amino-acid residues. The broad spectral feature in the amide I region (full-width at half-maximum of ~50 cm^−1^) with its centre at ~1,670 cm^−1^ indicates that the protein fibrillary structures observed with AFM contain mixed secondary structure contributions of random coils, α-helices^51^ and β-type motifs^50,52–60^. Thus, our SFG data show that FUS LC, which is intrinsically disordered in solution and in liquid-like BCs^29^, appears to order at the air/PBS solution interface. In contrast, only a very small SFG amide I signal was detected for FUS LC, and no film formation was observed at pH 11. Interestingly, FUS LC also did not order/structure at the DOPC monolayer (Fig. 3a), despite the similar surface propensity towards the air/PBS buffer as indicated by SP measurements (Fig. 1a). These results highlight the unique ability of the hydrophobic interface to drive ordered self-assembly of the FUS LC.

As an additional control, we tested the interfacial behaviour of the FUS 12E LC mutant, which is known to stay monomeric even under physiological conditions^33,61^. We found that the SP change relative to the air/PBS solution interface observed for 1.5 μM FUS 12E LC is similar to that obtained for 0.15 μM FUS LC (Supplementary Fig. 12a). This result indicates that the interfacial adsorption propensity for FUS 12E LC is considerably less than that of wild-type FUS LC. We further find that the amide I SFG signal intensity is higher for 0.15 μM FUS LC than for 1.5 μM FUS 12E LC (Supplementary Fig. 12b), similarly indicating more substantial ordering for the wild type, despite the tenfold lower concentration. Because LLPS of FUS LC in the bulk at pH 7.4 strongly depends on protein concentration and can be kinetically controlled^41^, we also tested the effect of protein bulk concentration (in the range 0.5–6 μM) and the injection concentration of FUS LC on the adsorption and organization of FUS LC at the air/PBS solution interface. We observed ordered FUS LC organization at the air/PBS solution interface for all studied concentrations (Supplementary Figs. 12b and 13) and relatively minimal dependence on the injection concentration of FUS LC (Supplementary Fig. 14).

Interestingly, amide I SFG spectra acquired for FUS LC over the entire concentration range, including those for equilibrated films, as well as the time-dependent spectra taken during FUS LC film equilibration (Supplementary Fig. 11), do not present any dominant spectral feature at ~1,620–1,630 cm^−1^, characteristic for amyloid fibrils, despite FUS LC being organized into fibrils in the film, as evidenced by AFM. Because SFG is a nonlinear spectroscopic technique that is sensitive to both the structure and orientation of interfacial molecules, the amplitude of the signal also depends on the molecular orientation. To exclude that a specific orientation of FUS LC fibrils is the reason for the negligible SFG signal intensity at ~1,620 cm^−1^, we performed Fourier-transform infrared (FTIR) spectroscopy experiments on the FUS LC films. The FTIR measurements were performed in two distinct ways: on-liquid FUS LC films were measured in situ with the polarization modulation infrared reflection absorption (PM-IRRAS) technique, and for the measurement of the FUS LC film deposited on a solid substrate, conventional FTIR in transmission mode was employed. These measurements are presented in Supplementary section XIII. The vibrational response in the amide I region detected with both FTIR methods is in good agreement with the SFG spectra: no dominant contribution at ~1,620 cm^−1^ was observed, in contrast to FTIR (and SFG) spectra reported for conventional amyloid fibrils^59,62,63^. These experiments are consistent with the scenario that FUS LC fibril formation at the air/water interface is controlled by hydrophobic side-chain interactions, rather than by amide hydrogen bonding, as established for the cross-β spine structure of conventional amyloid-like fibrils, which gives rise to the 1,620-cm^−1^ mode^64–67^. This untypical behaviour is not intrinsic to FUS and may arise from the hydrophobic driving force that we probe. Indeed, a recent study has revealed that a slightly longer FUS segment (2–214) can form typical amyloid-like fibrils^68^.

As known from work on the hIAPP protein, the correlated appearance of both amide I and amide II SFG signals for the hIAPP protein serves as a reporter of amyloid oligomer/fibril assembly^59^. As such, we investigated the amide II SFG spectra of the FUS LC film (Supplementary Fig. 17). Both steady-state and kinetic spectra show a low-intensity amide II vibrational response, too low for reliable analysis and, more importantly, inconsequential in comparison with the intense amide I signal.

Taking into account the ordered accumulation of FUS LC at the amphiphilic air/PBS interface, a further question naturally arises regarding how FUS LC interacts with (inter)cellular membranes. Recently it has been reported that the interaction of FUS LC with negatively charged phosphatidylserine (PS) and phosphatidylglycerol (PG) membranes is largely defined by the lipid headgroup chemistry^69^. In fact, our data on FUS LC interactions with zwitterionic (net neutrally charged at pH 7.4) phosphatidylcholine (PC) and positively charged trimethylammonium-propane (TAP) lipid/water interfaces further confirm the point that the FUS LC/membrane interaction is controlled by the lipid headgroup (Supplementary Fig. 18).

Given the observed interfacial ordering and structuring of FUS LC at the amphiphilic air/PBS buffer interface, we hypothesized that interfacial protein ordering exposes hydrophobic domains of FUS LC to air, and hydrophilic domains towards the aqueous phase. The latter could lead to an aligned water network at the protein–water interface via hydrogen bonding between the protein and water. The side chains of tyrosine, glutamine and serine amino acids in FUS LC all have hydrogen-bonding capabilities that could affect interfacial water ordering. To probe the interfacial water organization at the protein film, the OH stretching mode^70^ was probed (Supplementary Fig. 19). To determine the interfacial water orientation, we used heterodyne-detected SFG (HD-SFG) spectroscopy, which provides a direct measurement of the imaginary component of the second-order susceptibility, Im(*χ*^(2)^)^71,72^. The sign of the Im(*χ*^(2)^) feature reports on the net interfacial molecular orientation.

Figure 3b shows the Im(*χ*^(2)^) spectra in the C–H stretch (2,800–3,050 cm^−1^) and O–H stretch (3,000–3,600 cm^−1^) frequency region. The sign of the Im(*χ*^(2)^) signal for water’s OH stretching mode at the air/PBS solution interface (Fig. 3b, light blue curve) is negative, indicating that the OH groups of the interfacial water molecules are, on average, oriented down towards the bulk solution. Upon adding FUS LC in the PBS subphase, the sign of the OH stretching signal flips (blue curve), meaning the OH groups on average point up towards the interface and the protein film. The reversal of the water orientation and a redshift of the response (as is apparent from the central frequency obtained from Gaussian fits to the water bands, indicated by vertical dashed lines) indicate that interfacial water molecules strongly interact with the protein^73^. Such flipping of the OH group can be attributed to either the charge of the protein or the protein–water hydrogen-bonding interaction; however, FUS LC is nearly uncharged at pH 7.4^29^.

As a control experiment, we show HD-SFG spectra for FUS LC in 20 mM CAPS pH 11 (Supplementary Fig. 20). The data show that the Im(*χ*^(2)^) signal is positive. However, FUS LC is injected into the PBS subphase, where the CAPS concentration is considerably lower (0.8 mM) and the pH is essentially 7.4. For 0.8 mM CAPS in PBS, the HD-SFG spectra show a signal that is indistinguishable from pure PBS (Supplementary Fig. 21), proving that the flipping of the water signal detected for FUS LC is not caused by CAPS molecules in PBS but rather by the FUS LC film.

To verify that charge does not play an important role in aligning interfacial water, NaCl was added into the PBS subphase to reduce the Debye length and screen any surface charge present in the film^74^. On adding ~180 mM (Fig. 3b, purple line) and ~530 mM (black line) NaCl, no major change in the HD-SFG water response was detected (for a detailed analysis, see Supplementary section XVIII). This observation demonstrates that the water signal originates mainly from water directly interacting with the protein at the interface. The presence of ordered FUS LC at the interface is sufficient to invert the orientation of OH moieties compared to that in the absence of FUS LC.

We emphasize that the vibrational response of water in contact with a protein at the water/air interface is nontrivial. For comparison, amyloid-β proteins at the water/air interface at pH 3 order water inversely^62^ compared to the FUS LC results. Bovine serum albumin shows up-pointing water, similar to the FUS LC segment. Haemoglobin and hydrophobin show positive and negative OH stretching signals at pH values above and below their isoelectric points, respectively^73,75^. As such, there is no general response for water interacting with proteins at the water/air interface. In general, one can state that the water orientation is determined by charged groups exposed to water and non-electrostatic specific interactions between water and the protein.

### Robustness of FUS LC films at the air/PBS buffer interface

**Fig. 4:**
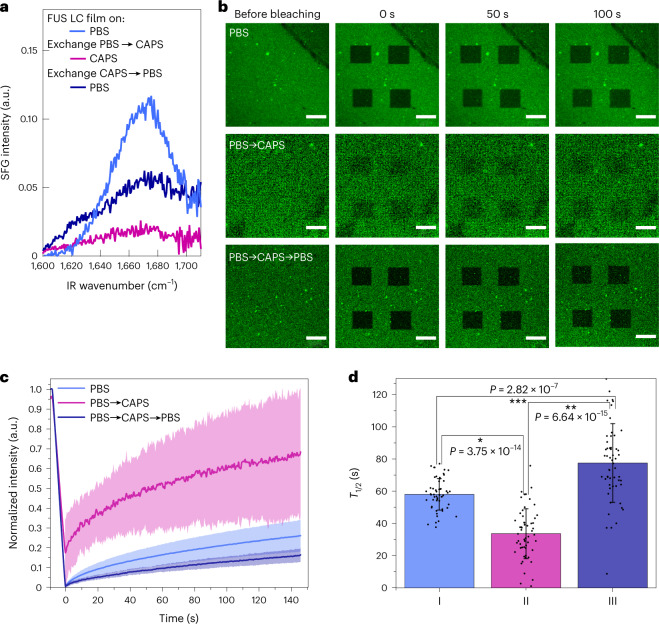
Robustness of the FUS LC film against buffer exchange. **a**, SFG spectra acquired in the amide I region for 1.5 μM FUS LC in PBS (blue), after PBS buffer exchange to CAPS pH 11 (purple) and after subsequent buffer exchange of CAPS back to PBS (dark blue). **b**–**d**, FRAP experiments for the 5% Cy3‐labelled 5 μM FUS LC domain and quantitative analysis. **b**, Representative FRAP images for buffer-exchange experiments. Note the consistent inability to completely bleach the CAPS-exchanged sample, implying high mobility. Scale bars, 20 μm. **c**, Normalized FRAP recovery of the film after formation in PBS buffer (blue), after buffer exchange to CAPS buffer (purple) and after further buffer exchange back to PBS buffer (dark blue). Each data line in **c** was averaged over *N* = 56 unique curves (four samples were prepared and, for each sample, four FRAP measurements were conducted with four bleaching regions of interest). The dark lines show the mean, and the shaded regions are standard deviations. **d**, Bar graphs showing the averaged time of fluorescence recovery time constant *T*_1/2_, inferred from the data in **c**. Bars show the mean, and the error bars are standard deviations. Asterisks show statistical significance, *P* < 0.05, using a two-way analysis of variance followed by a Student’s *t*-test between different groups. I, II and III denote the three experimental stages: PBS, PBS → CAPS and PBS → CAPS → PBS, respectively. Source data

To explore the robustness of the FUS film at the air/buffer solution interface, we performed SFG, SP and FRAP experiments in situ while performing buffer exchanges between PBS and CAPS (experimental details are provided in Supplementary section XIX). We initially formed the FUS LC film at the air/PBS solution interface. Following exchange of the subphase to pH 11 CAPS via a fivefold volumetric exchange, we observed that the SP increased, despite no additional protein being added (Supplementary Fig. 23). This result demonstrates that the film, once formed, is stable even when the buffer condition is reverted to that which fully stabilizes monomeric FUS LC in solution bulk. In addition to the SP increase, we found that the SP fluctuations apparent for the FUS LC film on PBS were considerably attenuated (Supplementary section XX). Together, these changes in SP metrics can indicate that the nature of the FUS LC film changed after the buffer was exchanged to pH 11 CAPS. Along with this finding, we observed that the amide I SFG signal decreased substantially (Fig. 4a) after buffer exchange to pH 11. The SFG and SP results indicate that (1) FUS LC remains at the interface even at high pH and (2) a structural rearrangement of the protein at the interface probably occurs because of the pH increase in CAPS buffer. We hypothesized that FUS LC transforms from an ordered, solid-like structure at the PBS interface to a more liquid-like and disordered state after CAPS buffer exchange. To test our hypothesis that the FUS LC film liquifies after PBS → CAPS buffer exchange, we used FRAP measurements. For the FRAP acquisitions we mirrored the experimental conditions used for the buffer-exchange SFG and SP experiments. FRAP images and fluorescence recovery curves for the original film at PBS and that after the CAPS buffer exchange are presented in Fig. 4b and 4c, respectively. Our data demonstrate that the molecular motion of labelled FUS LC in the film with a CAPS subphase is substantially faster than that in the initial PBS film. This is apparent from three observations: (1) the recovery time constant (*T*_1/2_) decreases from ~60 s for the initial FUS LC film in PBS to ~35 s for the FUS LC film in CAPS, and this difference is statistically robust (*P* < 0.05; Fig. 4d); (2) the inability to fully bleach the film, evident from Fig. 4b and 4c after PBS → CAPS exchange; (3) the protein mobile fraction increases (Supplementary Fig. 28). Together, these data clearly indicate the substantial mobility of the protein and the ‘liquification’ of the film introduced by CAPS. These FRAP data are consistent with, and present proof that, the film shows more mobility after exchange to CAPS compared to the originally formed PBS film.

Subsequently, we exchanged the buffer from CAPS back to PBS (again, without adding any protein). Interestingly, the film is persistent at the interface, as confirmed by SP measurements (Supplementary Fig. 23), and the SFG signal in part recovers in intensity (Fig. 4a). Moreover, we further measured the FRAP recovery after buffer exchange into PBS again, and the recovery time again increased to more than 80 s, which was again statistically significant. It is very intriguing that the SFG amide I intensity follows the same trend: from substantial in PBS, to depleted in PBS → CAPS exchanged, to partially recovered after exchange of the subphase back to PBS. This observation underlines the tight connection between the order of FUS LC at the molecular scale and the suppressed dynamics of molecules in the FUS LC film assembly.

The appearance of the spectral feature at ~1,620 cm^−1^, after the buffer was exchanged back to PBS, is notable. To achieve insight into the origin of this resonance, we performed complementary FTIR measurements on interfacial FUS LC films at all three stages of the buffer exchange (experimental conditions and details are provided in Supplementary section XXII). These data show that the SFG response at 1,620 cm^−1^, evident after the PBS → CAPS → PBS exchange, indeed originates from FUS LC (Supplementary Fig. 29). The amide SFG spectra of the original PBS film and the one formed after buffer exchange to CAPS and returning to PBS show that the film is structurally different at the molecular scale, with the 1,620-cm^−1^ shoulder indicating a more classical amyloid behaviour for the latter.

Summarizing, our data indicate that a film prepared on a PBS subphase appears to liquify when the subphase is changed to CAPS, which solubilizes the FUS LC protein. We note that purified FUS LC (1–214) formed a thermally reversible hydrogel that eventually formed an irreversible condensed fibril assembly after repeated thermal cycling^76^. Similarly, our recent work has shown that bulk, kinetically trapped FUS LC condensates, which are more solid-like than canonical droplets, could be thermally liquified and transformed into liquid droplets^41^. Together, these examples support the notion that a solid-like structure can be converted into a liquid-like structure under proper conditions. Moreover, our data on in situ measurements of FUS LC film robustness show that exchanging the buffer is indeed a tool to manipulate the FUS LC interfacial film state from more solid-like to liquid-like, and back.

Under physiological conditions, intrinsically disordered FUS LC adsorbs strongly to the air/water interface at bulk concentrations as low as 150 nM, more than 600-fold less than required for bulk LLPS. Following interfacial adsorption, disordered FUS LC self-assembles into a solid-like fibrillar network at pH 7.4 and shows molecular-scale order at the air/buffer solution interface. FUS LC surface adsorption and the subsequent organization into a fibrillar network are driven by favourable protein–protein interactions in the presence of a hydrophobic phase (air). The strong response of FUS LC to the presence of hydrophobic surfaces suggests an alternative mechanism underlying the self-assembly and phase separation compared to that in bulk solution. Our findings are consistent with previous work showing that agitation induces FUS LC fibril formation, possibly via interfacial protein fibrillar species getting mixed into bulk when the interface is disturbed^40^. Similarly, other proteins, such as α-synuclein, have been shown to co-localize to the surface of lipid droplets, emulsified hydrophobic phases in the cytosol^77^. Interestingly, FUS LC film formation was sufficient to reverse the water molecule orientation. Finally, we found that the FUS LC film was stable in response to buffer exchange into conditions that stabilize monomeric FUS LC, highlighting the persistent nature of the FUS films. This knowledge provides crucial insights into the formation of solid-like aggregates from otherwise liquid-like condensates, and the presence of a macroscopic hydrophobic surface allows us to get mechanistic insights into this process that are otherwise inaccessible. Recognizing the importance of hydrophobic interactions suggests potential ways of tuning the nature of phase-separated FUS with targeted therapeutics to limit protein aggregation in neurodegenerative diseases.

## Methods

### Protein expression and purification

Plasmid used for FUS LC expression was obtained from AddGene (plasmid 127192). Human FUS LC (amino-acid residues 1–163) was expressed in a chemically competent *Escherichia coli* bacterial strain. Cells were grown in LB medium containing kanamycin shaken at 37 °C until an optical density at 600 nm within 0.6–1 was reached. Expression was induced by the addition of isopropyl-β-d-thiogalactoside (IPTG) to a final concentration of 1 mM. After 4 h of IPTG induction, the cells were centrifuged at 4,500*g* for 10 min at 4 °C. The resultant pellet was stored at −80 °C. For the cell lysis, the pellet was redispersed in 20 ml of phosphate buffer at pH 7.4 (containing 300 mM NaCl and 10 mM imidazole) and followed by sonication in an ice bath. Lysed cells were sedimented by centrifugation at 18,500*g* for 1 h at 4 °C (using an Eppendorf centrifuge, 5810R). The obtained pellet was redispersed in solubilizing buffer (phosphate buffer pH 7.4 containing 300 mM NaCl, 10 mM imidazole and 8 M urea) and stirred overnight at 4 °C. The sample was centrifuged again at 18,500*g* for 1 h at 4 °C, and the supernatant was loaded to Ni-NTA agarose resin-containing columns. After binding of histidine-tagged protein to Ni-NTA (for 1 h at 4 °C), the unbound proteins and cell fragments were washed several times with phosphate buffer (pH 7.4) containing 300 mM NaCl and 5 mM imidazole. The protein of interest was subsequently eluted in steps by running through the washing buffer with increasing imidazole concentration (10, 20, 40 and 100 mM). Purified protein was cleaved by diluting in solubilizing buffer with the addition of tobacco etch virus protease with a 1:20 mass ratio. Cleaved purified protein was buffer-exchanged to CAPS (pH 11) overnight, then concentrated using a 3-kDa Amicon filter and stored at −80 °C.

### Vibrational SFG spectroscopy

SFG is a surface-sensitive second-order nonlinear spectroscopic technique that allows selective probing of vibrational modes of molecules at interfaces^78^. SFG is extensively used to study biointerphases^51^.

#### Homodyne SFG spectroscopy

Homodyne SFG spectroscopy experiments were performed on a laser system consisting of a Nd:YLF pump laser (Empower 45, Spectra Physics), a Ti:sapphire seed laser (MaiTai, Spectra Physics) and a regenerative amplifier (Spitfire Ace, Spectra Physics). The Spitfire Ace output (~5-mJ power, 1-kHz repetition rate, ~800-nm centre wavelength) is separated into two by a beamsplitter. The first part pumps an optical parametric amplifier (OPA, TOPAS-C, Light Conversion). The OPA, conjugated with the difference frequency generation (DFG) stage, produces a broadband infrared (IR) beam (3–6 mW) with tunable centre frequency. The second part is directed into a home-built pulse shaper consisting of a grating, a plano-convex cylindrical lens, a slit with a tunable width and a dielectric mirror. The pulse shaper allows us to generate a narrowband visible pulse with a bandwidth of ∼10 cm^−1^. The visible and IR beams are further focused and tuned to enable spatial and temporal overlap at the sample surface with incident angles of 64° and 40°, respectively. The generated SFG beam is collimated, directed into the detection path, focused, further directed into and dispersed by the spectrometer (Andor SR-303i-A), and finally detected by an electron-multiplied charge-coupled device (CCD camera, Andor Newton). The polarization state of each beam (SFG, visible and IR) is controlled by a polarizer and a half-wave plate. In our experiments, the ssp (s (SFG), s (visible), p (IR)) polarization combination was used, where p (s) denotes the polarization parallel (perpendicular) to the plane of incidence. SFG spectra were recorded using Andor Solis software. A *z*-cut quartz crystal was used as a reference. SFG spectra acquisition for the sample and reference was followed by a background acquisition by blocking the IR beam. For the processing, a background-corrected sample SFG spectrum was divided by a background-corrected reference SFG spectrum.

#### HD-SFG spectroscopy

HD-SFG^71^ measurements were performed in a collinear beam geometry using a Ti:sapphire regenerative amplifier (centred at 800 nm, ∼40-fs pulse duration, 5-µJ pulse energy, 1-kHz repetition rate, Spitfire Ace, Spectra Physics). Part of the output was used to generate a broadband IR pulse in an OPA (TOPAS-C, Light Conversion) with a DFG crystal. The other part of the output was directed through a pulse shaper consisting of a grating-cylindrical mirror system to generate a narrowband visible pulse with a bandwidth of ∼10 cm^−1^. The IR and visible beam were first focused into a 20-μm *y*-cut quartz plate as the local oscillator (LO). These beams were collinearly passed through a 2-mm SrTiO_2_ plate for phase modulation and focused on the sample surface at an angle of incidence of 45°. The SFG signal from the sample interfered with the SFG signal from the LO, generating the SFG interferogram. The SFG interferogram was dispersed in a spectrometer (HRS-300, Princeton Instrument) and detected by a liquid nitrogen-cooled CCD camera (PyLoN, Princeton Instruments). The data were analysed using a previously described procedure^79^. Briefly, the complex spectra of the second-order nonlinear susceptibility ${\chi }_{\rm{eff}}^{(2)}$ were obtained via Fourier analysis of the interferogram and normalization by a *z*-cut quartz crystal. All measurements were performed using the ssp polarization combination.

### SP measurements

We used SP measurements to study the protein interfacial adsorption, using a DeltaPi tensiometer (KBN 315 Sensor Head, Kibron Inc.) and FilmWareX 3.62 software. The SP data were recorded versus time.

SFG and SP experiments were performed simultaneously. The trough was cleaned with ethanol (Sigma Aldrich, absolute, ≥99.8%) and then with water (Millipore Milli-Q, resistivity of 18.2 MΩ cm). Except when otherwise noted, the trough was filled with 4.8 ml of PBS buffer (Dulbecco’s PBS 1X, Gibco). The SP was also calibrated using pure water. The concentrated FUS LC protein solution (200 μl of protein dissolved in CAPS buffer pH 11) was injected into the subphase with a glass syringe to reach a final protein bulk concentration of 1.5 μM. The sample was left to equilibrate until the SP stabilized. Afterwards, SFG spectra were collected until no further changes in the spectrum could be observed. During the entire experiment, the sample box was flushed with nitrogen to prevent absorption of the IR beam by water vapour. The resultant relative humidity in the sample box was less than 5%, which produces a highly hydrophobic environment inside the measurement chamber above the buffer solution (Supplementary section II provides details about the humidity). Note that the sample trough was rotated, allowing us to probe various spots at the sample surface and avoid possible depletion of molecules from the laser focus area.

### BAM imaging

BAM imaging was performed on an Accurion instrument (Nanofilm EP3) with EP3 View software. A ×10 objective and a white Teflon trough were used. The objective focus, the polarization optics settings (angles of the polarizer, compensator and analyser), the sample stage height, the laser power and the laser beam incidence angle were adjusted to optimize the image contrast and the signal-to-noise ratio. For each experiment, 200 μl of FUS LC was added into 3.8 ml of the subphase (the FUS LC concentration in the subphase was 1.5 μM). The sample was left to equilibrate, and BAM images (dimensions of 387 μm × 500 μm) were acquired every hour after the addition of FUS LC to the subphase.

### FUS LC film deposition on the solid substrate for AFM studies

A freshly cleaned Si wafer was placed into a home-designed and home-built Teflon trough (volume 5 ml). We note that the trough bottom was created tilted at an angle of 8° with respect to the horizontal plane to provide effective deposition of the protein film on a solid substrate upon liquid subphase removal. The trough was filled with PBS buffer, submerging the Si wafer, and protein was added into the subphase to reach a final protein concentration of 1.5 μM. The sample was left to equilibrate for 5 h. Afterwards, the solvent was gently removed with a Hamilton syringe without disturbing the Si wafer, then the wafer was dried overnight for subsequent AFM imaging in peak-force-mode Bruker Dimension Icon probes: OLTESPA with a nominal resonance frequency of 70 kHz and a nominal spring constant of 2 N m^−1^. All experiments were conducted at 20 ± 1 °C.

### FRAP

FUS LC was labelled according the protocol described in Supplementary section XXIII. Cy3-NHS ester dye (1 mg, Lumiprobe) was dissolved in 100 μl of dry dimethylformamide and divided into aliquots of 40 μl. FUS LC (50 µM) in HEPES buffer (50 mM, pH 8.0) was added to 40 µl of dye stock solution in dimethylformamide. The reaction mixture was incubated overnight at 4 °C. Purification of dye-labelled FUS LC was carried out by repeating buffer exchange to CAPS (pH 11) at 4 °C to remove excess dye, and then concentrated using a 3-kDa Amicon filter and stored at −80 °C. For FRAP experiments on droplets, 0.03 mol% of Cy3-labelled FUS LC was doped into 300 µM unlabelled FUS LC droplets. For the film experiment, a glass-bottom 35-mm dish (Matek, #1.5 coverslip) was used. The dish was initially filled with 1,982 µl of PBS, and 18 µl of FUS LC solution (594 µM) in 20 mM CAPS pH 11 was injected into the PBS solution to make a total volume of 2 ml. Five percent of injected FUS LC monomers were labelled with Cy3 as described above. The final concentration of FUS LC in the dish was 5 µM. The dish was covered, transferred to the microscope stage, and incubated for 2 h at 25 °C to allow for FUS LC film formation. FRAP was performed on a Leica SP8 confocal microscope with ×20, 0.95 NA water immersion objective and 532-nm laser line. All buffer exchanges were done with two 1-ml plastic syringes equipped with 25-G needles. One syringe was used to add 1 ml of new buffer (gently) at the bottom of a dish, and the other needle was used to remove 1 ml. This cycle was repeated ten times for a total of 5× volume replacements with the target buffer. The buffer change from PBS → CAPS was done ~3 h after film formation, and the following buffer change from CAPS → PBS was done after another 3 h. FRAP data were processed using EasyFRAP with the ‘Full normalization’ process. Each bleached region of interest was fit independently, four measurements were taken per film condition, and four independent samples for each buffer setting were prepared, for a total of *N* = 56 curves for each condition.

## Online content

Any methods, additional references, Nature Portfolio reporting summaries, source data, extended data, supplementary information, acknowledgements, peer review information; details of author contributions and competing interests; and statements of data and code availability are available at 10.1038/s41557-023-01221-1.

## Supplementary information

Supplementary Information Supplementary Figs. 1–29, Tables 1 and 2 and methods.

## Data Availability

The data that support the findings of this study are provided at 10.17617/3.PUBRKR. Raw images for FRAP analysis can be obtained by reasonable request from the corresponding authors. Source data are provided with this paper.

## Source data

Source Data Fig. 1 SP traces, AFM images, BAM images.
Source Data Fig. 2 FRAP analysis of droplet and film for Figs. 2 and 4c,b,d.
Source Data Fig. 3 Homodyne and heterodyne SFG traces.
Source Data Fig. 4 Homodyne SFG traces PBS, PBS→CAPS, PBS→CAPS→PBS.
