# Supplementary material for: Fibril formation and ordering of disordered FUS LC driven by hydrophobic interactions
Source: Nat Chem. 2023 May 25;15(8):1146–54. doi: 10.1038/s41557-023-01221-1 (PMC10396963; doi:10.1038/s41557-023-01221-1)
Supplement: Supplementary file 1 — Supplementary Figs. 1–29, Tables 1 and 2 and methods. [file 41557_2023_1221_MOESM1_ESM.pdf]

nature chemistry

Article

https://doi.org/10.1038/s41557-023-01221-1

Fibril formation and ordering of disordered FUS LC driven by hydrophobic interactions

In the format provided by the authors and unedited

Supporting Information (SI)

Table of Contents:

I. Experimental scheme for SFG and SP studies of lipid monolayer/protein interactions............S4

II. Humidity: Experimental conditions of SFG and SP measurements.......................................S4

III. CAPS molecule in pH 11: Control experiments on interfacial behavior.................................S6

IV. Surface pressure (SP) fluctuations: A control experiment with the trough rotation switched off..S9

V. Brewster angle microscopy (BAM) images for 1.5 μM FUS LC at the air/PBS buffer interface..S10

VI. BAM images for 0.15 μM FUS LC at the air/PBS buffer interface.......................................S10

VII. BAM images for 1.5 μM FUS LC at the air/CAPS buffer interface.......................................S11

VIII. FUS LC fibril size..S11

IX. Time-dependent SFG spectra for FUS LC film at the air/water interface............................S12

X. SP and SFG data for 1.5 μM FUS 12E LC *vs.* 0.15 μM FUS LC at the air/PBS buffer interface..S13

XI. SFG spectra for FUS LC at the air/PBS buffer interface at different FUS LC bulk concentrations..S13

XII. SFG spectra for FUS LC at the air/PBS buffer interface at different local, but fixed bulk concentration...S16

XIII. FTIR measurements of the FUS LC film..S17

XIV. SFG signal in amide II spectral region...S20

XV. FUS LC interaction with PC and TAP membranes investigated by SFG and SP..S22

XVI. Homodyne SFG spectra in the CH-/OH-stretching region for 1.5 μM FUS LC at the air/PBS buffer interface..S23

XVII. Control experiments: HD-SFG of FUS LC in CAPS pH 11, and HD-SFG of 0.8 mM CAPS in PBS....S25

XVIII. $\chi^{(3)}$ analysis of the SFG spectra in the OH-stretching region for 1.5 μM FUS LC at the air/PBS buffer interface....S28

XIX. SP data for PBS→CAPS→PBS buffer exchange experiment....S31

XX. Analysis of SP fluctuations for PBS→CAPS→PBS buffer exchange experiment....S32

XXI. Protein mobile fraction during buffer exchange....S36

XXII. FTIR experiments on FUS LC film upon buffer exchanges....S37

XXIII. FUS LC Labelling Protocol....S39

References....S40

I. Experimental scheme for SFG and SP studies of lipid monolayer/protein interactions.

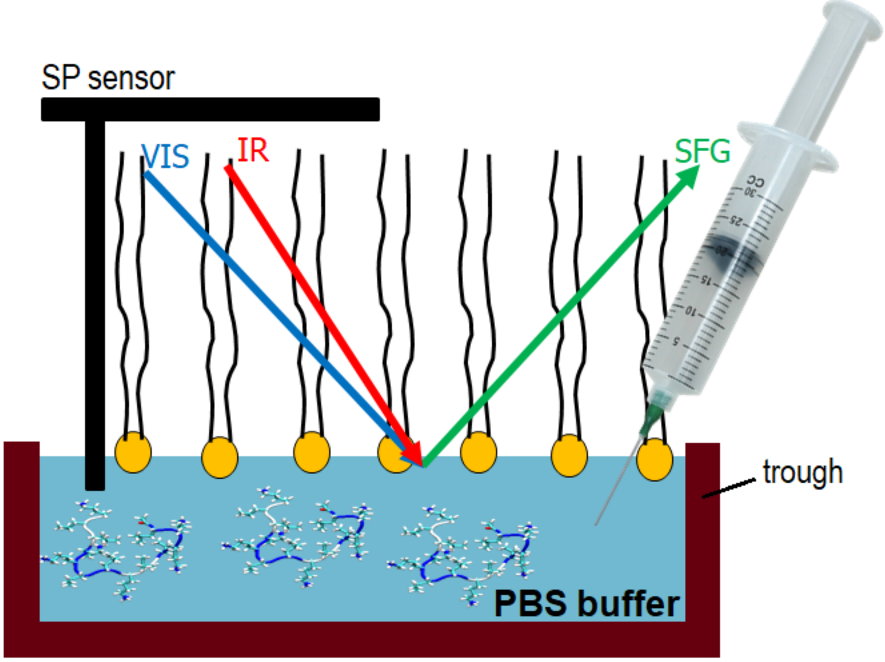

Figure S1. Experimental scheme to study lipid/protein interactions: the trough is filled with the PBS buffer, the probing needle of the SP sensor calibrated at the air/water interface is inserted into the subphase, lipid monolayer used as a model biomembrane is spread at the air/buffer interface, SFG is typically measured before and after addition of a protein solution into the subphase.

II. Humidity: Experimental conditions of SFG and SP measurements.

In the experimental enclosure, we flush with nitrogen continuously and measure the humidity with the temperature/humidity sensor "EBI 20-TH1 Standard-Temperatur-/Feuchtedatenlogger". According to the technical information, the systematic error is (depending on the relative humidity (RH) value, in brackets): ±3% RH (30% RH ... 90% RH), ±5% RH (10% RH ... 30% RH). As we see, below 10% RH, the value is actually in the range of 0-10% RH. However, the more important factor for us is not the exact humidity value, but rather the ability to provide experimental conditions to sufficiently reduce water IR absorption bands that would "contaminate" an SFG spectrum. The water bending mode, in particular, would complicate the interpretation of the amide I region (see Figure S2). The nitrogen flushes out water vapor and thus allows us to suppress water absorption bands. In this sense, the amplitude of water bands acts like an *in situ* humidity sensor. In our experimental setup, we almost completely remove water bands, which puts an upper bound on the RH of <5%.

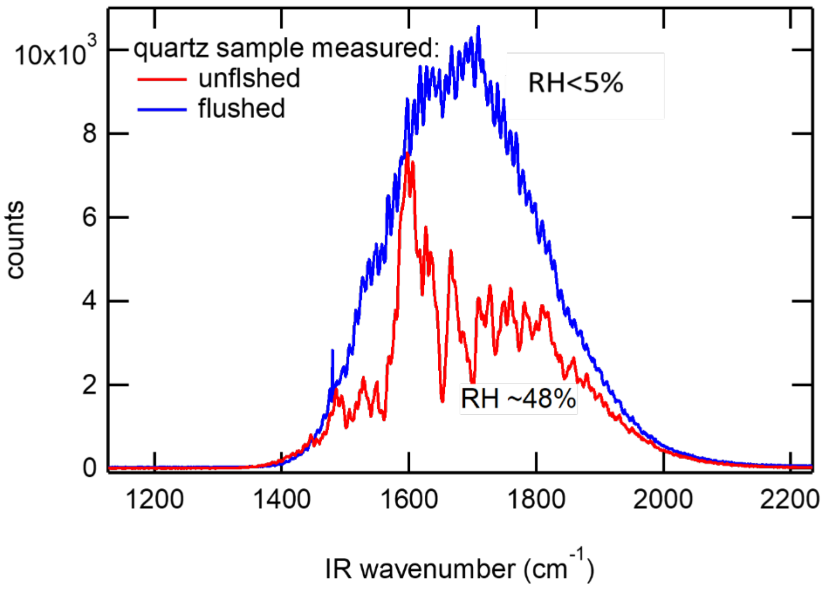

Figure S2. Non-resonant signal from z-cut quartz sample at (red) unflushed vs. (blue) flushed condition in OH bending / amide I region. In the red curve, the dips in spectrum correspond to the water absorption lines. Nitrogen flushing allows to get rid of water absorption bands, as clear from blue curve, from which we infer an RH less than 5%.

As an additional demonstration of the concept, we present an experiment where we record nonresonant SFG signal from z-cut quartz while varying humidity in the sample box. At each humidity value, we record both SFG signal and background (by blocking the IR beam), subtract background from the signal (and thus get the background-corrected signal). Then for different RH from the sensor, the background corrected spectrum at ~0% humidity (completely purged) is subtracted from the background-corrected spectrum recorded from each RH. We perform the experiment in the free OH region (peak centered at ~3750 cm^{-1}), since the water band structure is stronger and easier to interpret compared to OH bending. In this way, we measure the water band intensity at each RH value and plot the number of counts at the main peak centered at ~3760 cm^{-1} versus the RH value (Figure S3). Note that we added (0;0) point as a natural “boundary” condition (no water vapor = no bands). A linear correlation between the water band amplitude and RH measured by humidity sensor is evident from the graph.

Since we flush the sample box with nitrogen in such a way that no water bands can be observed in the spectrum, the RH is <5% can be empirically determined with a good level of precision, although the systematic error of the sensor not reported in that range.

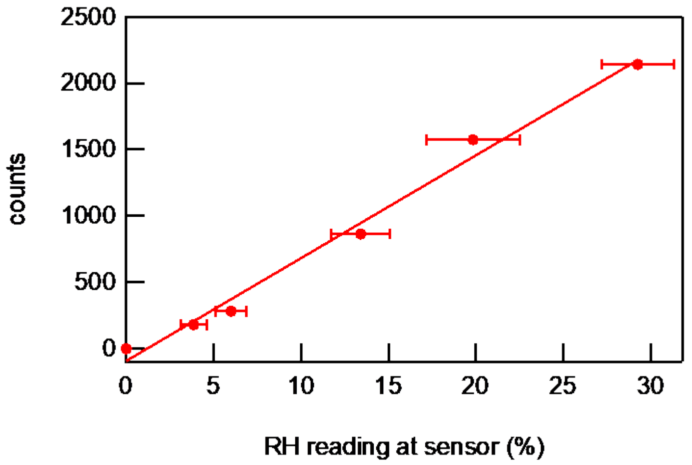

Figure S3. Amplitude of water band measured in number of counts versus RH value obtained from humidity sensor. The red line presents a linear fit. Horizontal error bars are standard deviation of RH reading recorded by three humidity sensors placed in the sample box. The Y error bars are not presented, as they are statistically negligibly small.

III. CAPS molecule in pH 11: Control experiments on interfacial behavior.

The CAPS buffer at pH 11 was used to stabilize FUS LC molecules in the monomer state as outlined in by Burke *et al.*[1] To the best of our knowledge, there have not been any studies reporting the interfacial behavior of CAPS molecule. Thus, we have performed additional surface-specific experiments for the conditions relevant to our work.

In Figure S4, we present SP measurements. The SP is first calibrated to the air/PBS buffer interface (at t~0s), and then the SP measurement is started. At time t~100s, the trough with PBS buffer solution is carefully drained, and the SP probing needle becomes free (i.e. out of the sample). As the definition of SP is $\pi = \gamma_0 - \gamma$, the surface tension $\pi = \gamma_0 {\sim} 72.5$ mN/m for the needle out of the sample, exactly what we observe. At t~180s, we carefully fill the trough with 20 mM CAPS pH 11 buffer to immerse the end of needle into the liquid, as marked with an arrow in Figure S4. For our measurement, we use a height sensor (Keyence LK-G82 Lasersensor, precision ±1 μm) to keep the sample height constant, thus eliminating the effect of a different depth of needle immersion after the sample exchange. We observe a ~ 1.5 mM/m change in surface pressure when changing the PBS buffer to CAPS. Therefore, we conclude that the minor population of CAPS molecules is present at the interface and change the SP only slightly (in the range of ~1.5-2 mN/m) compared to PBS.

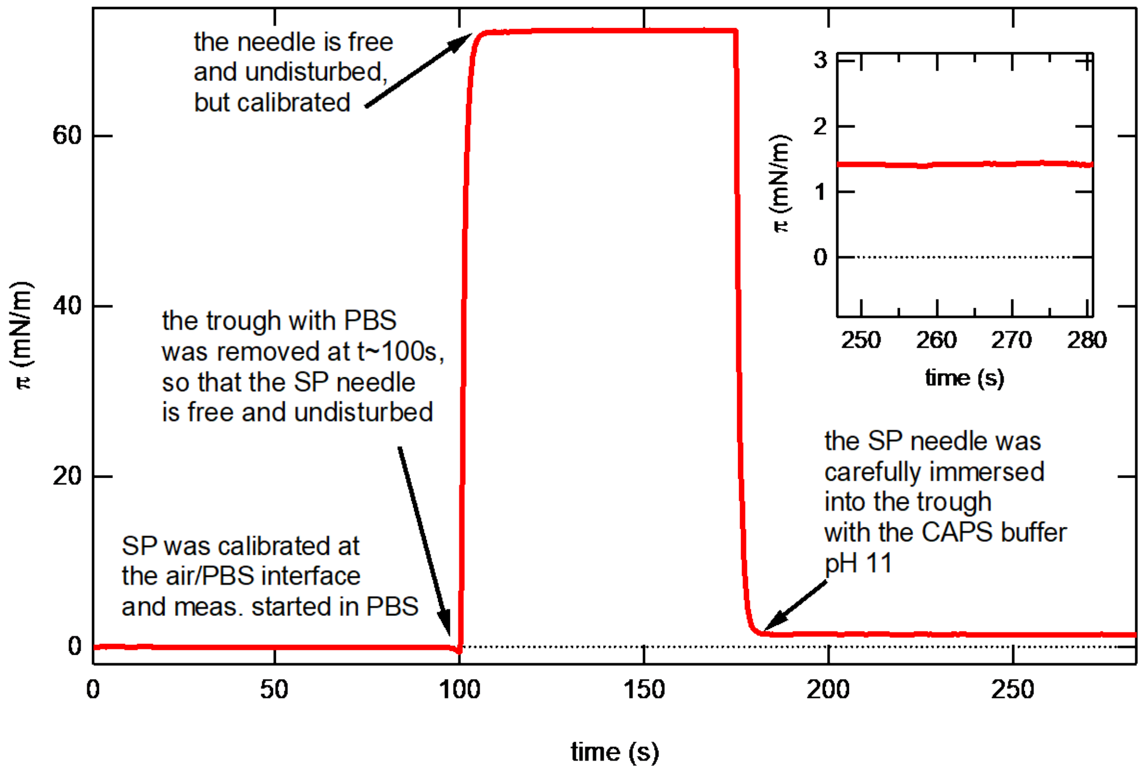

Figure S4. Surface pressure over time upon change of sample from PBS to CAPS. The increase up to 72.5 mN/m happens when the SP needle loses contact with the liquid surface (at t~100s) and is almost back to the value of SP~1.5 mN/m (see inset) for the air/liquid interface of the CAPS buffer (insertion of a needle to the CAPS sample is at ~180s).

In addition to SP measurements through complete buffer exchange, we doped CAPS into a PBS subphase at precisely the same dilution, the same as was done when introducing monomeric FUS LC in the SFG experiments. The SP measurement was started at t~0s (see Figure S5), and at times marked with black arrows, 100 μL of CAPS buffer solution (20 mM in miliQ, pH 11) was added with a glass syringe into the sample trough (no protein was added), so that the final CAPS concentration in the trough was 0.8 mM, exactly same as in our experiments with FUS LC. At t~330s (marked with a green arrow), we have switched the trough rotation on to ensure the good mixing of the sample and to mirror the experimental conditions for the experiments with FUS LC injection. In summary, the changes of SP after CAPS injection are negligible. The change in surface pressure is less than 0.1 mN/m, i.e. less than 1% of the changes seen with the interfacial FUS LC film (15-20 mN/m).

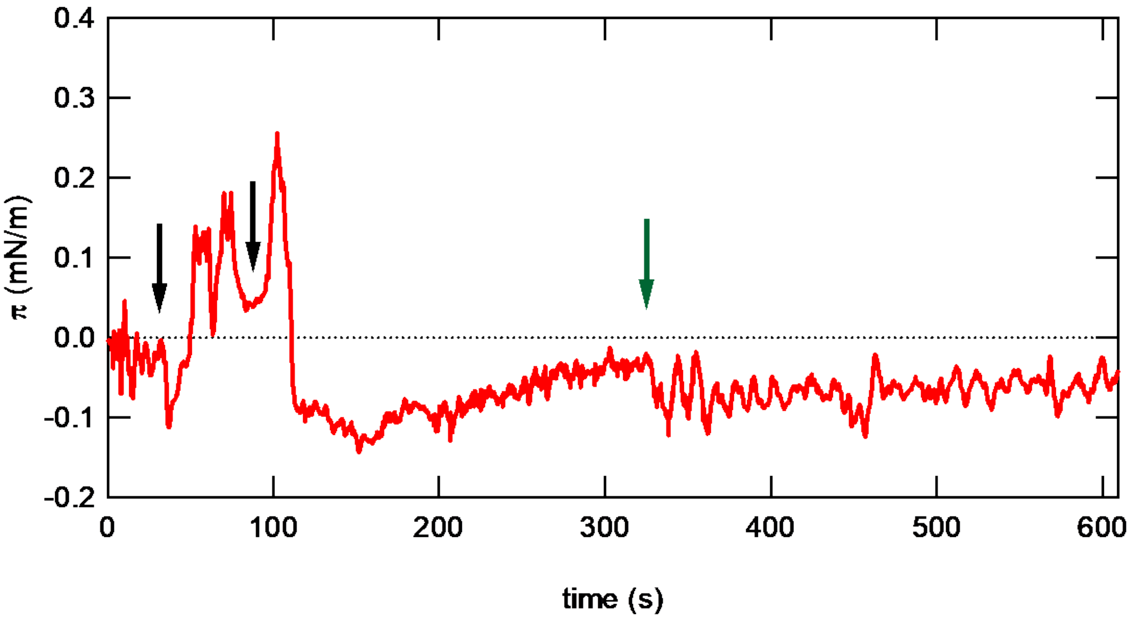

Figure S5. Surface pressure versus time. Initial subphase is PBS, the black arrows indicate timepoints of CAPS injection (overall 200 nL of 20 mM CAPS pH 11 injected into 4.8 mL PBS), the green arrow indicates time when the rotation of the trough was switched on.

IV. Surface pressure (SP) fluctuations: A control experiment with the trough rotation switched off.

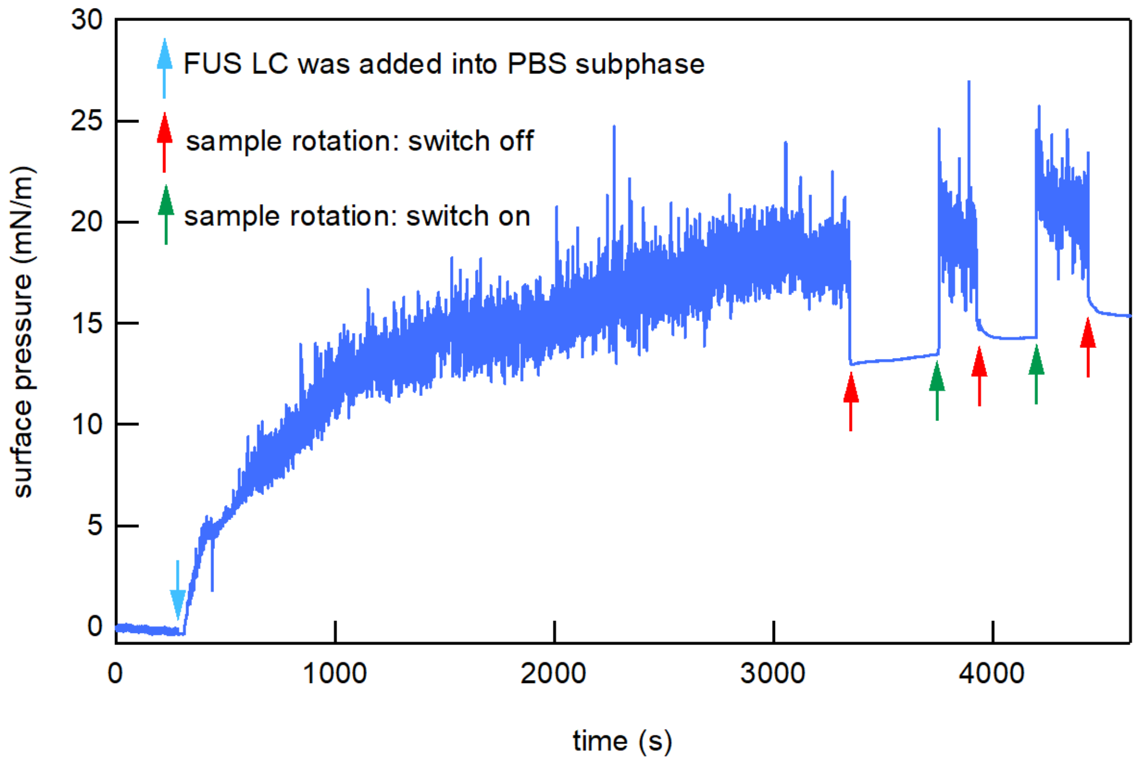

Figure S6. Surface pressure measurement of 1.5 μM FUS LC adsorption at the air/PBS interface (protein injection is marked with a blue arrow) with trough rotation switching off (marked with a red arrow) and back on (marked with a green arrow).

Control experiment reveals the stable surface tension when the rotation of the trough is switched off, see Figure S6. The results indicate a continuous average force (corresponding to a surface pressure of ~5 mN/m) acting on the needle, through the collisions with peptide particulates on the surface, in addition to the fluctuations those collisions cause.

V. Brewster angle microscopy (BAM) images for 1.5 µM FUS LC at the air/PBS buffer interface.

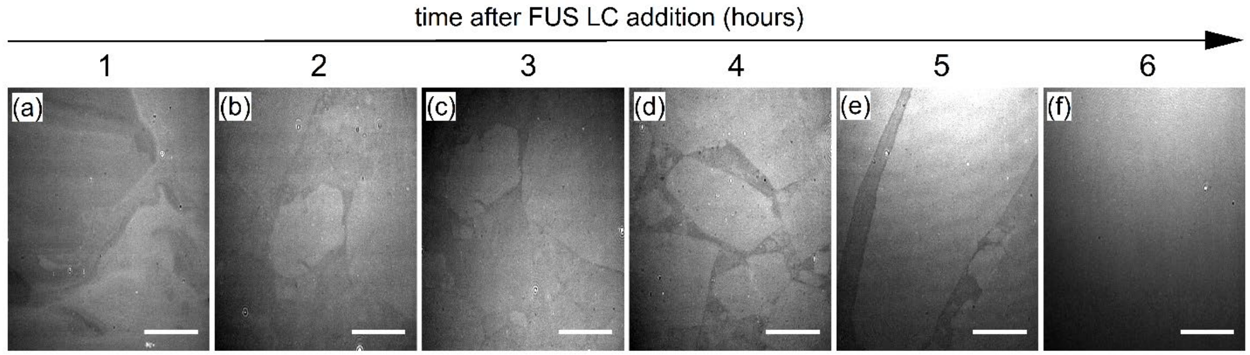

Figure S7. BAM images recorded at one-hour intervals after the addition of 1.5 µM FUS LC in **PBS** buffer (at t=0). Scale bar corresponds to 100 µm.

VI. BAM images for 0.15 µM FUS LC at the air/PBS buffer interface.

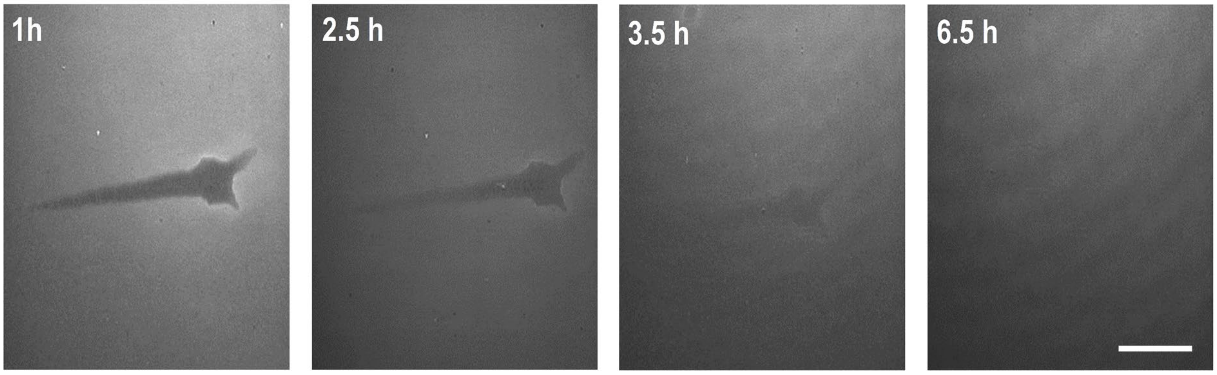

Figure S8. BAM images collected during the film formation and homogenization for **0.15 µM FUS LC in the PBS buffer** (protein addition at t=0). BAM image dimensions are 387 µm x 500 µm. Scale bar is 100 µm.

VII. BAM images for 1.5 μM FUS LC at the air/CAPS buffer interface.

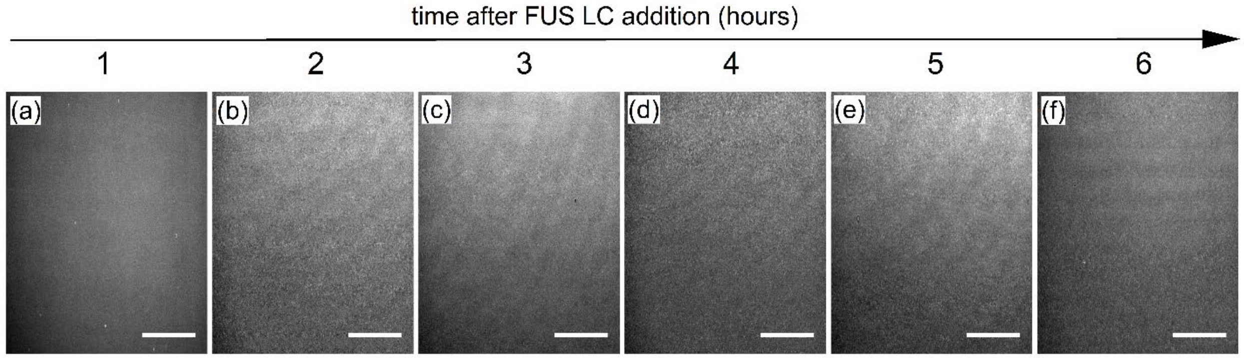

Figure S9. BAM images recorded at one-hour intervals after the addition of 1.5 μM FUS LC in **CAPS** buffer (at t=0). Scale bar corresponds to 100 μm.

VIII. FUS LC fibril size.

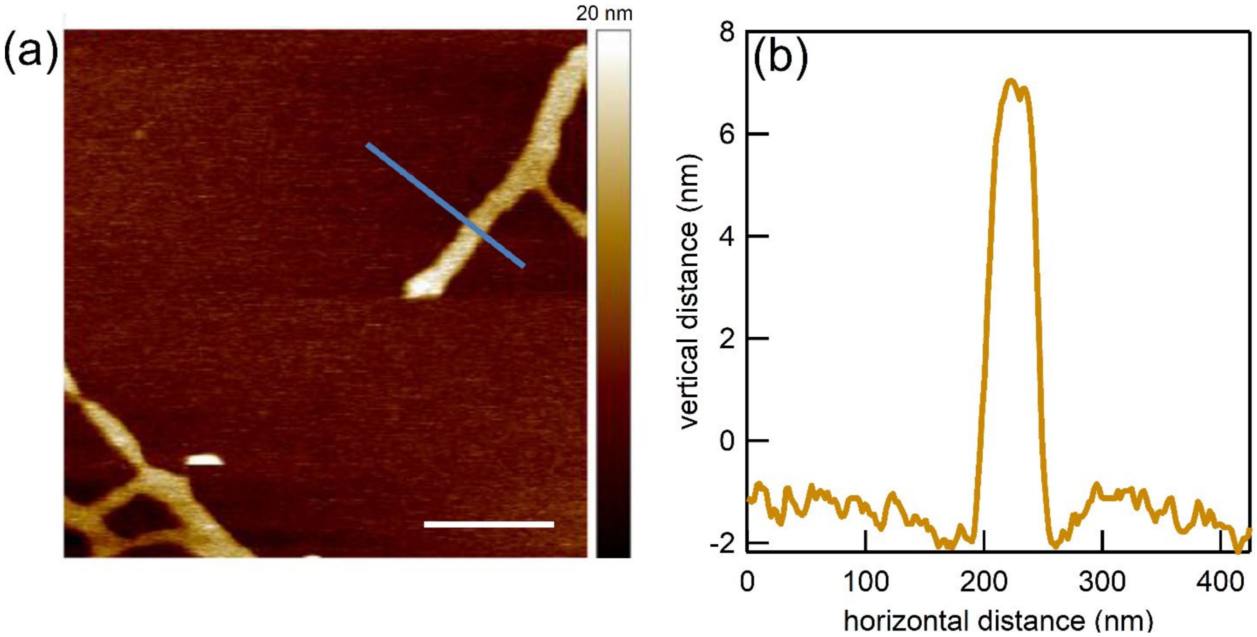

Figure S10. (a) AFM image of the FUS LC protein film formed at the air/PBS buffer interface and deposited on a silicon wafer and (b) height profile of the fibril marked in (a). Scale bar in (a) corresponds to 250 nm.

IX. Time-dependent SFG spectra for FUS LC film at the air/water interface.

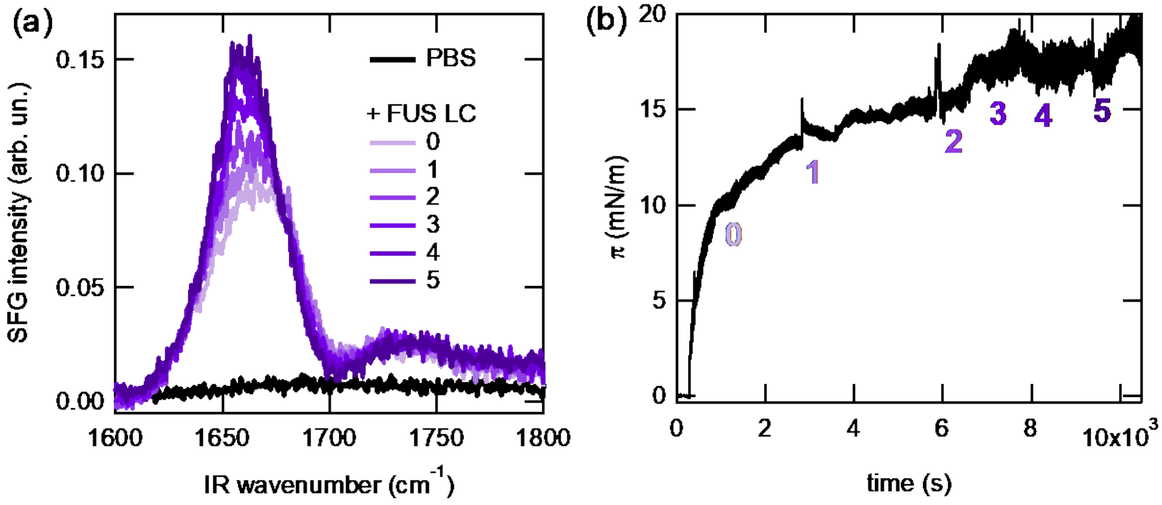

Figure S11. Kinetic SFG spectra of 1.5 μM FUS LC sample in PBS buffer in the amide I and carbonyl stretch region show some spectral intensity changes over time and only insignificant change in the spectral shape.

We record kinetic SFG spectra until no changes in spectral shape and intensity are observed (in average, this is reached in ~2 hours after FUS LC injection into the PBS subphase). Both kinetic as well as steady-state SFG spectra present an amide I band centered at ~1670 cm^{-1} and does not contain any noticeable feature centered at ~1620 cm^{-1}. Because of the kinetic nature of the film formation, it is challenging to determine whether the final state we measure is the true thermodynamic equilibrium state or, rather, a long-lived metastable state. We perform measurements of liquid samples with an open surface, so the effect of evaporation is unavoidable, and this limits the experiment time, especially in comparison with bulk studies of closed samples, e.g. in Eppendorf tubes or 96-well plates. Nevertheless, we can definitively say that on timescales up to ~3.5 hours, we see no additional peaks (including the resonance at 1620 cm^{-1}), but rather only the slight growth of the already-present peak in the SFG response (see Figure S11).

In fact, from our AFM experiments (reported in the main text in Figure 1) and from FTIR data (Figure S16) we can claim: (1) for AFM measurements, the film was allowed to develop at the air-water interface on over a few hours, was transferred to the solid substrate, and fibrils were observed; (2) a comparison of FTIR measurements of the film on the liquid surface with the transferred film revealed a very similar infrared response for the on-liquid vs. on-solid films (Figure S16). Thus, from the statement (2) follows: the two films, i.e. on-liquid and on-solid, are structurally very similar. Finally, from the statements (1)&(2) follows: fibrils were present already on the liquid surface.

X. SP and SFG data for 1.5 μM FUS 12E LC *vs.* 0.15 μM FUS LC at the air/PBS buffer interface.

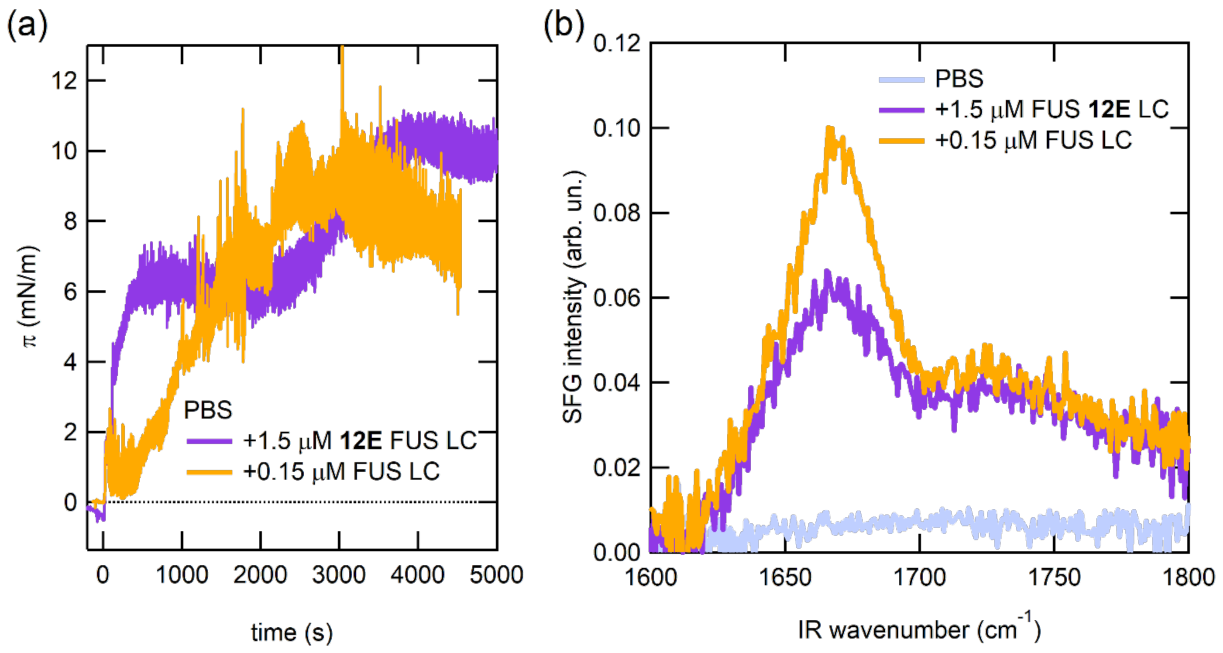

Figure S12.(a) Surface pressure vs time for (yellow) 0.15 μM FUS LC and (purple) 1.5 μM FUS **12E** LC adsorption at the air/PBS buffer interface. (b) SFG spectra in the amide I and C=O stretching region for (yellow) 0.15 μM FUS LC and (purple) 1.5 μM FUS **12E** LC adsorbed at the air/PBS buffer interface.

XI. SFG spectra for FUS LC at the air/PBS buffer interface at different FUS LC bulk concentrations.

We tested the effect of protein bulk concentration on the adsorption and organization of FUS LC at the air/PBS interface. Various final concentrations of FUS LC in PBS were tested, namely 1.5, 3, and 6 μM. No major differences in SP were observed (Figure S13a), all three curves are fluctuating, and only for 6 μM the SP is slightly higher (by ~3 mN/m). Figure S13b shows that the SFG C=O signal is the same for all concentrations while the amide I intensity is different. The spectral shape observed for 6 μM FUS LC is different from that for 1.5 and 3 μM. Fitting is needed to disentangle the contributions from different folding motifs at each protein concentration. Fitting of the amide I mode contribution for the SFG spectra presented in Figure S13b was performed in the frequency region 1615-1705 cm^{-1}. The results are presented in Table S1. For each considered FUS LC concentration, the ratio of the integrated SFG intensity of the peak at ~1671 cm^{-1} (assigned to different β-folded structures)[2–8] to that of the peak at ~1656 cm^{-1} (assigned to the

random-coil and/or α-helix structures)[9] was calculated based on fitting results and is shown in the inset in Figure S13b. As evident from the inset, the intensity of the β-contribution decreases upon increasing the protein concentration. Since protein fibrils are commonly densely packed and enriched with β-folded structures,[10] we assume that the observed trend for the different protein bulk concentration arises from the different the orientation of FUS LC molecules folded into β-conformation at the interface.

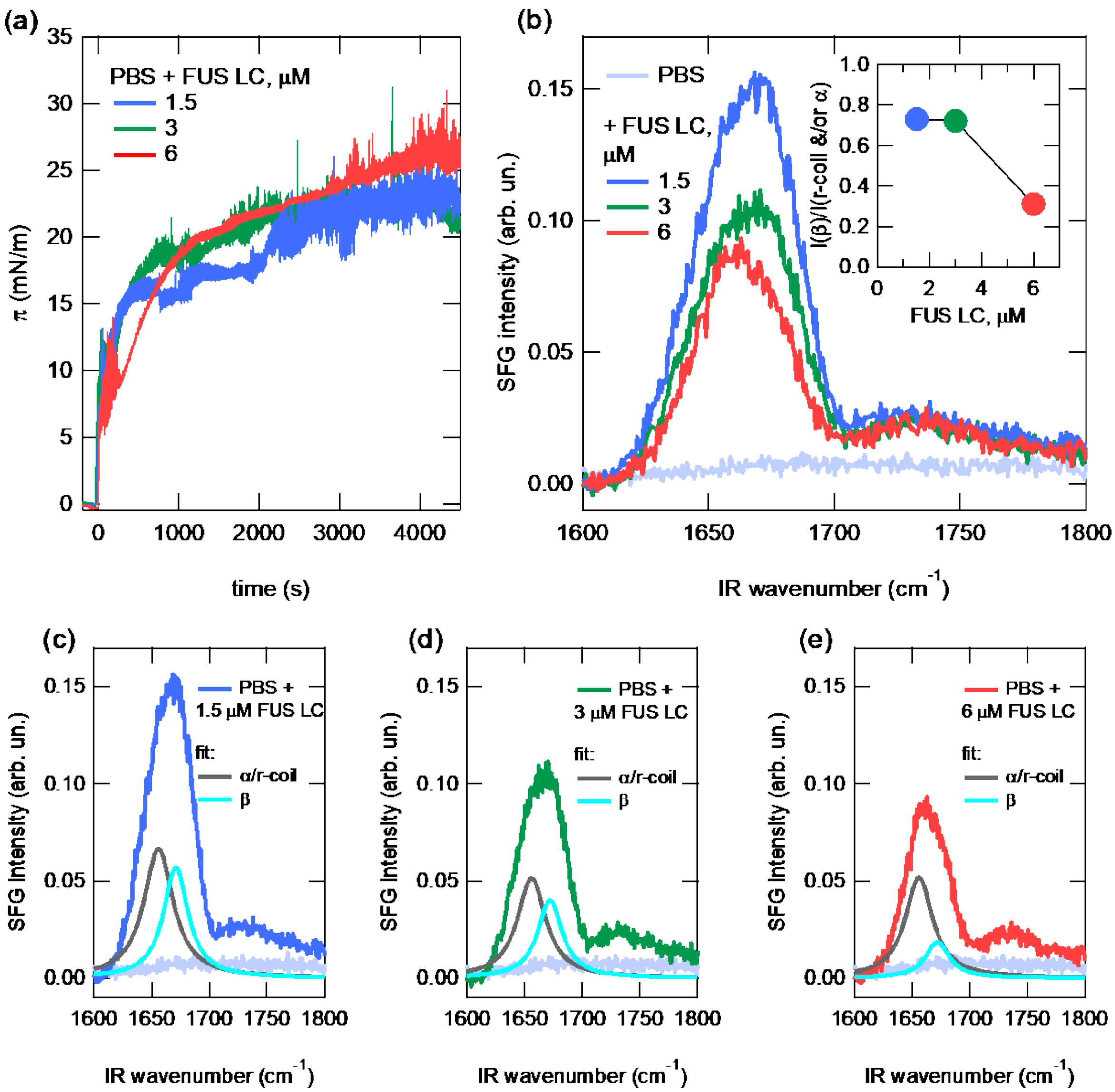

Figure S13. (a) Surface pressure curves for (blue) 1.5 μM, (green) 3 μM, and (red) 6 μM FUS LC in PBS; (b) SFG spectra in the amide I and carbonyl stretching region after equilibration (t~5000s) for (blue) 1.5 μM, (green) 3 μM, and (red) 6 μM, respectively. Inset: the ratio I_{SFG}(β)/I_{SFG}(random-coil and/or α-helix) as obtained from the spectral fitting of the experimental data plotted against the FUS LC concentration in PBS.

(c)-(e) Resonant contributions (see Table S1) in the amide I region presented in graph (b): α-helix/random coil (gray) and β-folded (cyan).

Table S1. Results of the fitting for SFG spectra presented in Figure S13b.

	**1.5 μM**	**3 μM**	**6 μM**
A_{NR}	0.01	0.01	0.01
ϕ_{NR}	-0.1	-0.1	-0.1
$A_{(\alpha\ \text{and/or r-coil})}$	4.0	3.4	3.3
$\omega_{(\alpha\ \text{and/or r-coil})}$	1656	1656	1656
$2\Gamma_{(\alpha\ \text{and/or r-coil})}$	31	30	29
$A_{(\beta)}$	3.1	2.6	1.7
$\omega_{(\beta)}$	1671	1672	1672
$2\Gamma_{(\beta)}$	26	26	25

XII. SFG spectra for FUS LC at the air/PBS buffer interface at different local, but fixed bulk concentration.

Two FUS LC stock solutions were prepared at different concentrations, namely 75 µM and 37.5 µM so that 200 µL of the former or 400 µL of the latter were injected in the PBS buffer to reach a final FUS LC concentration of 3 µM. The obtained SP and SFG data are shown in Figure S14a and S14b, respectively. In Figure S14a no major differences in SP are observed, and the final SP values are similar. Figure S14b shows the intensity of the amide I signal is different, but not its spectral shape suggesting that the protein conformation is similar. Taking into account the similar SP values (and thus the similar amount of the FUS LC protein adsorbed at the interface), we speculate that the difference in the amide I signal intensity (but not shape) could arise from the different protein backbone orientation. The indistinguishable carbonyl stretching mode responses for both experiments suggests that the protein side-chain conformation is also very similar.

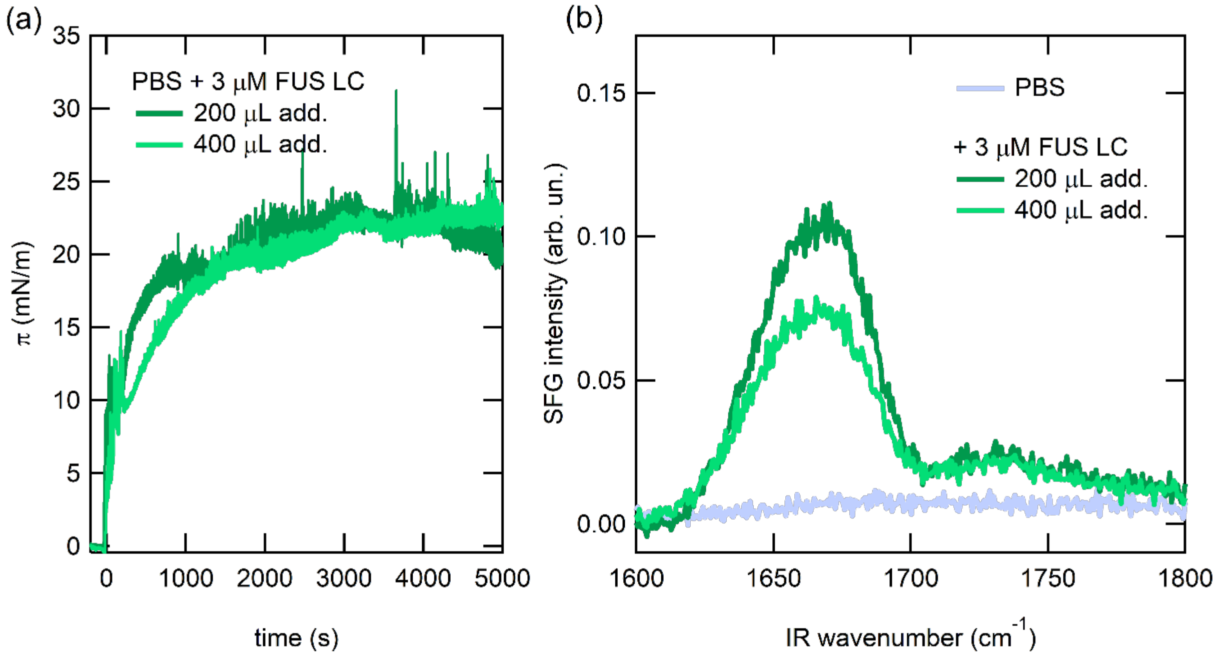

Figure S14. (a) Surface pressure curves for (dark green) 200 µL and (light green) 400 µL of FUS LC in the PBS buffer (final concentration in solution = 3 µM FUS LC); (b) SFG spectra in the amide I and carbonyl stretching region after equilibration (t~ 5000 s) for 3 µM FUS LC obtained by adding (dark green) 200 µL and (light green) 400 µL of the protein solution in the PBS buffer.

XIII. FTIR measurements of the FUS LC film.

We employed FTIR technique, which is sensitive to vibrational modes of molecules and their structure, but not to their orientation. We performed FTIR experiments, both from the on-liquid film, and the film transferred onto a solid substrate. To perform these measurements, we first determined the fraction of protein organized in the film vs. left in solution bulk. We found that, following the film formation, only ~5% of protein is left in bulk, while 95% is contained in the formed protein film (by checking the absorbance and recalculating for the protein concentration). The high partitioning of the protein into the film enables a straightforward interpretation of these (in principle, surface-insensitive) FTIR measurements, due to the limited contribution from the molecules in bulk. Because FTIR is usually performed for bulk liquids (or pellets) or solid samples in the ATR mode, we employed a special unit for the FTIR measurement of a protein film at the air/water interface in reflection geometry (see photo in Figure S15). To correct for fluctuating CO_2 and H_2O absorption in the background without applying nitrogen purging, which would otherwise perturb the liquid surface, polarization modulation (PM) using a photoelastic modulator (PEM) was essential to achieve the necessary signal-to-noise ratio in the infrared reflection absorption measurements (IRRAS).[11,12] This experimental design thus allowed for the *in situ* FTIR measurement of an on-liquid protein film.

As shown below (see Figure S16), the resulting PM-IRRAS amide I spectrum (dark blue line) is fully consistent with the SFG results (violet line), as shown below. In addition to PM-IRRAS data at the air/water interface, we also measured FTIR of the film on a solid substrate (blue line). For supported-film measurements, we used the same sample preparation as for the AFM measurements of the FUS LC film on a silicon wafer (shown in Figure 1 of the main text). However, due to the presence of water absorption bands, the amide band was obscured by the OH bending of water. Therefore, we deposited the film onto conventional CaF_2 windows, which allowed us to detect protein bands with standard transmission FTIR (see Figure S16).

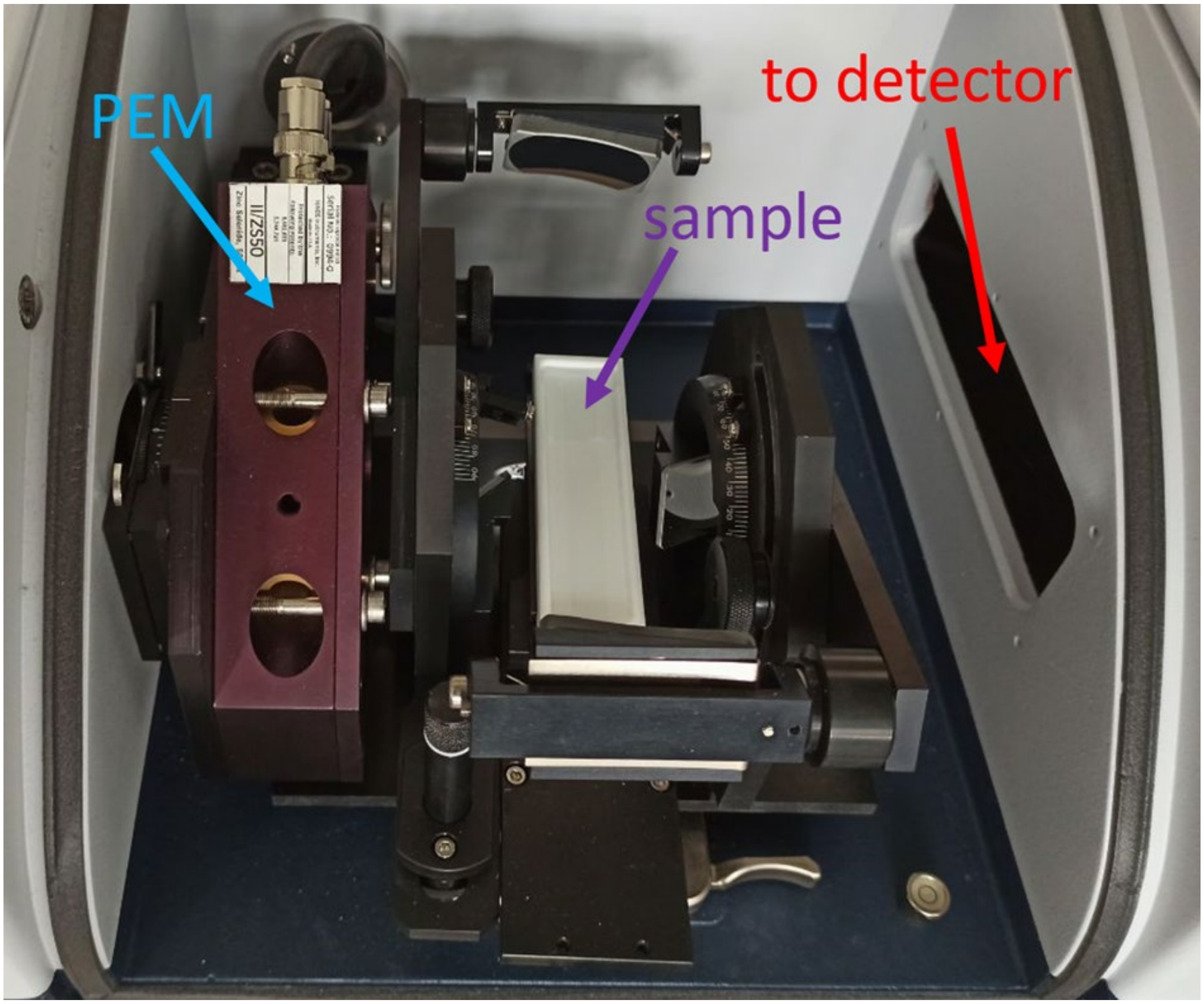

Figure S15. Unit designed for performing FTIR measurements of the air/liquid surface in reflection geometry.

Importantly, these new FTIR data show that the vibrational amide I response of the fibrillar film on a solid substrate is very similar to those of the film on buffer solution, both of which are highly similar to our original SFG data. Specifically, we find that the PM-IRRAS spectra from the on-liquid film and that deposited onto the solid substrate both show amide I and amide II bands centered at similar frequencies, confirming that fibrillar species are present already on the liquid interface. Center frequencies of amide I and amide II bands are ~1655 cm^{-1} and ~1540 cm^{-1}, respectively. The amide I band is broad, with several possible contributions: ~1620 cm^{-1} (β-turn or -sheet), ~1640 cm^{-1} and ~1655 cm^{-1} (random coil and α-helix), ~1685 cm^{-1} (β-turn or -sheet).[7,8] However, we find no dominant contribution at ~1620 cm^{-1} in contrast to FTIR (and also SFG) spectra reported for conventional amyloid fibrils.[5,13,14]

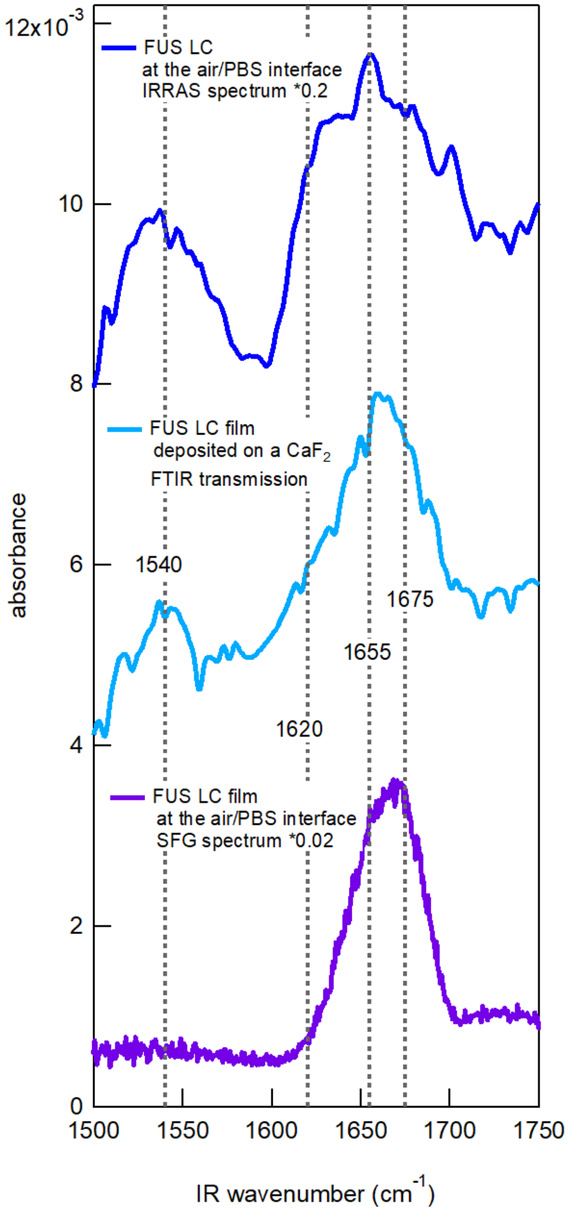

Figure S16. From top to bottom: (dark blue) FTIR spectrum of FUS LC film at the air/PBS buffer interface measured in reflection geometry with PM-IRRAS (3 μM FUS LC); (blue) Spectrum for the FUS LC film formed at the air/PBS buffer interface, deposited on the CaF_2 substrate, and measured in transmission mode with conventional FTIR; (violet) SFG spectrum of the FUS LC FUS LC film at the air/PBS buffer interface.

XIV. SFG signal in amide II spectral region.

In the study on human islet amyloid polypeptide (hIAPP), *Tan et al.* have revealed a correlation between the appearance of amide I (in fact, at ~1624 cm^{-1}) and amide II SFG bands, reporting on oligomer/fibril peptide organization.[5] The center frequency of the amide I peak for FUS LC is more blue-shifted compared to that observed for hIAPP. Despite that, hypothesizing that the amide II signal can also potentially be used for FUS LC as a reporter of fibrillar structures, we also investigated the amide II SFG spectra for FUS LC. As shown in Figure S17, we observe a very weak amide II (almost insignificant compared to amide I) signal, but its signal is too low for reliable analysis. Note that, due to interference between the different signals, the very weak amide II peak first appears as a slightly negative feature around 1580 cm^{-1}, before developing into a positive peak around 1560 cm^{-1}. Figure S17 shows three different sets of experiments recording the SFG spectra for the FUS LC film formed at the air/PBS buffer interface, with a 1.5 μM FUS LC initial concentration in solution bulk upon injection. By performing time-dependent measurements, we find that the amide II response develops and remains in the spectrum over time, which excludes that the amide II response is caused by intermediate states.

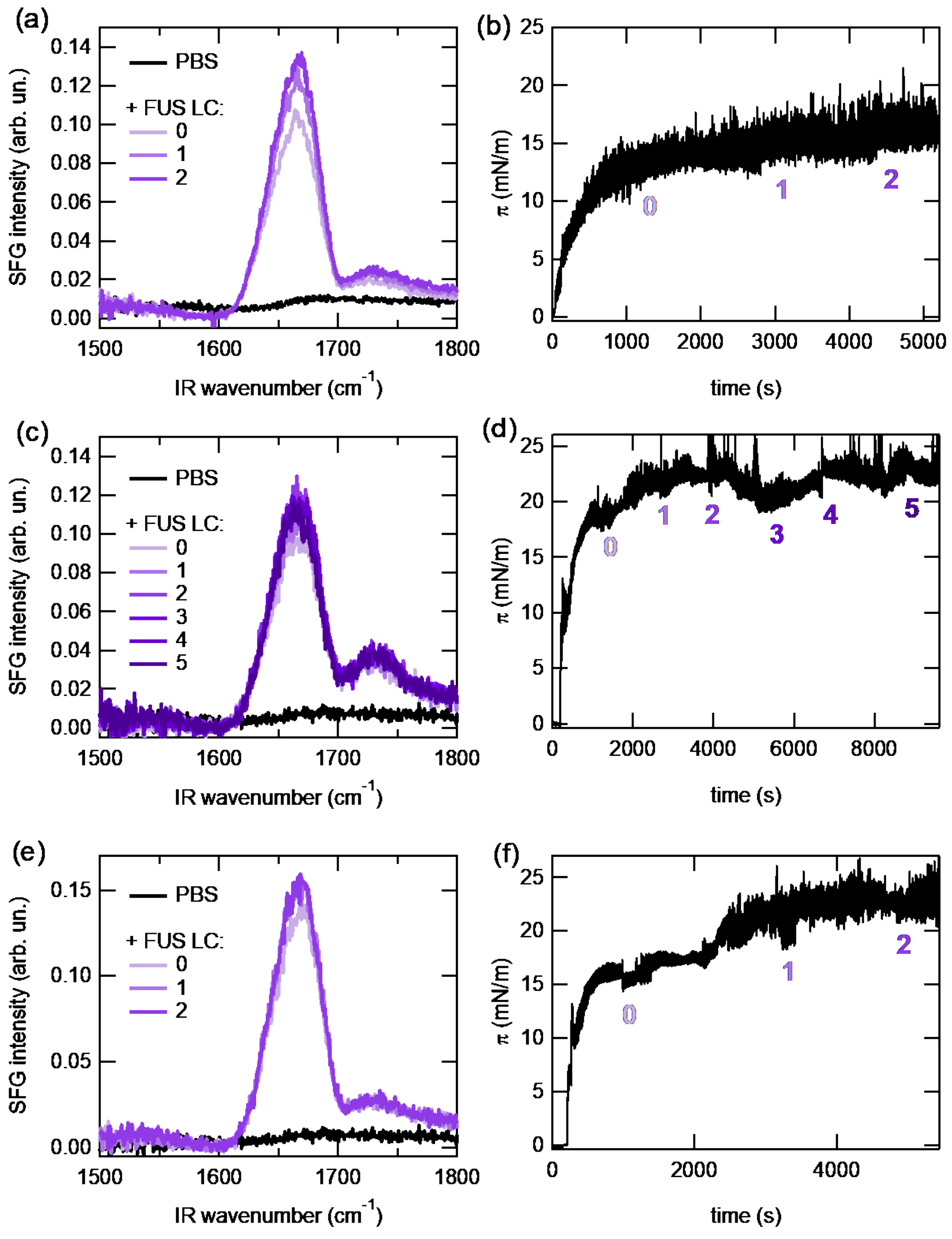

Figure S17. Three repetitions: (a), (c),(e) time-dependent SFG spectra in the amide II, amide I, and carbonyl regions for FUS LC film formed at the air/PBS buffer interface (initial bulk FUS LC concentration after the addition to the subphase is 1.5 μM). The spectra show that the amide I signal is reproducible in its details,

while the amide II response is very weak. (b), (d), (f) Corresponding surface pressure graphs. Numbers in (a), (c), € correspond to the markings in (b), (d), (f) to indicate when SFG spectra were acquired.

XV. FUS LC interaction with PC and TAP membranes investigated by SFG and SP.

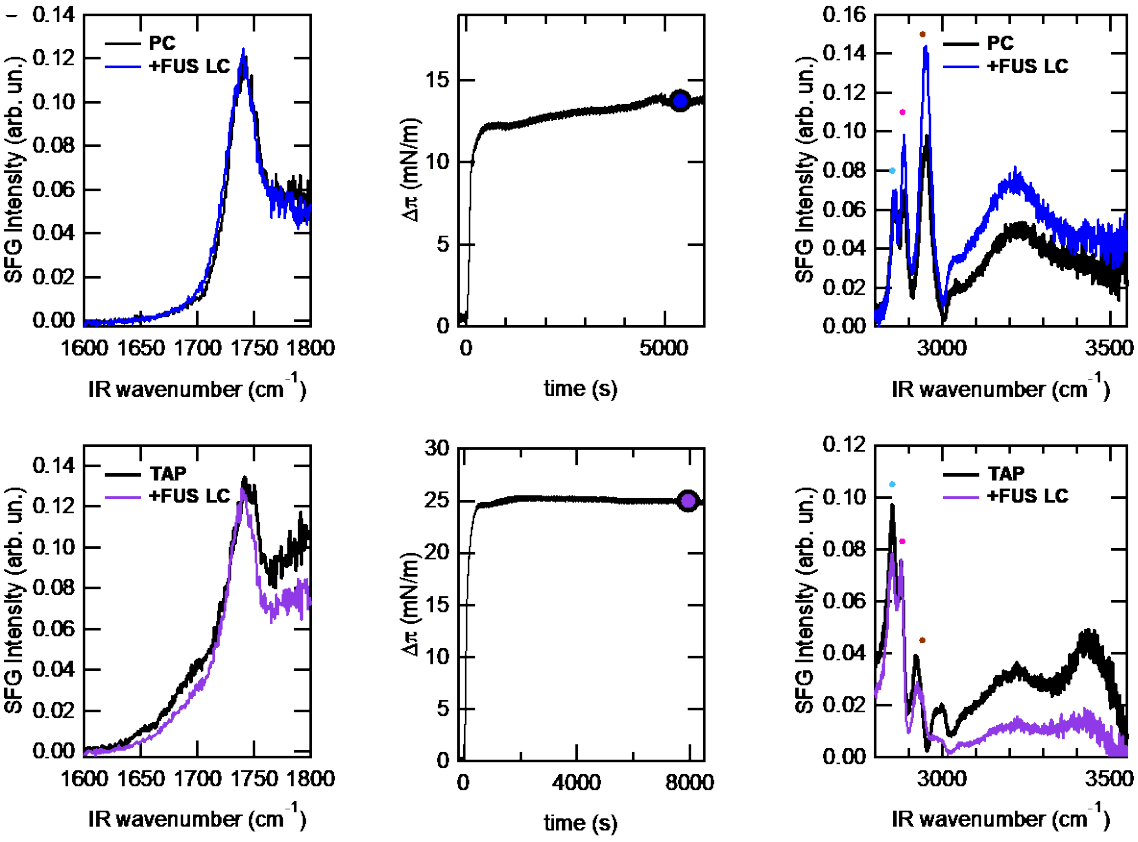

Figure S18. SFG spectra acquired in the amide I and carbonyl stretching region (a) for a DOPC monolayer (black) before and (blue) after FUS LC addition, (b) for a DOTAP monolayer (black) before and (purple) after FUS LC addition into PBS. All monolayers are prepared at surface pressure π~20 mN/m. Dependence of the surface pressure change Δπ on time after the addition of FUS LC in the PBS buffer subphase with the (c) DOPC and (d) DOTAP monolayer spread at the air/PBS solution interface. In (c) and (d), a circle indicates a time point at which the SFG spectrum was recorded. SFG spectra acquired in the -CH and -OH stretching region (e) for a DOPC monolayer (black) before and (blue) after FUS LC addition, (f) for a DOTAP monolayer (black) before and (purple) after FUS LC addition. In (e) and (f), the colored dots indicate the spectral peak assignment: (cyan) symmetric CH_2 stretching, (magenta) symmetric CH_3 stretching, and (brown) symmetric CH_3 stretching (Fermi resonance).

XVI. Homodyne SFG spectra in the CH-/OH-stretching region for 1.5 µM FUS LC at the air/PBS buffer interface.

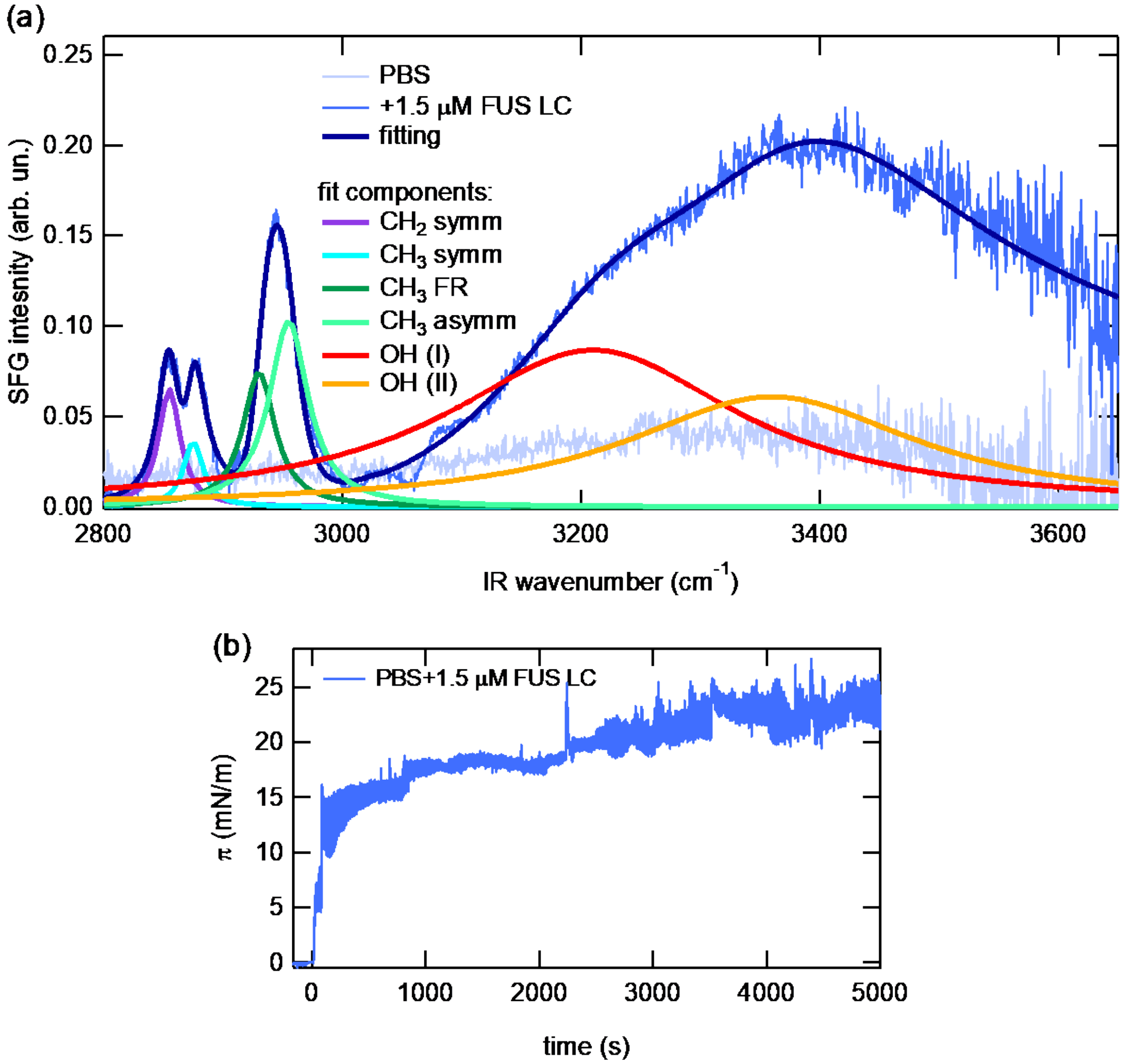

Figure S19. a) Homodyne SFG spectra acquired in the CH- and OH-stretching region for 1.5 µM FUS LC in PBS buffer (light) before and (dark) after the protein addition. The solid dark line presents the fitting curve. Resonant contributions (see Table S2) are presented as follows: (purple) CH_2 symm, (cyan) CH_3 symm, (green) CH_3 FR, (light green) CH_3 asymm, (red) and (orange) OH stretch contributions. For simplicity, the aromatic CH peak centered at ~3025 cm^{-1} is not presented, due to low contribution. (b) The corresponding surface pressure curve.

After FUS LC is added in the PBS buffer, a substantial increase in the OH-stretching signal intensity is observed (Figure S19a). Likely, the observed increase is due to an increased ordering of water molecules at the air/PBS buffer interface due to ordered proteins present at the interface.

The fitting of the complete CH- and OH-stretching region was performed. Each resonant contribution was fitted with a Lorentzian. Parameters obtained from the fitting are presented in Table S2. The signs selected for the Im($\chi^{(2)}$) of the contributions are as follows (according to HD-SFG, Figure 3b in the main text): CH_2(symm)<0, CH_3(symm)<0, CH_3(FR)<0, CH_3(antisymm)>0, CH(aromatic)>0, OH(3200 cm^{-1})>0, OH(3400 cm^{-1})>0.

Table S2. Results of the fitting for SFG spectra presented in Figure S19a.

	PBS+1.5 µM FUS LC
A_{NR}	0.2
ϕ_{NR}	3.5
A(CH_2 symm)	2.8
ω(CH_2 symm)	2855
2Γ(CH_2 symm)	22
A(CH_3 symm)	1.9
ω(CH_3 symm)	2875
2Γ(CH_3 symm)	20
A(CH_3 FR)	4.5
ω(CH_3 FR)	2930
2Γ(CH_3 FR)	33
A(CH_3 asymm)	-6.4
ω(CH_3 asymm)	2955
2Γ(CH_3 asymm)	40
A(aromatic)	-0.04
ω(aromatic)	3025
2Γ(aromatic)	20
A(OH I)	-44
ω(OH I)	3210
2Γ(OH I)	299
A(OH II)	-37
ω(OH II)	3360
2Γ(OH II)	300

Based on the HD-SFG data, we propose that the presence of FUS LC (which adsorbs, orders, and folds at the air/PBS buffer interface) aligns hydrogen-bonded interfacial water (with hydrogens pointing towards the air phase). We note that when we compare the homodyne SFG result (Figure S19a) of a separately performed experiment with the heterodyne SFG result (Figure 3b), it is evident that in homodyne SFG data the difference in the OH-stretching signal intensity (with *vs.* without FUS LC) is bigger than that in heterodyne SFG data. This can, for the most part, be explained by the homodyne SFG signal being proportional to the square of the second-order

susceptibility. Figure 3b shows a two-fold increase in the heterodyne signal strength for water in the presence of FUS - this leads to a four-fold increase in SFG intensity, as observed in Figure S19a. Secondly, the experiments were performed under slightly different experimental conditions. For the homodyne SFG, during the entire experiment, the SP needle was inserted into the trough (stainless steel trough, D=4.5 cm), and the trough was rotated. For the heterodyne SFG, during the experiment, there was no SP needle in the trough (pure Teflon trough, D=2.9 cm), and the trough was not rotated during the measurement to maintain the phase stability.

XVII. Control experiments: HD-SFG of FUS LC in CAPS pH 11, and HD-SFG of 0.8 mM CAPS in PBS.

We measured HD-SFG spectra for the pristine CAPS buffer (20 mM pH 11) and that in the presence of FUS LC (1.5 μM concentration in bulk). The corresponding spectra for the imaginary part of the complex second-order nonlinear response $\chi_{eff}^{(2)}$ (which contains information on vibrational resonances and is free from the non-resonant background) are presented in Figure S20.

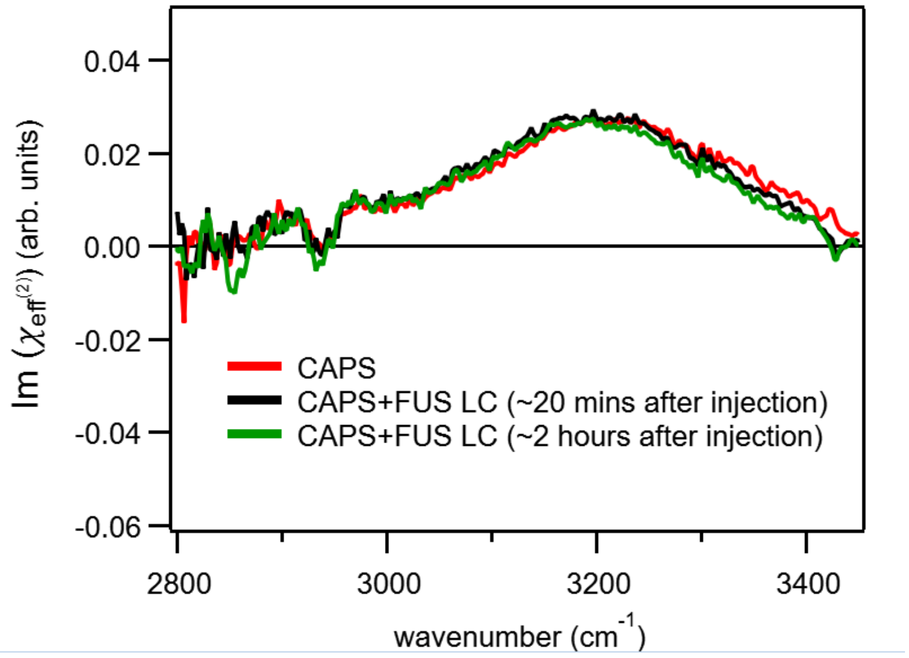

Figure S20. Imaginary part of the second-order nonlinear susceptibility for (red) the pristine CAPS buffer (20 mM pH 11) and that after addition of FUS LC into the subphase and measured ~20 mins (black) and ~2 hours (green) since the protein injection.

The spectrum of the pristine CAPS pH 11 buffer shows a positive signal in the OH-stretch region (reporting, on average, H-up oriented hydrogen-bonded OH moieties), and a minor contribution from CH (consistent with a minor, slow, but steady increase in SP and amide I SFG signal, see Figures 1 and 3a in the main text, respectively). The H-up orientation of water can be understood by noting that, since the pH of the CAPS solution 0.6 pH units above the pK_a (pH 11, pK_a=10.4), ~ half of the CAPS molecules will be deprotonated and negatively charged. The interfacial water molecules will interact with the negatively charged sulfonate group resulting in the H-up orientation of interfacial water molecules. A substantial red shift of the OH stretch down to 3200 cm^{-1} can arise from the $\chi^{(3)}$ (bulk) contribution in addition to interfacial $\chi^{(2)}$ response. We note that while the orientation of the water molecules is similarly H-up for pure CAPS buffer, this spectrum in Figure S20 (i) is not relevant for the SFG spectra reported in the manuscript and (ii) differs significantly from the positive peak at 3330 cm^{-1} for water interacting with FUS (Figure 3b in the main text). That is, FUS and CAPS have different interactions with water molecules, as expected. After adding FUS LC to CAPS pH 11 buffer solution, we do not observe any significant change in the OH signal and only minor changes in the CH signal (consistent with the small signal detected in the amide I region). This result is expected since FUS LC molecules predominantly reside in bulk. We note that the above experiments, while insightful, are not particularly relevant for the interpretation of our results.

The most relevant are HD-SFG experiments having a smaller amount of CAPS within the PBS subphase as is done when FUS LC film is formed in our SFG experiments. In the presence of protein, the CAPS concentration is equal to 0.8 mM in PBS (since we inject 0.2 mL of 20 mM CAPS into 4.8 mL PBS). In this case, the interfacial water signal is identical to that in the pure PBS since there is very little CAPS in the solution to adsorb to the surface (see Figure S20). This is entirely consistent with Figure S5 showing almost no change in SP with 0.8 mM CAPS in a PBS subphase. We also recorded HD-SFG spectrum with the sample trough rotation switched on to exclude possible effect of the laser pushing molecules out of focus area. The spectra of pure PBS and 0.8 mM CAPS + PBS (with and without rotation) are identical. Thus we confirm that the signal presented in Figure 3b is indeed the effect of FUS LC (and not arising from CAPS surface propensity).

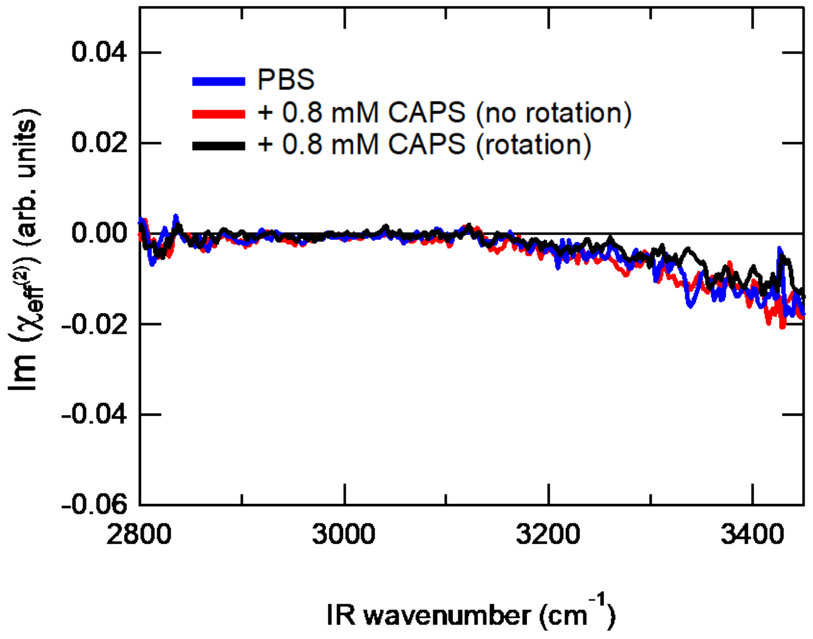

Figure S21. Imaginary part of the second-order nonlinear susceptibility for (blue) the pristine PBS buffer, (red) and (black) that after addition of 20 mM CAPS pH 11 buffer solution into the PBS subphase with the following switching of the trough rotation on, for the black curve.

XVIII. $\chi^{(3)}$ analysis of the SFG spectra in the OH-stretching region for 1.5 μM FUS LC at the air/PBS buffer interface.

$\chi_{eff}^{(2)}$ response measured with the heterodyne SFG spectroscopy for FUS LC at the air/PBS buffer interface and presented in Figure 3b of the main text was modelled according to the following formula:[15]

$$\chi_{eff}^{(2)}(\omega) = A_{NR}e^{i\varphi_{NR}} + \chi_{CH}^{(2)}(\omega) + \chi_{OH}^{(2)}(\omega) + \chi_{OH}^{(3)}(\omega) \cdot \Phi(\sigma, c) \cdot \frac{\kappa(c)}{\kappa(c) - i \cdot \Delta k_z},$$

where A_{NR} and φ_{NR} are amplitude and phase of the non-resonant contribution; $\chi_{CH}^{(2)}$ and $\chi_{OH}^{(2)}(\omega)$ are second-order nonlinear resonant contribution of CH- and OH-stretching vibrational modes, respectively; $\chi_{OH}^{(3)}$ is the third-order nonlinear susceptibility; $\Phi(\sigma, c)$ is the surface potential where σ is the surface charge density and c is the salt concentration; κ is the inverse Debye length, Δk_z is the mismatch of the wave-vectors along the z axis:[15,16]

$$\Phi(\sigma, c) = \frac{2k_BT}{e} \cdot sinh^{-1}\left(\frac{\sigma}{\sqrt{8000k_BTN_Ac\varepsilon_0\varepsilon_r}}\right),$$

$$\kappa(c) = \sqrt{\frac{2000e^2N_Ac}{\varepsilon_0\varepsilon_rk_BT}},$$

$$\Delta k_z = k_{SFG,z} + k_{VIS,z} + k_{IR,z},$$

$$k_{i,z} = \frac{2\pi}{\lambda_i}\sqrt{n_i^2 - \sin^2(\theta_i)},$$

where i corresponds to the SFG, Vis or IR beam, λ_i is the wavelength and θ_i is the incident angle of the i-th beam.

For our case the considered salt concentrations are as follows: $c_1 = 0.137\ \frac{mol}{L}$ (only NaCl from the PBS buffer was taken into account here), $c_2 = 0.313\ \frac{mol}{L}, c_3 = 0.665\ \frac{mol}{L}$. Further, we construct the differences between the imaginary part (Im) of the $\chi_{eff}^{(2)}(\omega)$ response measured at different salt concentrations, c_s and c_n:

$$Im\left(\chi_{eff}^{(2)}(c_s)\right) - Im\left(\chi_{eff}^{(2)}(c_n)\right) = Im\left(\chi_{OH}^{(3)}(\omega) \cdot \Phi(\sigma, c_s) \cdot \frac{\kappa(c_s)}{\kappa(c_s) - i \cdot \Delta k_z}\right) - Im\left(\chi_{OH}^{(3)}(\omega) \cdot \Phi(\sigma, c_n) \cdot \frac{\kappa(c_n)}{\kappa(c_n) - i \cdot \Delta k_z}\right)$$

For simplicity, we denote the real and imaginary parts of $\chi_{OH}^{(3)}(\omega)$ as follows

$$\chi_{OH}^{(3)}(\omega) = Re\left(\chi_{OH}^{(3)}(\omega)\right) + i \cdot Im\left(\chi_{OH}^{(3)}(\omega)\right) = a(\omega) + i \cdot b(\omega)$$

and write the equations for the salt concentrations c_1, c_2, and c_3:

$$\begin{cases} Im\left(\chi_{eff}^{(2)}(c_1)\right) - Im\left(\chi_{eff}^{(2)}(c_2)\right) = \Phi(\sigma, c_1) \cdot \kappa(c_1) \cdot \dfrac{a(\omega) \cdot \Delta k_z + b \cdot \kappa(c_1)}{\left(\kappa(c_1)\right)^2 + (\Delta k_z)^2} - \Phi(\sigma, c_2) \cdot \kappa(c_2) \cdot \dfrac{a(\omega) \cdot \Delta k_z + b \cdot \kappa(c_2)}{\left(\kappa(c_2)\right)^2 + (\Delta k_z)^2} \\ Im\left(\chi_{eff}^{(2)}(c_2)\right) - Im\left(\chi_{eff}^{(2)}(c_3)\right) = \Phi(\sigma, c_2) \cdot \kappa(c_2) \cdot \dfrac{a(\omega) \cdot \Delta k_z + b \cdot \kappa(c_2)}{\left(\kappa(c_2)\right)^2 + (\Delta k_z)^2} - \Phi(\sigma, c_3) \cdot \kappa(c_3) \cdot \dfrac{a(\omega) \cdot \Delta k_z + b \cdot \kappa(c_3)}{\left(\kappa(c_3)\right)^2 + (\Delta k_z)^2} \end{cases}$$

The system above is a system of linear equations with respect to $a(\omega)$ and $b(\omega)$. The left side of each equation represents acquired experimental data.

FUS LC is usually treated as a neutrally charged protein. However, it is actually minorly negatively charged at pH=7.4 (PBS buffer) as contains two charged amino acids. According to various protein calculators available online, at a given pH, FUS LC molecule possesses a negative charge in the range from $(q_0)_a = 2$ to $(q_0)_b = 3$ (expressed in elementary electron charge units). Assuming that we are in the limiting case where all FUS LC molecules added in the PBS subphase are adsorbed at the air/buffer interface (overestimation), we calculate the maximal surface charge density σ possible:

$$\sigma = \frac{V \cdot N_A \cdot c_m^{FUS} \cdot q_0 \cdot e}{S}$$

where V is the volume of PBS in the trough ($V = 5$ mL), N_A is Avogadro number, c_m^{FUS} is the molar concentration of FUS LC in the subphase (here, $c_m^{FUS} = 1.5\ \mu M$), S is the surface area of the used trough (diameter D=2.9 cm). The calculations were further performed using the value $q_0 = (q_0)_a = 2$. The corresponding calculated surface charge density is $\sigma_a \simeq -2.06\ \frac{C}{m^2}$ (whereas $\sigma_b \simeq -3.09\frac{C}{m^2}$ for $q_0 = (q_0)_b = 3$).

Solving the above presented system of linear equations with relation to a and b, one obtains the following expressions:

$$a \simeq 1.74 \cdot 10^{22} \cdot \left(Im\left(\chi_{eff}^{(2)}(c_1)\right) - Im\left(\chi_{eff}^{(2)}(c_2)\right)\right) - 4.19 \cdot 10^{17} \cdot \left(Im\left(\chi_{eff}^{(2)}(c_2)\right) - Im\left(\chi_{eff}^{(2)}(c_3)\right)\right)$$

$$b \simeq -1.46 \cdot 10^{29} \cdot \left(Im\left(\chi_{eff}^{(2)}(c_1)\right) - Im\left(\chi_{eff}^{(2)}(c_2)\right)\right) + 5.12 \cdot 10^{24} \cdot \left(Im\left(\chi_{eff}^{(2)}(c_2)\right) - Im\left(\chi_{eff}^{(2)}(c_3)\right)\right)$$

Inserting experimental data into these expressions, we get $a(\omega)$ and $b(\omega)$, i.e the real and imaginary parts of $\chi_{OH}^{(3)}(\omega)$. Further, we plot the imaginary part of the entire $\chi^{(3)}$ contribution, i.e. $Im\left(\chi_{OH}^{(3)}(\omega) \cdot \Phi(\sigma, c) \cdot \frac{\kappa(c)}{\kappa(c) - i \cdot \Delta k_z}\right)$ (at highest salt concentration c_3 as presented in Figure S22, red curve) versus frequency ω together with the experimental data, $Im\left(\chi_{eff}^{(2)}(\omega)\right)$.

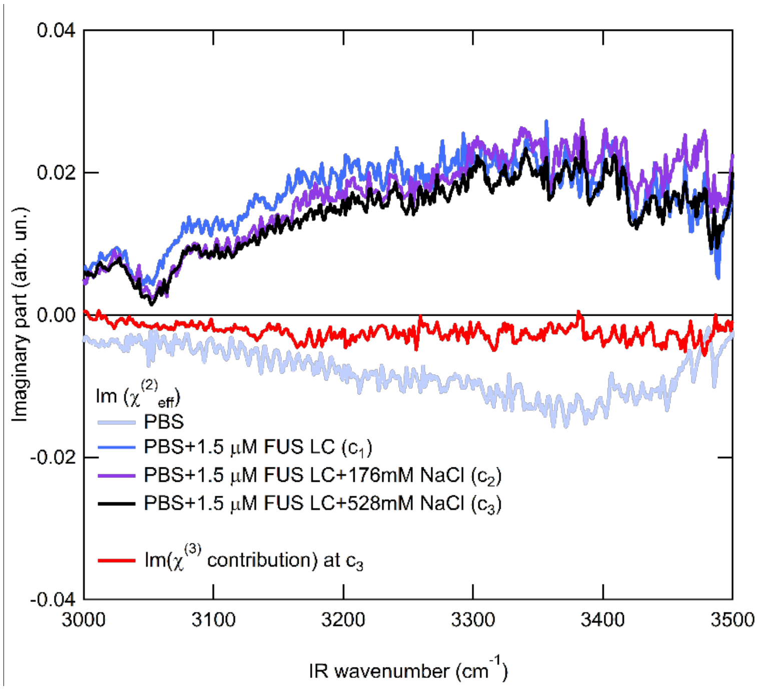

Figure S22. Imaginary part of heterodyne SFG data acquired in the OH-stretching region for (light blue) pure PBS, (blue) PBS in presence of 1.5 μM FUS LC (total salt concentration $c_1 = 137$ mM), (purple) PBS in presence of 1.5 μM FUS LC and additional 176 mM NaCl (total salt concentration $c_2 = 313$ mM), (black) PBS in presence of 1.5 μM FUS LC and additional 528 mM NaCl (total salt concentration $c_3 = 665$ mM). (Red) imaginary part of the $\chi^{(3)}$ contribution calculated for $c_3 = 665$ mM.

Figure S22 clearly shows that the main contribution in the measured $\chi_{eff}^{(2)}$ consists mainly of the $\chi^{(2)}$ term and not the $\chi^{(3)}$ term. A much larger $\chi^{(2)}$ contribution than the $\chi^{(3)}$ contribution indicates that the SFG signal probed in the hydrogen-bonded OH-stretching region originates from the molecular ordering of OH moieties in the topmost layers at the air/PBS buffer interface.

XIX. SP data for PBS→CAPS→PBS buffer exchange experiment.

SP measurements were performed with a height sensor (Keyence LK-G82 Lasersensor) to correct for possible evaporation from the liquid surface (and for changes of the surface height during buffer exchange experiment). As stated in the main text, the FUS film was first formed at the air/PBS buffer solution interface (we further recall this part of the experiment as “PBS”). Once the film formation equilibrated, as was determined by a stabilized SP, the PBS buffer subphase was exchanged to CAPS in the following way: without perturbing the formed film, the CAPS buffer solution was added into and an equal volume of the subphase was simultaneously removed from the trough. This cycle was repeated 5X, which showed a subphase pH (checked with a pH meter) consistent with that of the exchange buffer (pH 11 for CAPS). Once the PBS→CAPS buffer was exchanged, the film was again allowed to equilibrate. This experiment is referred to as “PBS →CAPS”. In experiments where the buffer was again echanged to PBS, “PBS→CAPS→PBS”, we performed the buffer exchange from CAPS back to PBS as described and the pH was verified. Note that during the buffer exchange, no additional protein was added into the subphase. See Figure S23 for the SP data obtained.

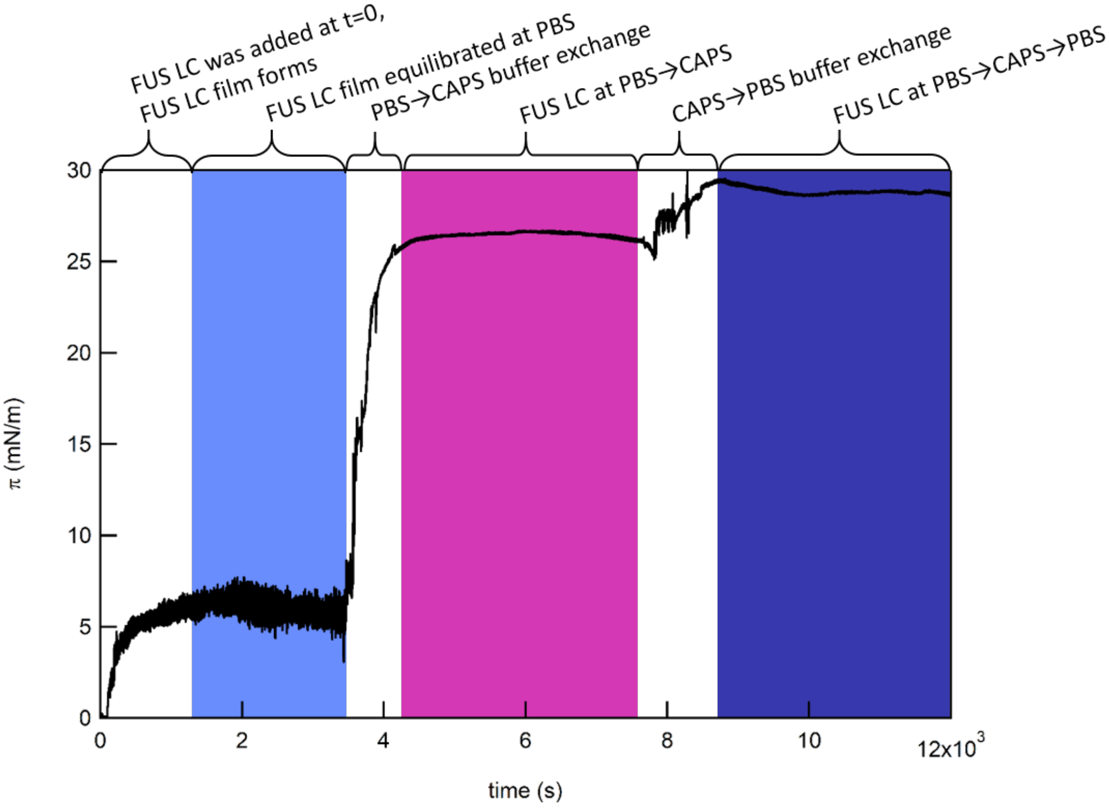

Figure S23. SP data for a PBS→CAPS→PBS buffer exchange experiment includes the stages as follows: FUS LC film forms after addition of FUS LC in the PBS subphase at a final concentration of 1.5 μM at t=0;

FUS LC film formed at the air/buffer solution interface and equilibrated as determined by stabilized SP (denoted as a “PBS” part of the experiment, blue); PBS→CAPS buffer exchange is performed; FUS LC equilibrated at the air/CAPS interface (denoted as a “PBS→CAPS” part of the experiment, purple); CAPS→PBS buffer exchange is performed; FUS LC organized at the PBS interface (denoted as a “PBS→CAPS→PBS” part of the experiment, dark blue).

XX. Analysis of SP fluctuations.

In addition to the SP average values, we quantified the amplitude of SP fluctuations detected during each stage of buffer exchange experiments, “PBS”, “PBS→CAPS”, “PBS→CAPS→PBS” (see explanation of notations in Figure S23). Zooming-in to the raw surface pressure data presented in Figure S23 reveals a periodicity in signal, which is shown in Figures S24a, S25a, and S26a for “PBS”, “PBS→CAPS”, and “PBS→CAPS→PBS”, respectively. Note that we shifted the SP signal along the vertical axis by the average SP value $\bar{\pi}$. Results of the Fourier transform analysis performed on the autocorrelated surface pressure measurements (ACF, i.e. $\langle(\pi(t)-\bar{\pi})\cdot(\pi(t+\tau)-\bar{\pi})\rangle$ where τ is a lag time) is presented in Figures S24c, S25c, and S26c. Taking the FFT of ACF reveals the dominant frequency centered at 0.08 Hz which corresponds to a period of T~12.5 s. As these expeiments are performed with a rotation trough, this periodicity corresponds to the rotational motion of ~5 rpm of the trough. The reason for such periodic SP curve fluctuation is solely technical: upon the trough rotation, the surface of the liquid in the trough is not flat, but has some curvature due to the centrifugal force. At the same time, if the trough is positioned not at the exact center of the rotation stage (whose center determines the rotation axis), the SP needle inserted into the trough then makes a circle at the surface with the center displaced with repect to the center of the trough. Since the liquid surface has some curvature, the depth of the needle insertion is a periodic function of time with a period equal to the time needed for one rotation of the trough, which accounts for the presence of the periodic signal we observe in SP curve. Since periodicity originates due to the technical reason, we filter it out using an infinite impulse response (IIR) notch filter with the center frequency corresponding to 0.08 Hz. The clean, filtered SP signal is presented in Figures S24d, S25d, and S26d, respectively. For the analysis and estimation of the SP fluctuation amplitude, three non-overlapping regions at each stage of the experiment were chosen so that the number of points in each region was N=2000 which corresponded to a 500s time interval. For each region, the signal was filtered according to the procedure described above and the standard deviation was estimated. The mean of those standard deviation values and the error were calculated (taking Student coefficient for α=0.9), and the data were presented in Figure S27.

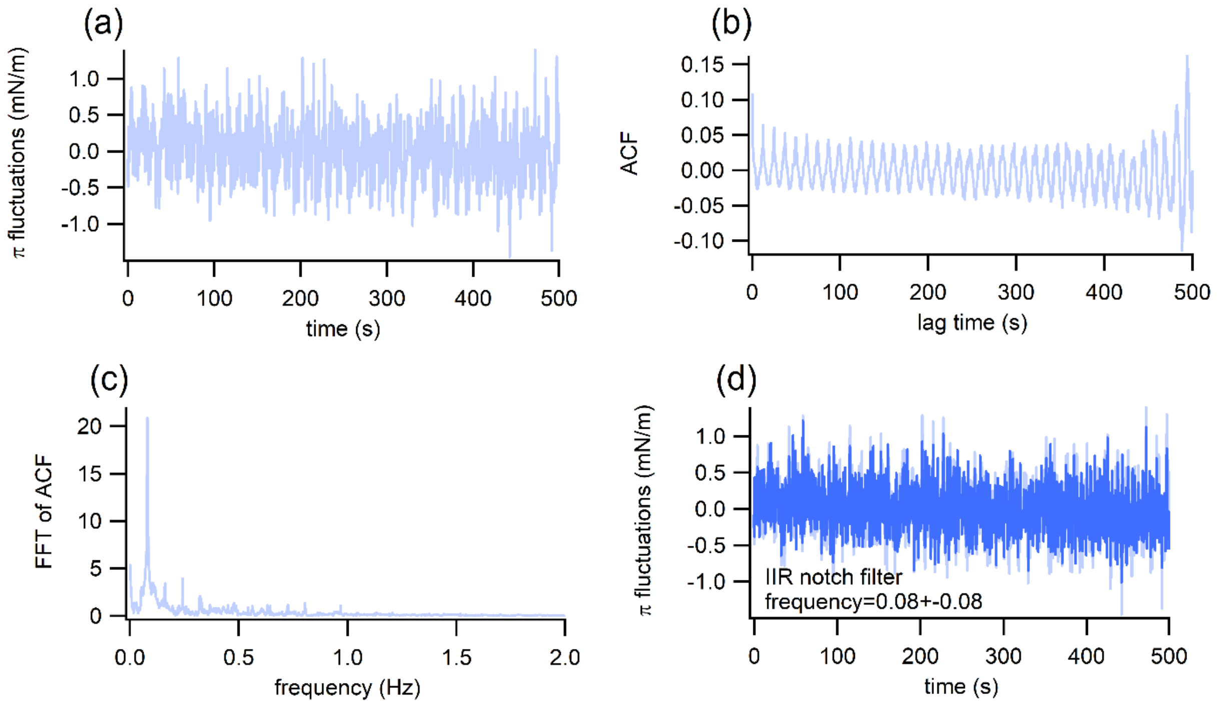

Figure S24. SP fluctuation analysis for the "PBS" experimental stage presented in Figure S23: (a) π versus time for the 500s interval. The graph is shifted in vertical axis to have $\bar{\pi} = 0$ mN/m and in horizontal axis to have the SP curve region started at t=0. (b) ACF calculated for the data presented in (a). (c) FFT of ACF. (d) The original unfiltered SP signal (light blue) and the signal obtained after the 0.08 Hz frequency was filtered out using the IIR notch filter (blue).

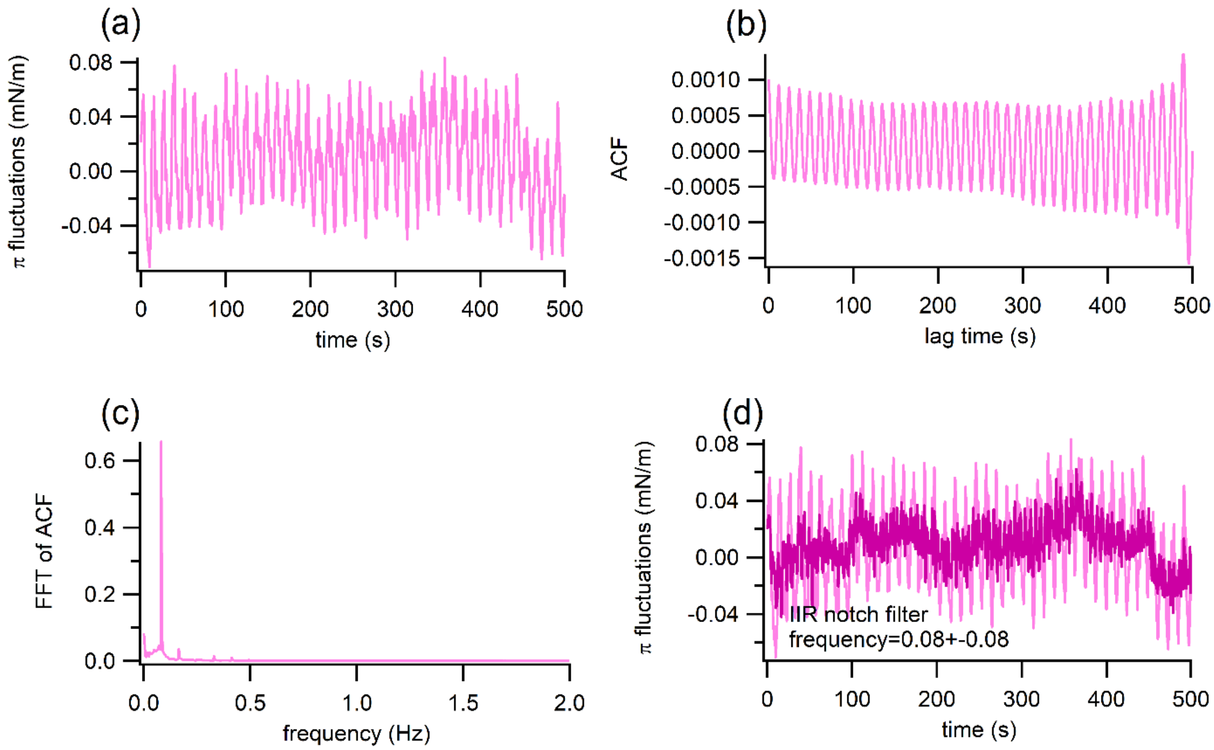

Figure S25. SP fluctuation analysis for the “PBS→CAPS” experimental stage presented in Figure S23: (a) π versus time for the 500s interval. The graph is shifted in vertical axis to have $\bar{\pi} = 0$ mN/m and in horizontal axis to have the SP curve region started at t=0. (b) ACF calculated for the data presented in (a). (c) FFT of ACF. (d) The original unfiltered SP signal (light purple) and the signal obtained after the 0.08 Hz frequency was filtered out using the IIR notch filter (purple).

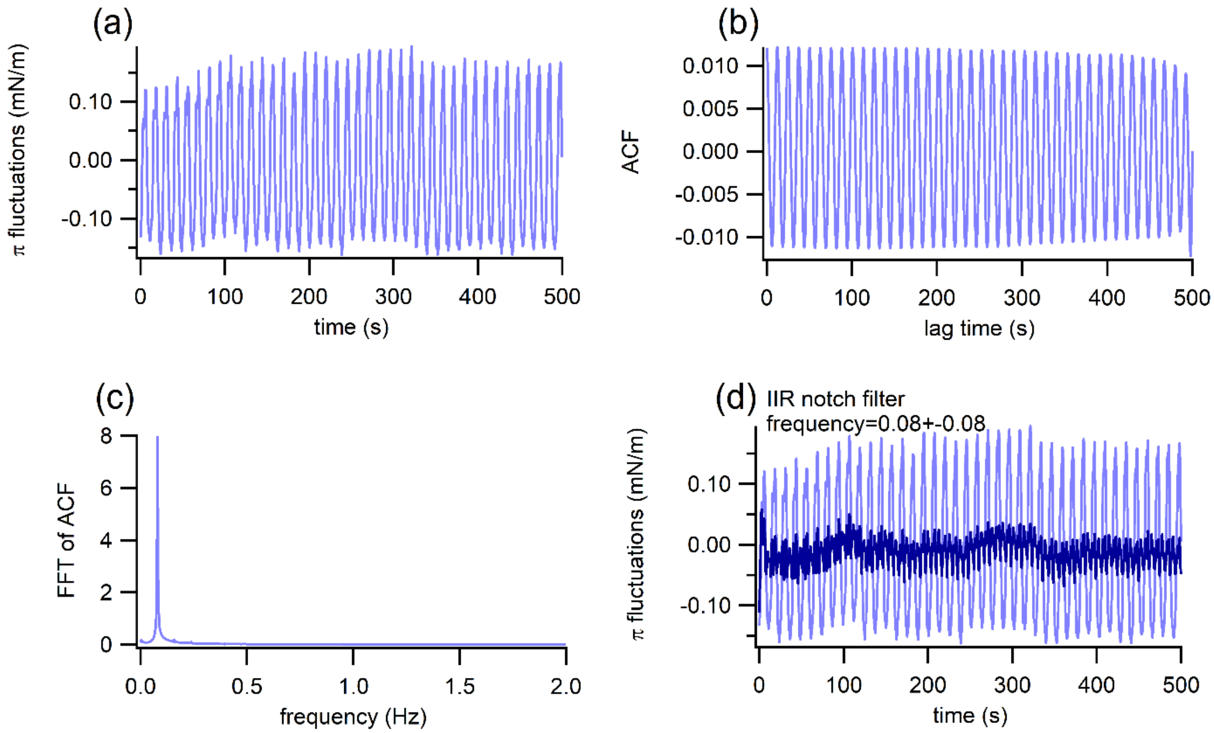

Figure S26. SP fluctuation analysis for the "PBS→CAPS→PBS" experimental stage presented in Figure S23: (a) π versus time for the 500s interval. The graph is shifted in vertical axis to have $\bar{\pi} = 0$ mN/m and in horizontal axis to have the SP curve region started at t=0. (b) ACF calculated for the data presented in (a). (c) FFT of ACF. (d) The original unfiltered SP signal (light blue) and the signal obtained after the 0.08 Hz frequency was filtered out using the IIR notch filter (dark blue).

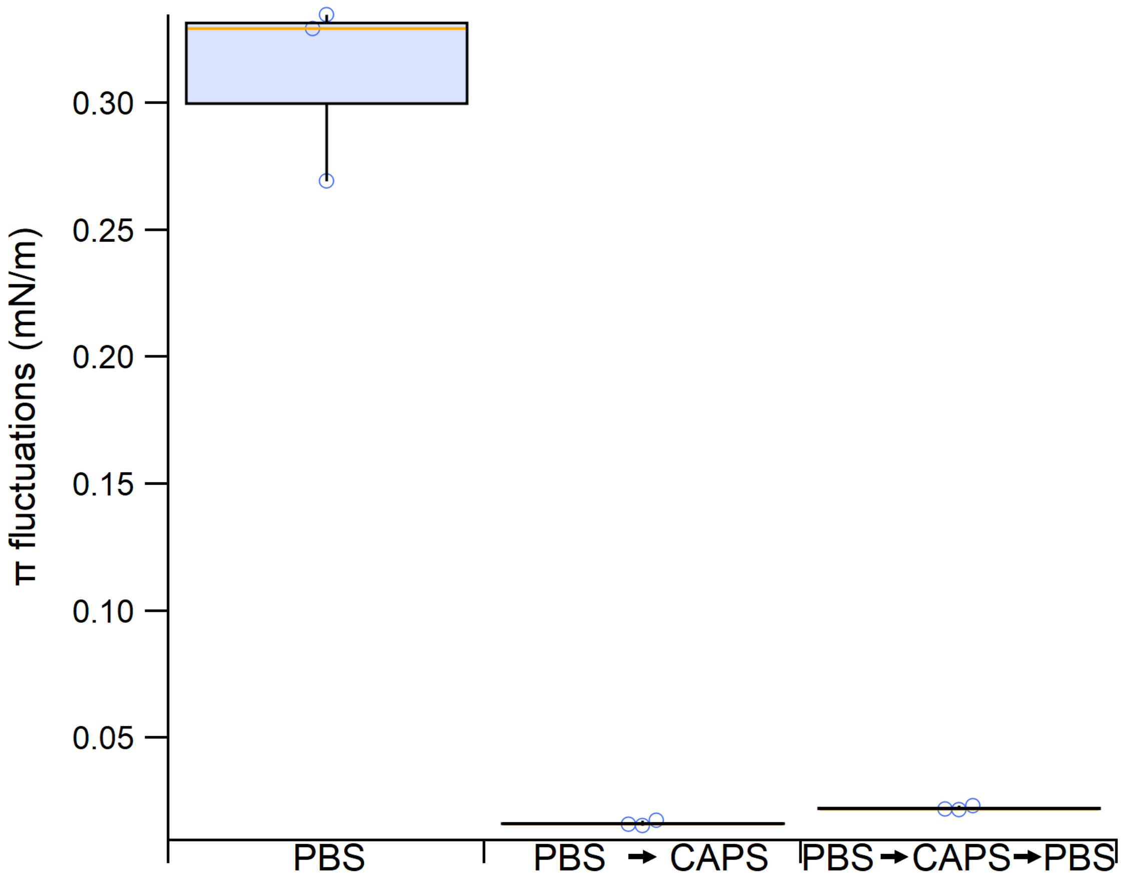

Figure S27. Fluctuations of surface pressure characterized by standard deviation from mean surface pressure for 1.5 μM FUS LC in PBS after film formation (PBS), after the PBS buffer was exchanged to CAPS pH 11 (PBS -> CAPS), and after the subsequent buffer exchange of CAPS back to PBS (PBS -> CAPS -> PBS). Flucations are analyzed from N=3 separate experiments, each having independent regions of each buffer condition. Individual measurements are represented by clear circles; whiskers are min/max of the data; box widths are the first and third quartiles of the data; orange lines are the medians.

XXI. Protein mobile fraction during buffer exchange.

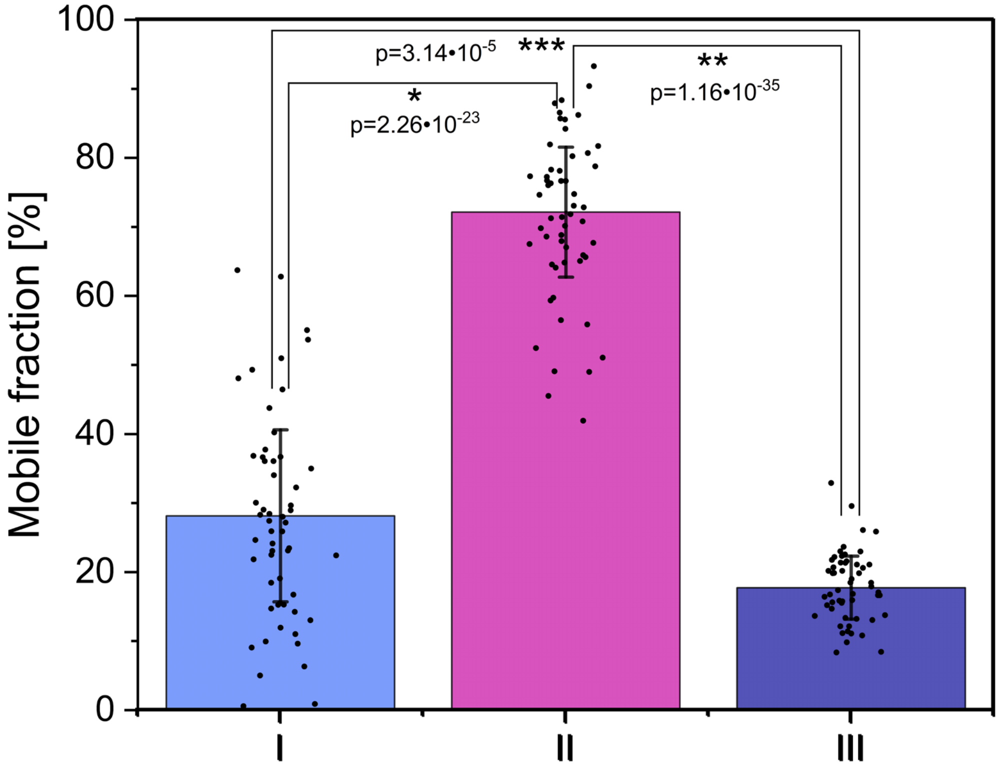

Figure S28. Bar graphs showing mobile fraction inferred from the FRAP data in Figure 4c in the main text. Mobile fraction data is extracted from N = 56 FRAP unique curves. Bars are mean, and the error bars are standard deviation. Asterisks show statistical significance, $P < 0.05$ using a Two-way ANOVA followed by a Student t-tests between different groups. I, II, III denote three experimental stages namely PBS, PBS→CAPS, PBS→CAPS→PBS, respectively. Individual data points are shown as dots.

XXII. FTIR experiments on FUS LC film upon buffer exchanges.

In complement to SFG, we present additional FTIR experiments which are insensitive to molecular orientation. For these measurements, we used the solid-supported films measurements to avoid the challenges of buffer exchange in the PM-IRRAS setup. Having established that film transfer to the CaF_2 windows did not fundamentally add or remove vibrations compared to the on-liquid film, this allowed us to make the buffer exchanges and examine the vibrational spectra of the film with FTIR thereafter (3 spectra, buffer exchange data, see Figure S29 below). Surprisingly, after the PBS→CAPS buffer exchange and film deposition, along with the peak centered at ~1670 cm^{-1}, we observe a distinct peak at ~1620 cm^{-1} (purple curve, Figure S29). To check whether this peak originates from the protein, we performed a control experiment: we “deposit” and measure FTIR of the subphase after the PBS→CAPS buffer exchange but without FUS LC present (light magenta curve, Figure S29). The results show that the response at ~1620 cm^{-1} is not from the protein, but from the CAPS molecule present in the buffer. In fact, this peak is also observed for the film deposited on a substrate after the PBS→CAPS→PBS buffer exchange (dark blue curve). The experiments thus show that the FTIR spectrum at ~1620 cm^{-1} is “contaminated” by this vibrational mode of the CAPS molecule, which complicates the interpretation of spectra and addressing the question if there is any protein contribution present at this frequency. The FTIR spectrum of CAPS molecule (see https://spectrabase.com/spectrum/AQFVQINGKyn) confirms that this resonance originates from CAPS. However, at the same time, CAPS does not have any significant response at this frequency in Raman (see https://spectrabase.com/spectrum/9ruifMdyFQF), so that in the SFG measurements (requiring both IR- and Raman-activity), this CAPS mode does not appear.

Summarizing our findings, the FTIR data thus support the SFG data, in that neither of the techniques contains an indication of the distinct amide I response at ~1620 cm^{-1}, for the original film formed at the PBS subphase. For the film after the PBS→CAPS→PBS buffer exchange, a weak SFG signal is detected at ~1620 cm^{-1}. Using FTIR, we tried to clarify the origin of this contribution. We have, however, revealed that CAPS contributes to FTIR spectrum, and this fact complicates the interpretation of CAPS vs. possible protein contribution at that frequency. At the same time, based on our data along with the available Raman spectrum, we conclude that, while being FTIR-active, CAPS cannot contribute to the SFG response due to the lack of Raman active modes in the amide I region. The combination of these experiments allows us to conclude that the SFG response at 1620 cm^{-1} evident after the PBS→CAPS→PBS exchange indeed originates from FUS LC. The amide SFG spectra of the original PBS-film and the one formed after buffer exchange with CAPS and returning to PBS show that the film is structurally different at the molecular scale, with the 1620 cm^{-1} shoulder indicating a more classical amyloid behavior for the latter.

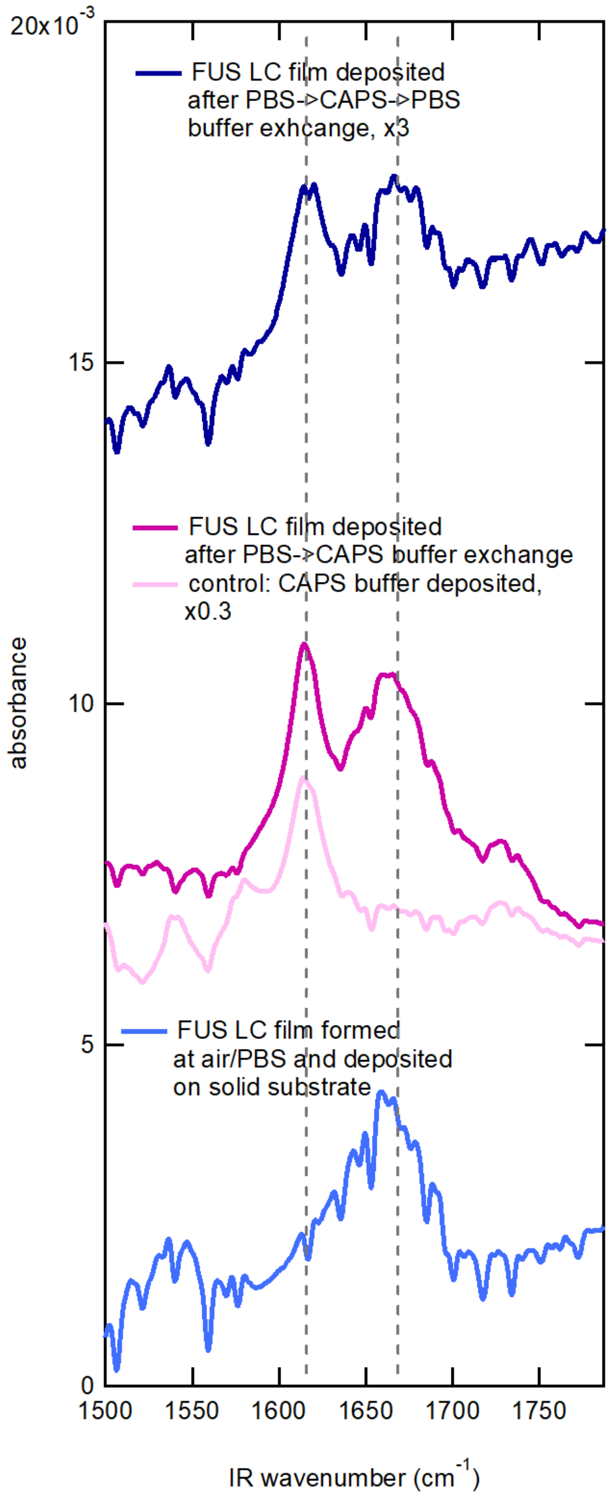

Figure S29. FTIR spectra obtained in transmission for the FUS LC film deposited on a solid substrate (CaF_2 window): (blue) original FUS LC film formed at the air/PBS buffer interface; (purple) that one after the PBS→CAPS buffer exchange and the following deposition of the film; (dark blue) original film after the PBS→CAPS→PBS buffer exchange and the following deposition of the film.

XXIII. FUS LC Labelling Protocol.

1. An aliquot of 100 µL of 0.83 mM FUS in CAPS (SP-182) is thawed, and 300 µL HEPES is added which results in volume ~0.2 mM.

2. Dialysis is performed in 50 mM HEPES, pH ~8.4 (in Ready-A-Lyzer 6-8 kDa) (gets cloudy in the cold, but gets transparent at room temp again); the volume increases during dialysis.

3. Protein concentration is determined with spectrophotometer at A280: 0.063 mM.

4. Cy3 label Kit (General electric Q13108): 1 vial is dissolved in 199 µL DMF for 30 nmol amino groups. Using a third of the label kit (33 µL) needs 10 nmol FUS (i.e 160 µL).

5. 33 µL label kit is added to 160 µL FUS in HEPES and shaked at 4°C over night.

6. Excess of dye is removed with Zeba columns 0.5 mL, 7 MWCO.

7. Centrifuge HEPES through the column, discard the flow through.

8. FUS-dye mix is added into sample and centrifuged.

9. Repeat this with a second column.

10. The excess of dye is removed with dialysis: CAPS buffer pH 11; several changes of the outer medium, the last one over night.

11. Bubble test is performed next morning.

12. 4 µL of 1 mM FUS in CAPS +1 µL labelled FUS + 5 µL MES pH 5.5 → glowing bubbles.

SI References

(1) Burke, K. A.; Janke, A. M.; Rhine, C. L.; Fawzi, N. L. Residue-by-Residue View of In Vitro FUS Granules That Bind the C-Terminal Domain of RNA Polymerase II. *Mol. Cell* **2015**, *60* (2), 231–241. https://doi.org/https://doi.org/10.1016/j.molcel.2015.09.006.

(2) Adochitei, A.; Drochioiu, G. Rapid Characterization of Peptide Secondary Structure by FT-IR Spectroscopy. *Rev. Roum. Chim.* **2011**, *56*, 783–791.

(3) Khurana, R.; Fink, A. L. Do Parallel β-Helix Proteins Have a Unique Fourier Transform Infrared Spectrum? *Biophys. J.* **2000**, *78* (2), 994–1000. https://doi.org/https://doi.org/10.1016/S0006-3495(00)76657-4.

(4) Byler, D. M.; Susi, H. Examination of the Secondary Structure of Proteins by Deconvolved FTIR Spectra. *Biopolymers* **1986**, *25* (3), 469–487. https://doi.org/https://doi.org/10.1002/bip.360250307.

(5) Tan, J.; Zhang, J.; Luo, Y.; Ye, S. Misfolding of a Human Islet Amyloid Polypeptide at the Lipid Membrane Populates through β-Sheet Conformers without Involving α-Helical Intermediates. *J. Am. Chem. Soc.* **2019**, *141* (5), 1941–1948. https://doi.org/10.1021/jacs.8b08537.

(6) Meister, K.; Bäumer, A.; Szilvay, G. R.; Paananen, A.; Bakker, H. J. Self-Assembly and Conformational Changes of Hydrophobin Classes at the Air–Water Interface. *J. Phys. Chem. Lett.* **2016**, *7* (20), 4067–4071. https://doi.org/10.1021/acs.jpclett.6b01917.

(7) Tamm, L. K.; Tatulian, S. A. Infrared Spectroscopy of Proteins and Peptides in Lipid Bilayers. *Q. Rev. Biophys.* **1997**, *30* (4), 365–429. https://doi.org/DOI: 10.1017/S0033583597003375.

(8) Barth, A.; Zscherp, C. What Vibrations Tell about Proteins. *Q. Rev. Biophys.* **2002**, *35* (4), 369–430. https://doi.org/10.1017/S0033583502003815.

(9) Hosseinpour, S.; Roeters, S. J.; Bonn, M.; Peukert, W.; Woutersen, S.; Weidner, T. Structure and Dynamics of Interfacial Peptides and Proteins from Vibrational Sum-Frequency Generation Spectroscopy. *Chem. Rev.* **2020**, *120* (7), 3420–3465. https://doi.org/10.1021/acs.chemrev.9b00410.

(10) Lee, M.; Ghosh, U.; Thurber, K. R.; Kato, M.; Tycko, R. Molecular Structure and Interactions within Amyloid-like Fibrils Formed by a Low-Complexity Protein Sequence

from FUS. *Nat. Commun.* **2020**, *11* (1), 5735. https://doi.org/10.1038/s41467-020-19512-3.

(11) Blaudez, D.; Castano, S.; Desbat, B. Chapter 2 - PM-IRRAS at Liquid Interfaces. In *Biointerface Characterization by Advanced IR Spectroscopy*; Pradier, C. M., Chabal, Y. J. B. T.-B. C. by A. I. R. S., Eds.; Elsevier: Amsterdam, 2011; pp 27–55. https://doi.org/https://doi.org/10.1016/B978-0-444-53558-0.00002-3.

(12) Zaborowska, M.; Broniatowski, M.; Wydro, P.; Matyszewska, D.; Bilewicz, R. Structural Modifications of Lipid Membranes Exposed to Statins – Langmuir Monolayer and PM-IRRAS Study. *J. Mol. Liq.* **2020**, *313*, 113570. https://doi.org/https://doi.org/10.1016/j.molliq.2020.113570.

(13) Lu, H.; Bellucci, L.; Sun, S.; Qi, D.; Rosa, M.; Berger, R.; Corni, S.; Bonn, M. Acidic PH Promotes Refolding and Macroscopic Assembly of Amyloid β (16–22) Peptides at the Air–Water Interface. *J. Phys. Chem. Lett.* **2022**, *13* (29), 6674–6679. https://doi.org/10.1021/acs.jpclett.2c01171.

(14) Sarroukh, R.; Goormaghtigh, E.; Ruysschaert, J.-M.; Raussens, V. ATR-FTIR: A “Rejuvenated” Tool to Investigate Amyloid Proteins. *Biochim. Biophys. Acta - Biomembr.* **2013**, *1828* (10), 2328–2338. https://doi.org/https://doi.org/10.1016/j.bbamem.2013.04.012.

(15) Seki, T.; Sun, S.; Zhong, K.; Yu, C.-C.; Machel, K.; Dreier, L. B.; Backus, E. H. G.; Bonn, M.; Nagata, Y. Unveiling Heterogeneity of Interfacial Water through the Water Bending Mode. *J. Phys. Chem. Lett.* **2019**, *10* (21), 6936–6941. https://doi.org/10.1021/acs.jpclett.9b02748.

(16) Gonella, G.; Lütgebaucks, C.; de Beer, A. G. F.; Roke, S. Second Harmonic and Sum-Frequency Generation from Aqueous Interfaces Is Modulated by Interference. *J. Phys. Chem. C* **2016**, *120* (17), 9165–9173. https://doi.org/10.1021/acs.jpcc.5b12453.
