# Supplementary figures and images for: Fibril formation and ordering of disordered FUS LC driven by hydrophobic interactions
Source: Nat Chem. 2023 May 25;15(8):1146–54. doi: 10.1038/s41557-023-01221-1 (PMC10396963; doi:10.1038/s41557-023-01221-1)

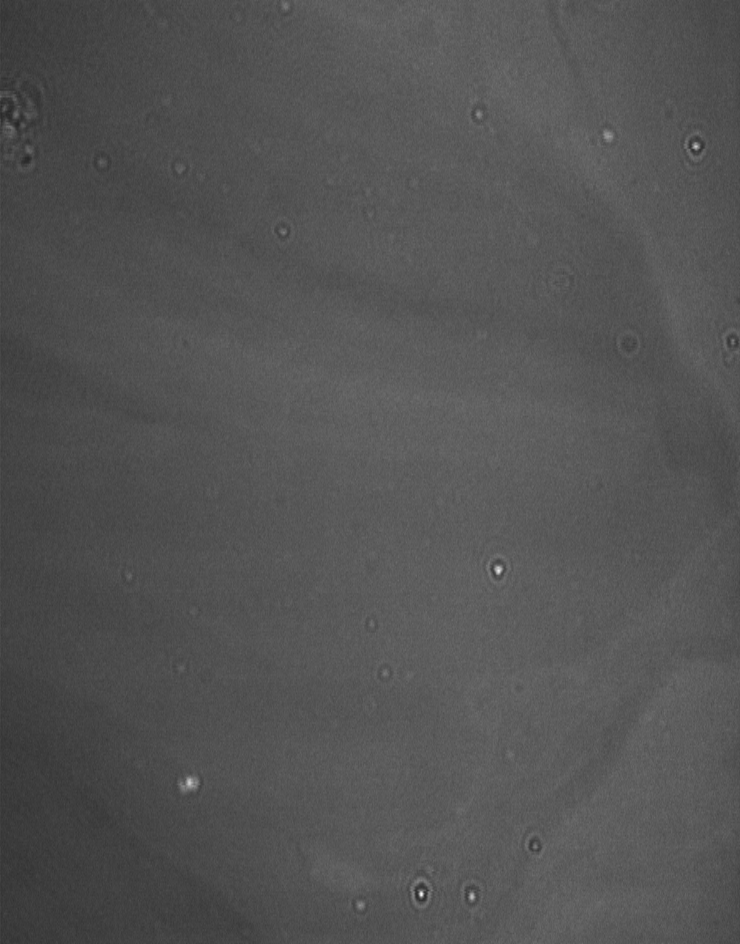

Supplement: Source Data Fig. 1 — SP traces, AFM images, BAM images. [file 41557_2023_1221_MOESM2_ESM.zip › Figure1/Figure1_b_c_d/All Data/FUS_PBS_13min.png]

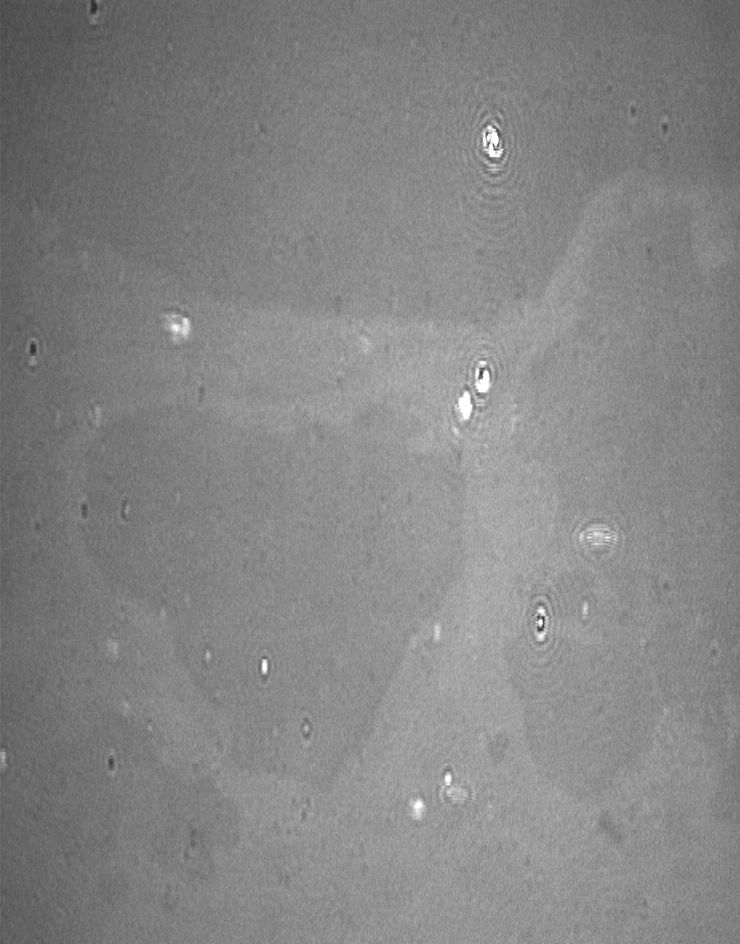

Supplement: Source Data Fig. 1 — SP traces, AFM images, BAM images. [file 41557_2023_1221_MOESM2_ESM.zip › Figure1/Figure1_b_c_d/All Data/FUS_PBS_163min.png]

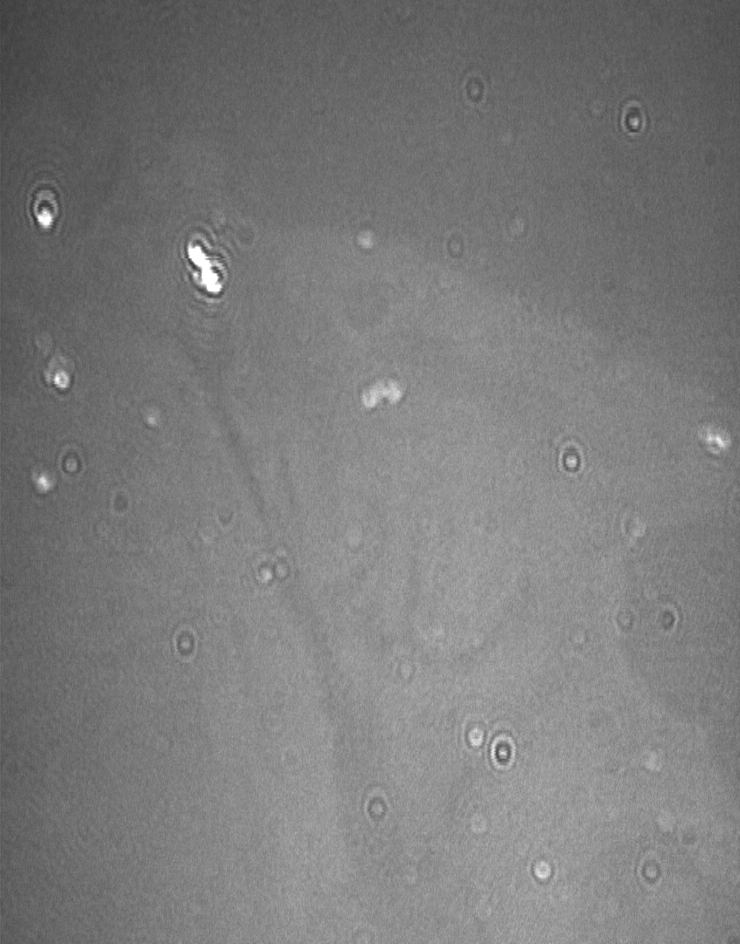

Supplement: Source Data Fig. 1 — SP traces, AFM images, BAM images. [file 41557_2023_1221_MOESM2_ESM.zip › Figure1/Figure1_b_c_d/All Data/FUS_PBS_164min.png]

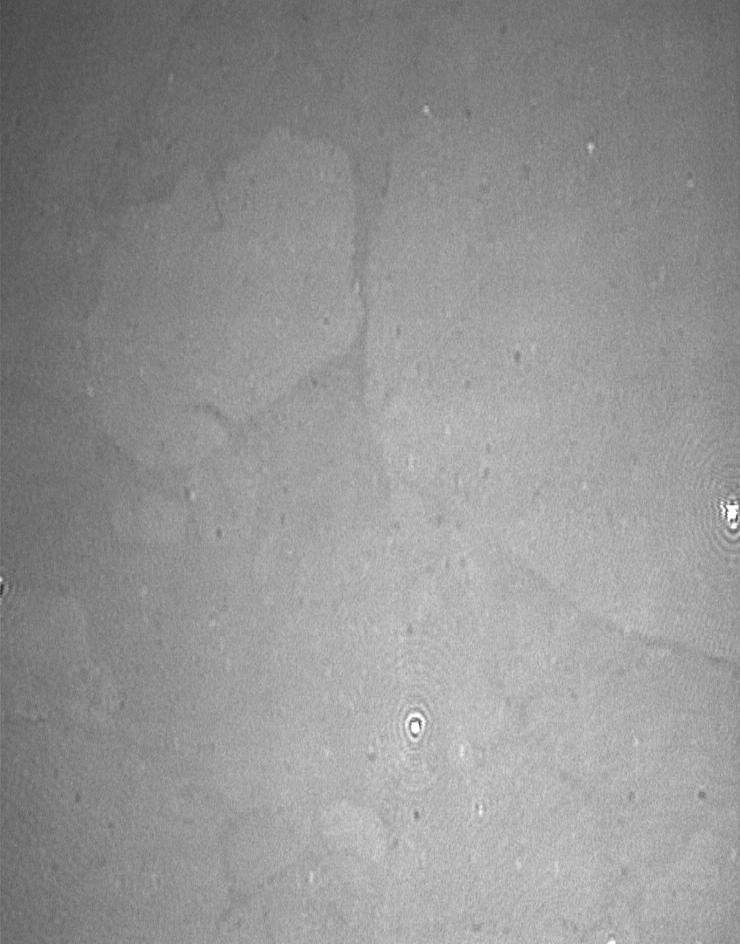

Supplement: Source Data Fig. 1 — SP traces, AFM images, BAM images. [file 41557_2023_1221_MOESM2_ESM.zip › Figure1/Figure1_b_c_d/All Data/FUS_PBS_166min.png]

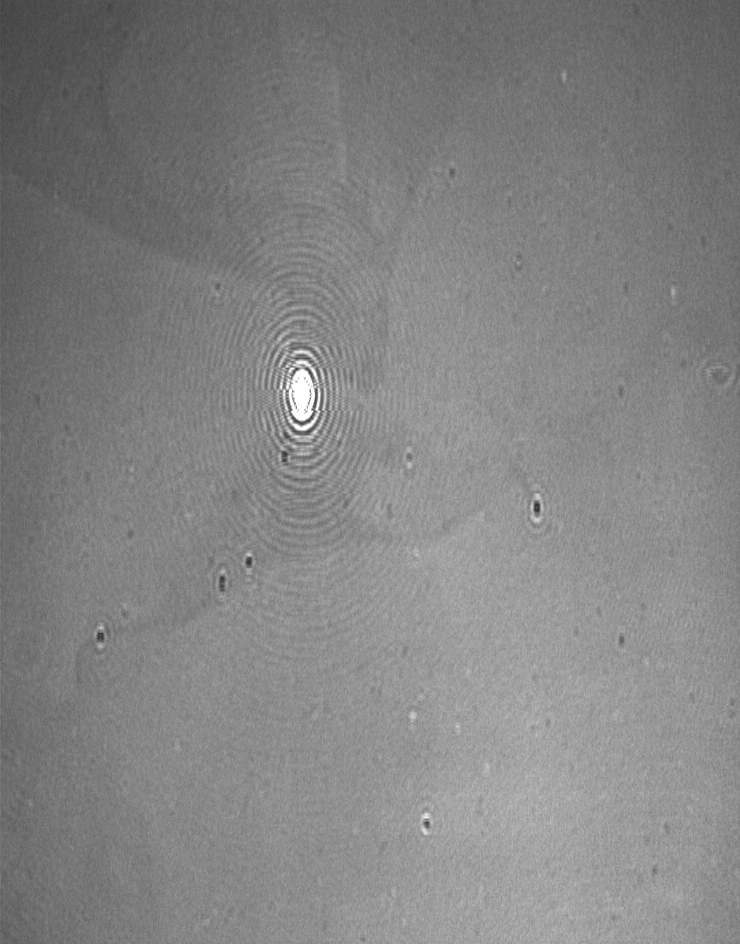

Supplement: Source Data Fig. 1 — SP traces, AFM images, BAM images. [file 41557_2023_1221_MOESM2_ESM.zip › Figure1/Figure1_b_c_d/All Data/FUS_PBS_167min.png]

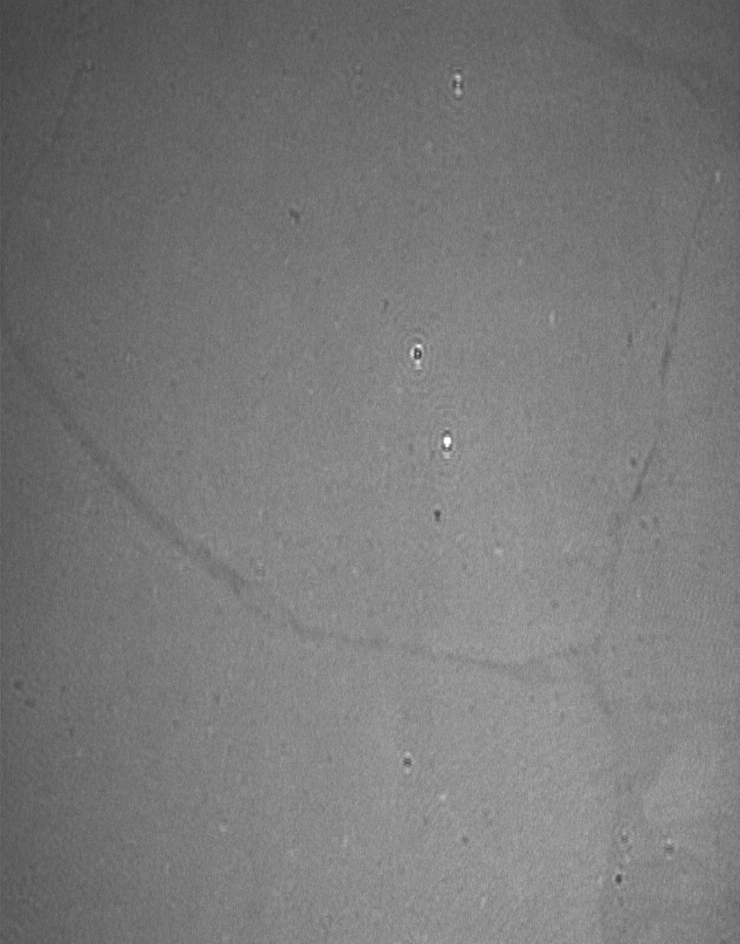

Supplement: Source Data Fig. 1 — SP traces, AFM images, BAM images. [file 41557_2023_1221_MOESM2_ESM.zip › Figure1/Figure1_b_c_d/All Data/FUS_PBS_168min.png]

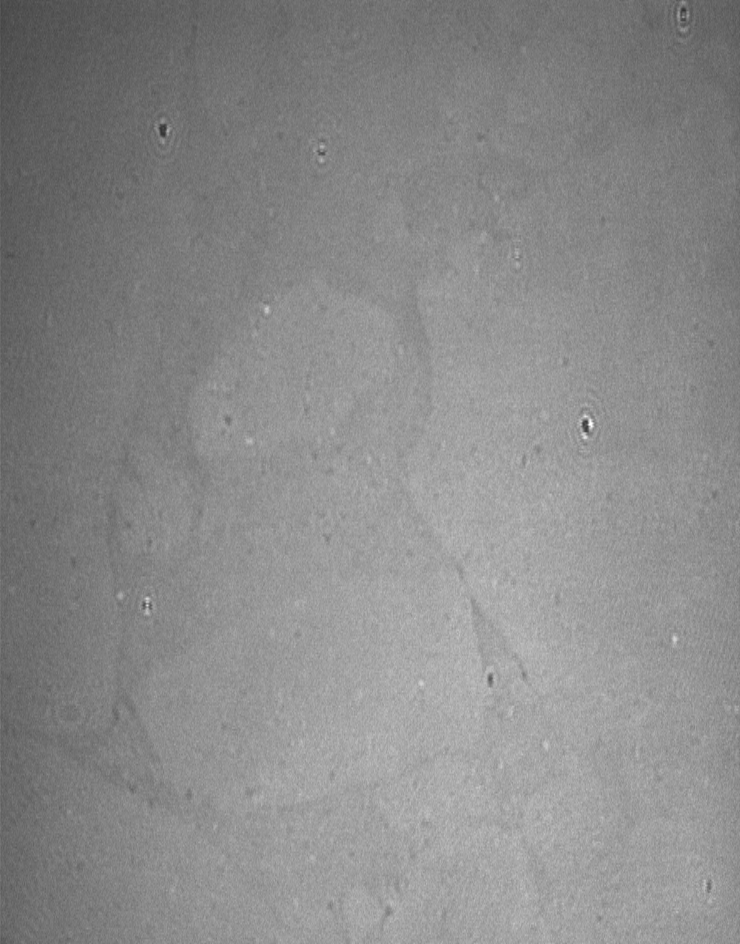

Supplement: Source Data Fig. 1 — SP traces, AFM images, BAM images. [file 41557_2023_1221_MOESM2_ESM.zip › Figure1/Figure1_b_c_d/All Data/FUS_PBS_171min.png]

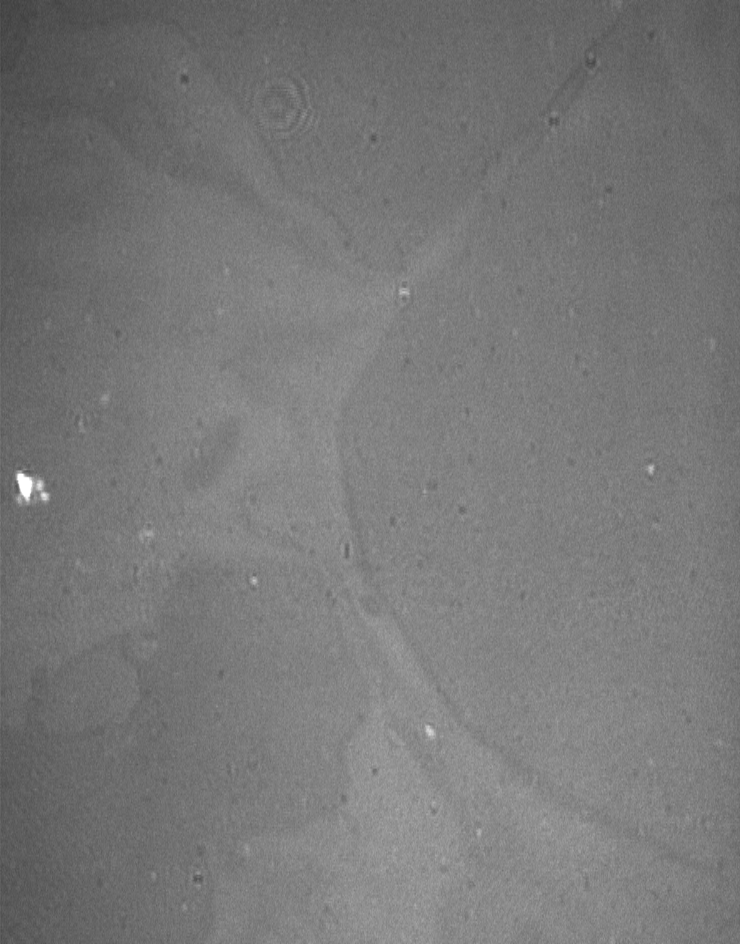

Supplement: Source Data Fig. 1 — SP traces, AFM images, BAM images. [file 41557_2023_1221_MOESM2_ESM.zip › Figure1/Figure1_b_c_d/All Data/FUS_PBS_173min.png]

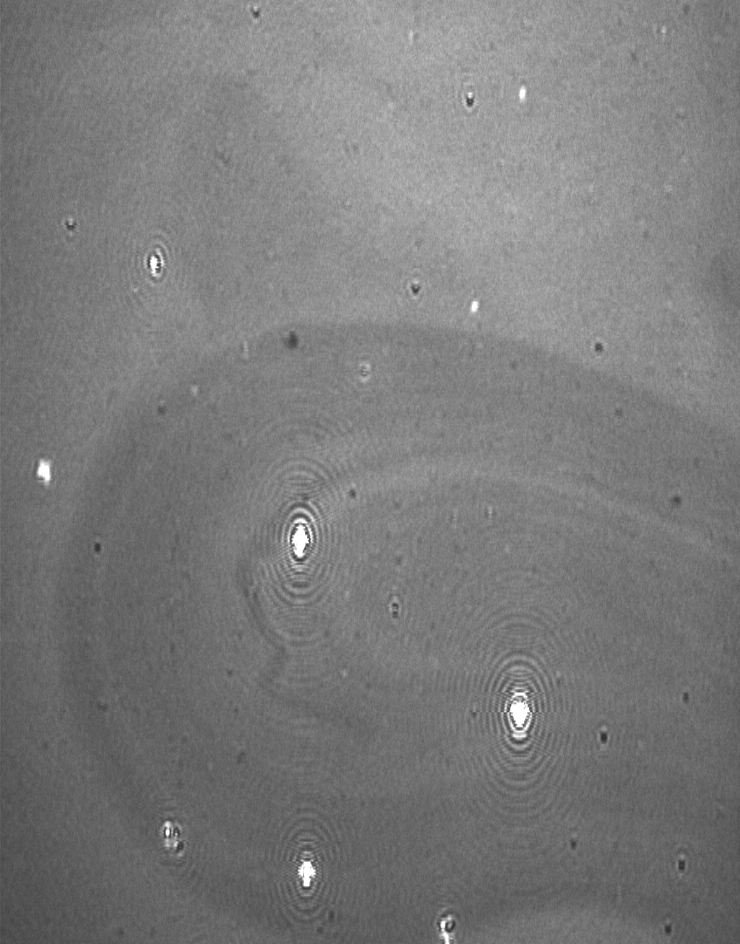

Supplement: Source Data Fig. 1 — SP traces, AFM images, BAM images. [file 41557_2023_1221_MOESM2_ESM.zip › Figure1/Figure1_b_c_d/All Data/FUS_PBS_178min.png]

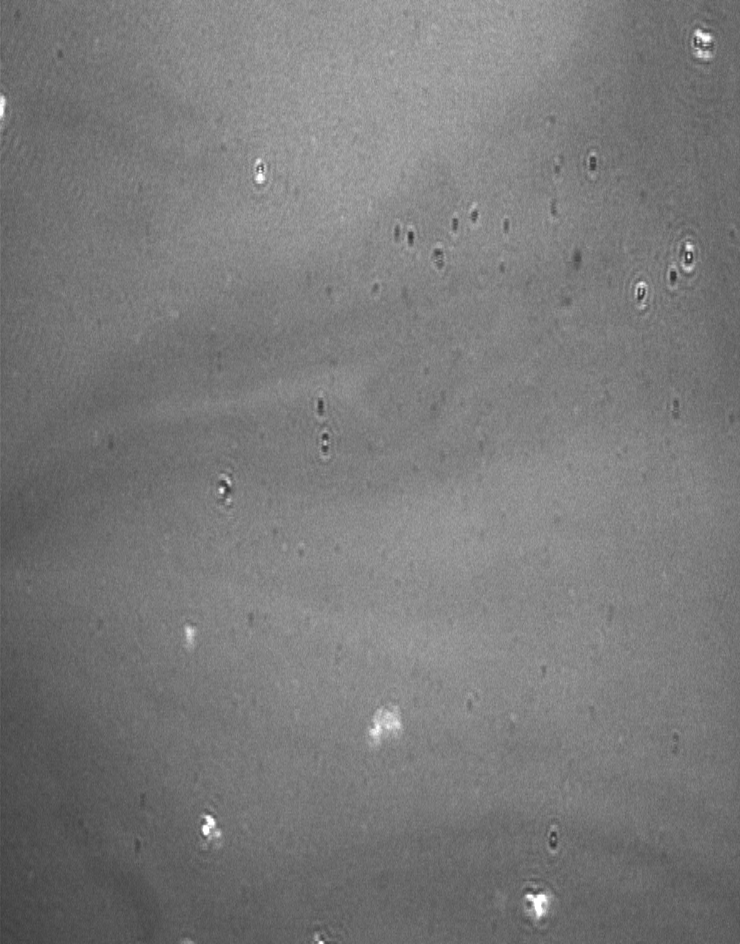

Supplement: Source Data Fig. 1 — SP traces, AFM images, BAM images. [file 41557_2023_1221_MOESM2_ESM.zip › Figure1/Figure1_b_c_d/All Data/FUS_PBS_179min.png]

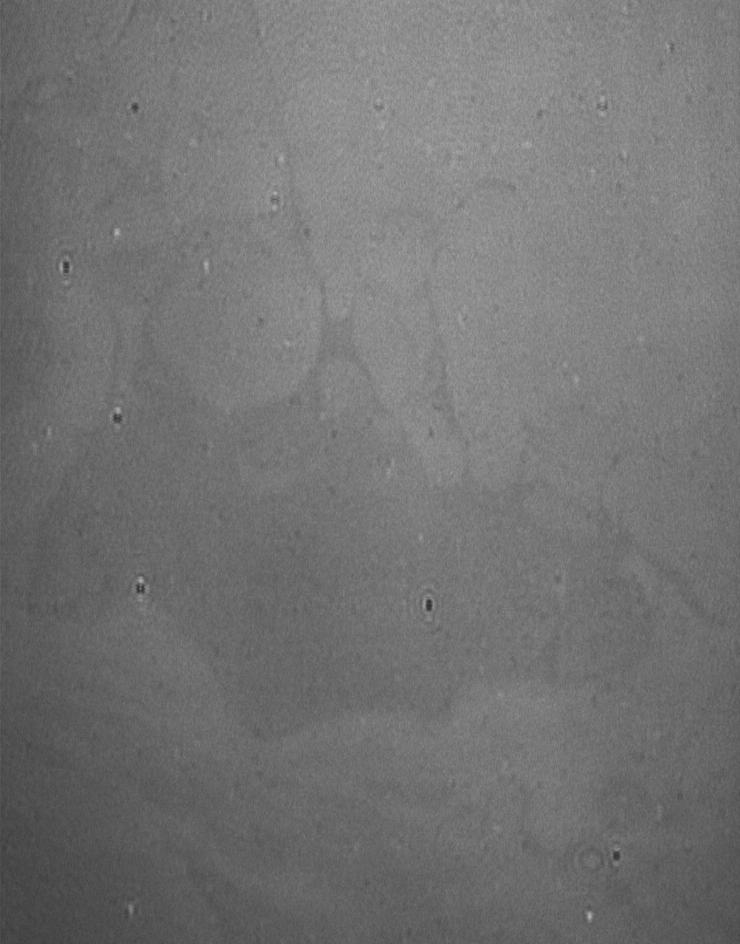

Supplement: Source Data Fig. 1 — SP traces, AFM images, BAM images. [file 41557_2023_1221_MOESM2_ESM.zip › Figure1/Figure1_b_c_d/All Data/FUS_PBS_186min.png]

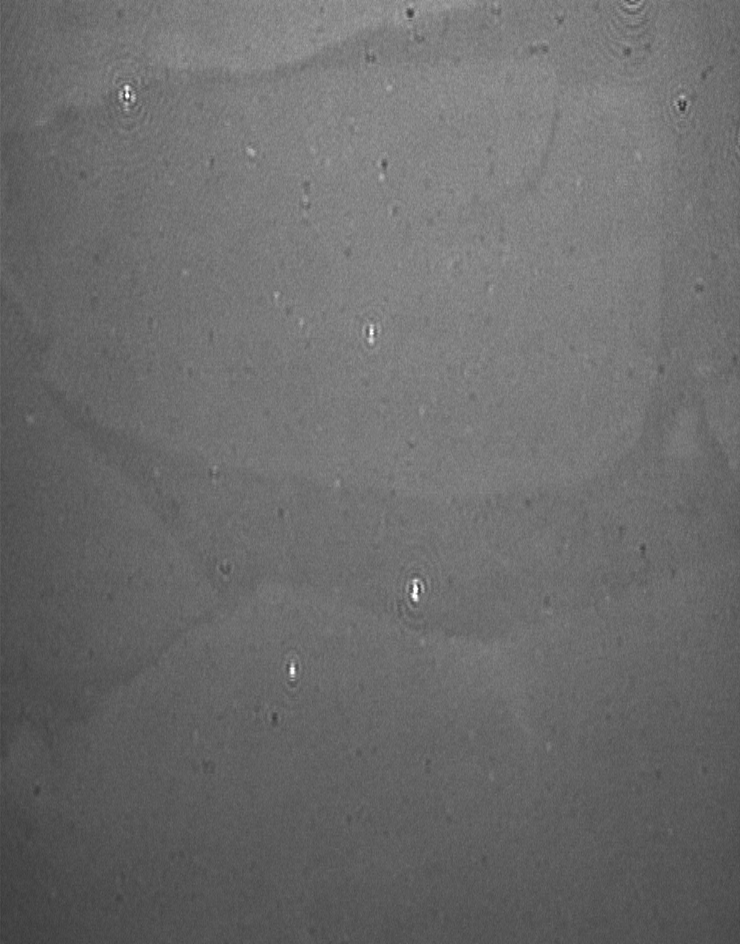

Supplement: Source Data Fig. 1 — SP traces, AFM images, BAM images. [file 41557_2023_1221_MOESM2_ESM.zip › Figure1/Figure1_b_c_d/All Data/FUS_PBS_196min.png]

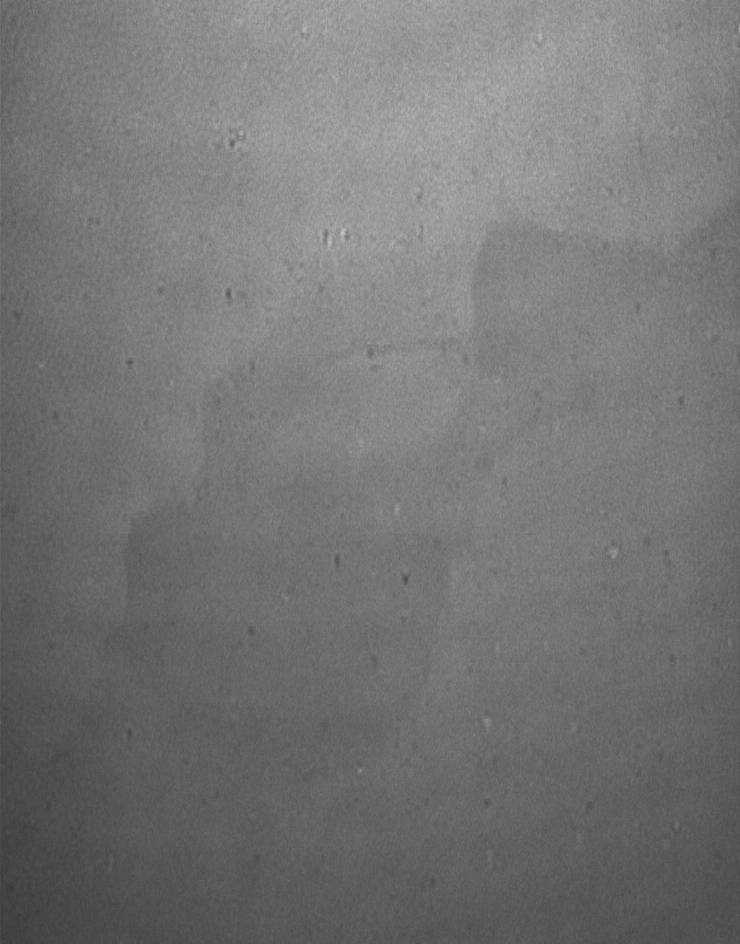

Supplement: Source Data Fig. 1 — SP traces, AFM images, BAM images. [file 41557_2023_1221_MOESM2_ESM.zip › Figure1/Figure1_b_c_d/All Data/FUS_PBS_197min.png]

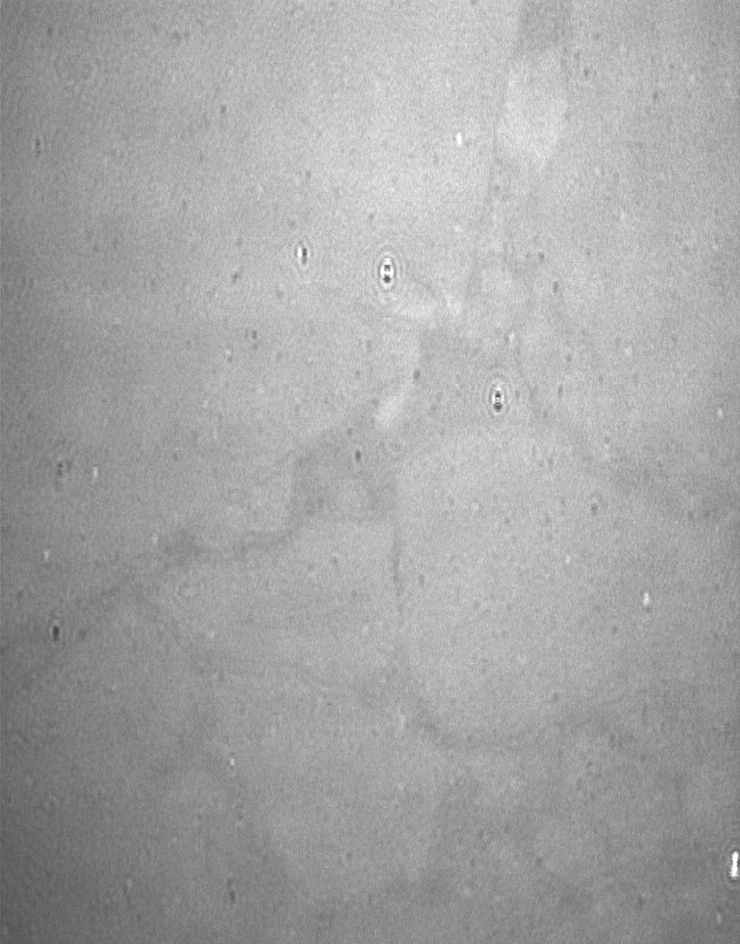

Supplement: Source Data Fig. 1 — SP traces, AFM images, BAM images. [file 41557_2023_1221_MOESM2_ESM.zip › Figure1/Figure1_b_c_d/All Data/FUS_PBS_219min.png]

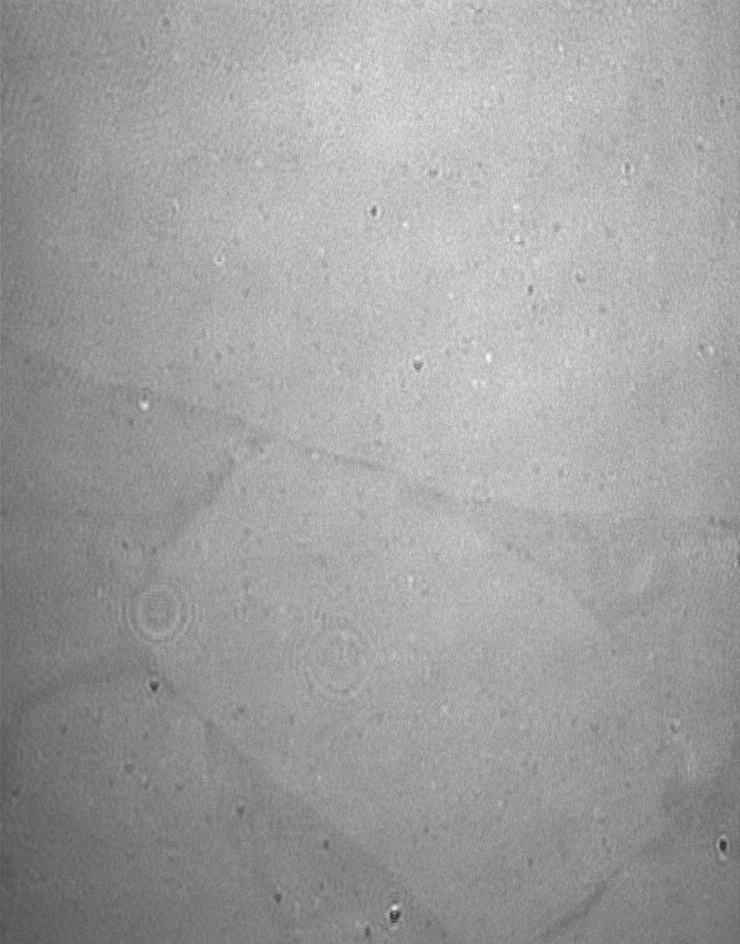

Supplement: Source Data Fig. 1 — SP traces, AFM images, BAM images. [file 41557_2023_1221_MOESM2_ESM.zip › Figure1/Figure1_b_c_d/All Data/FUS_PBS_221min.png]

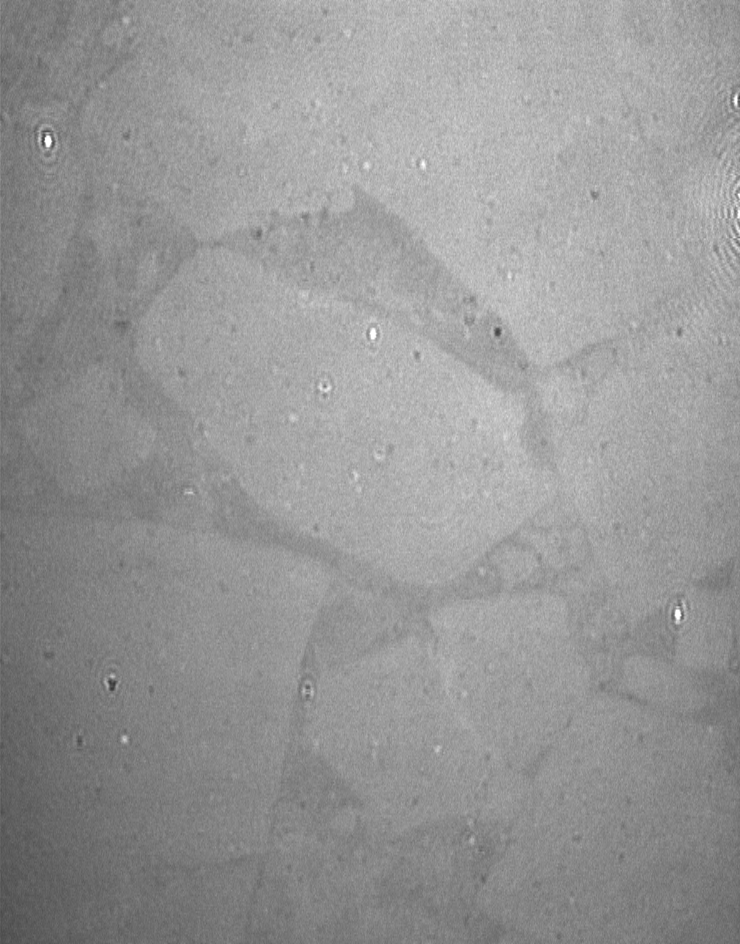

Supplement: Source Data Fig. 1 — SP traces, AFM images, BAM images. [file 41557_2023_1221_MOESM2_ESM.zip › Figure1/Figure1_b_c_d/All Data/FUS_PBS_223min.png]

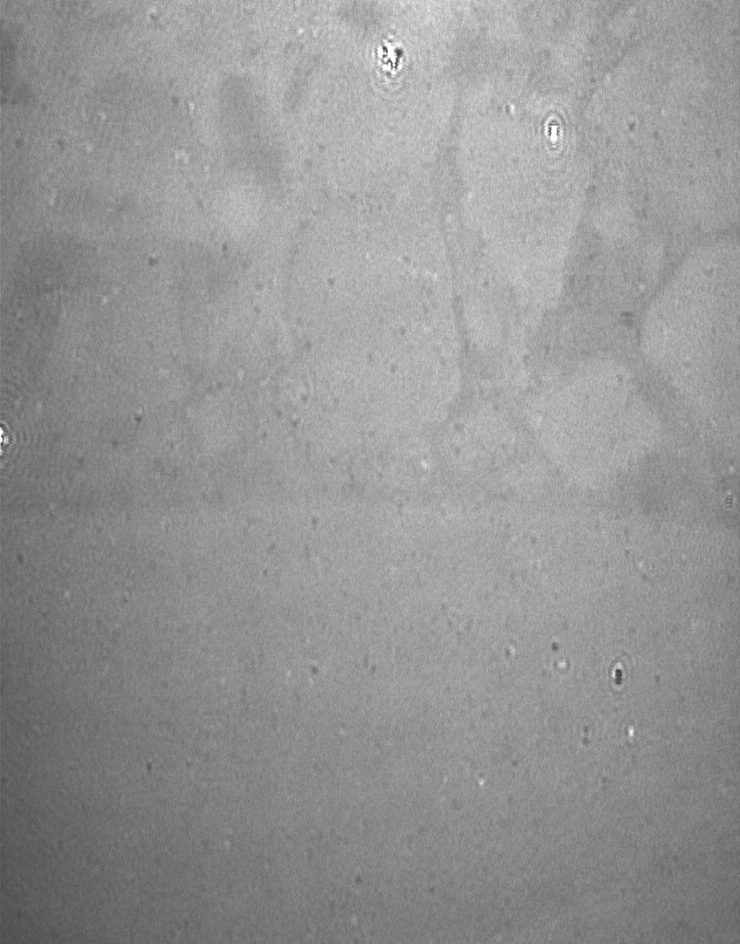

Supplement: Source Data Fig. 1 — SP traces, AFM images, BAM images. [file 41557_2023_1221_MOESM2_ESM.zip › Figure1/Figure1_b_c_d/All Data/FUS_PBS_224min.png]

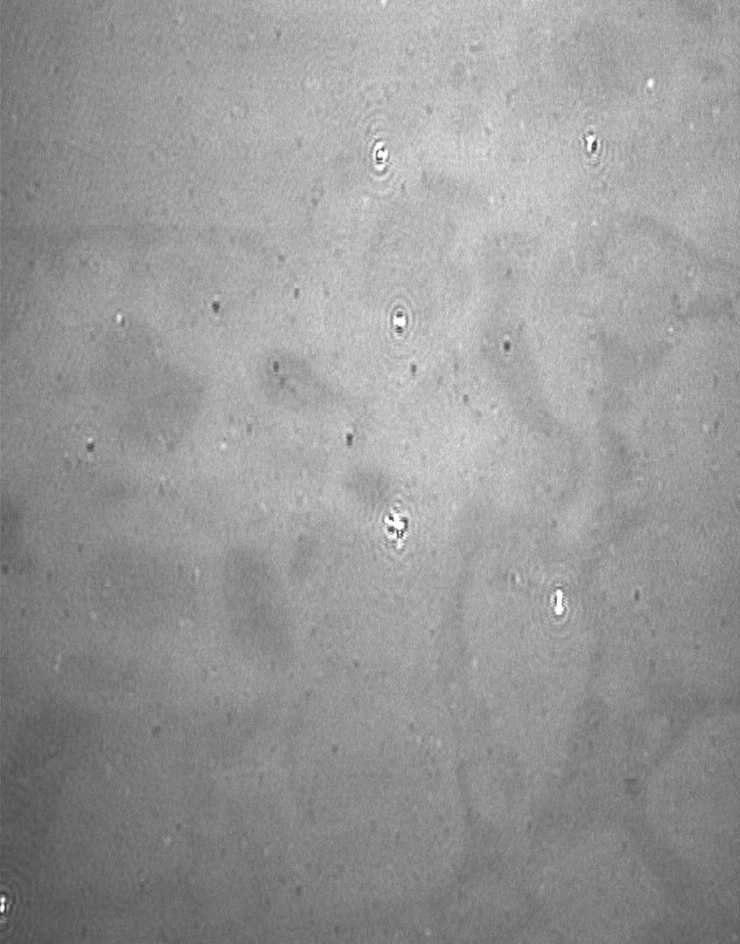

Supplement: Source Data Fig. 1 — SP traces, AFM images, BAM images. [file 41557_2023_1221_MOESM2_ESM.zip › Figure1/Figure1_b_c_d/All Data/FUS_PBS_225min.png]

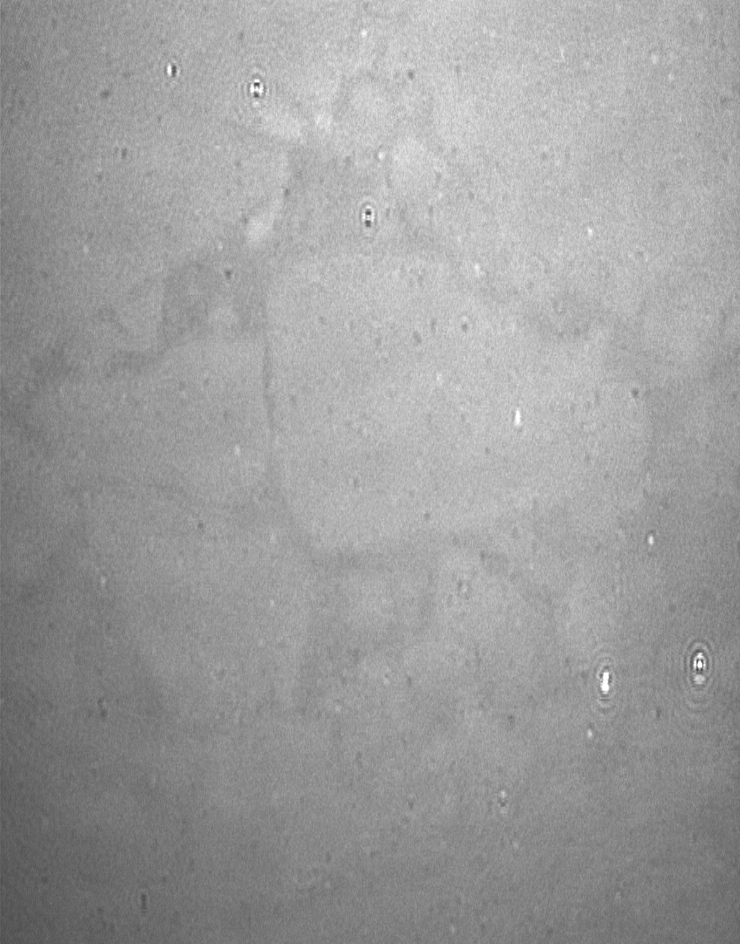

Supplement: Source Data Fig. 1 — SP traces, AFM images, BAM images. [file 41557_2023_1221_MOESM2_ESM.zip › Figure1/Figure1_b_c_d/All Data/FUS_PBS_228min.png]

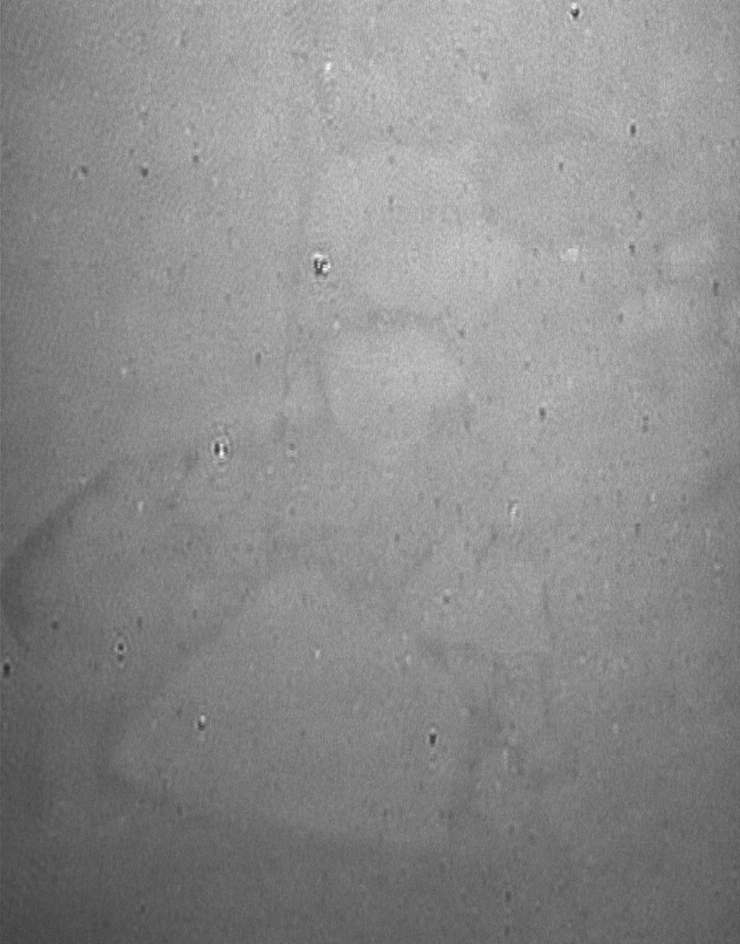

Supplement: Source Data Fig. 1 — SP traces, AFM images, BAM images. [file 41557_2023_1221_MOESM2_ESM.zip › Figure1/Figure1_b_c_d/All Data/FUS_PBS_230min.png]

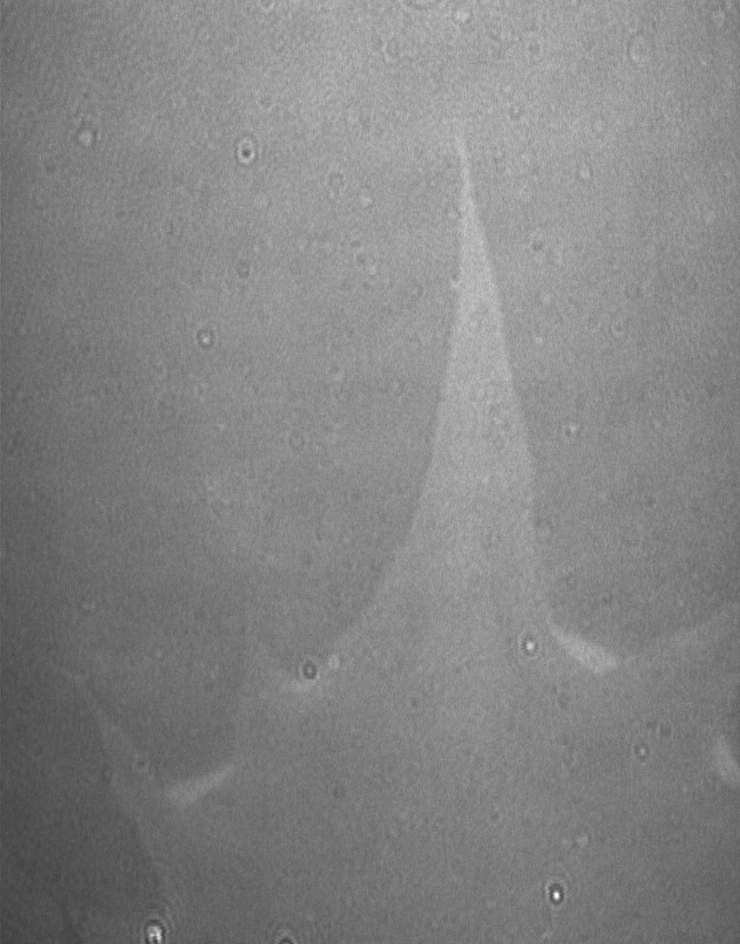

Supplement: Source Data Fig. 1 — SP traces, AFM images, BAM images. [file 41557_2023_1221_MOESM2_ESM.zip › Figure1/Figure1_b_c_d/All Data/FUS_PBS_232min.png]

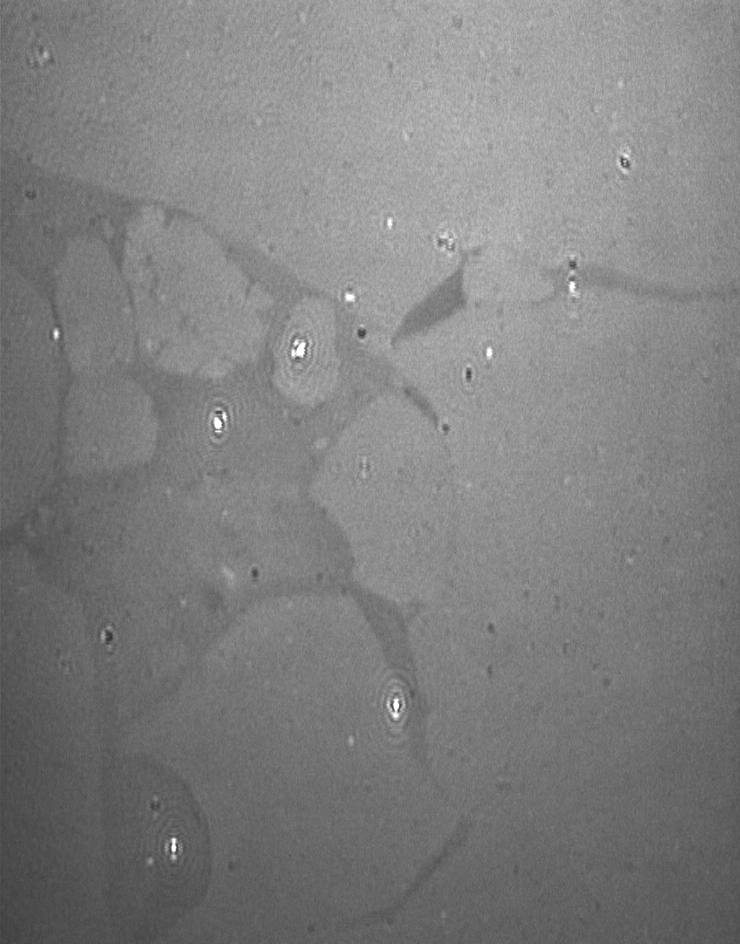

Supplement: Source Data Fig. 1 — SP traces, AFM images, BAM images. [file 41557_2023_1221_MOESM2_ESM.zip › Figure1/Figure1_b_c_d/All Data/FUS_PBS_236min.png]

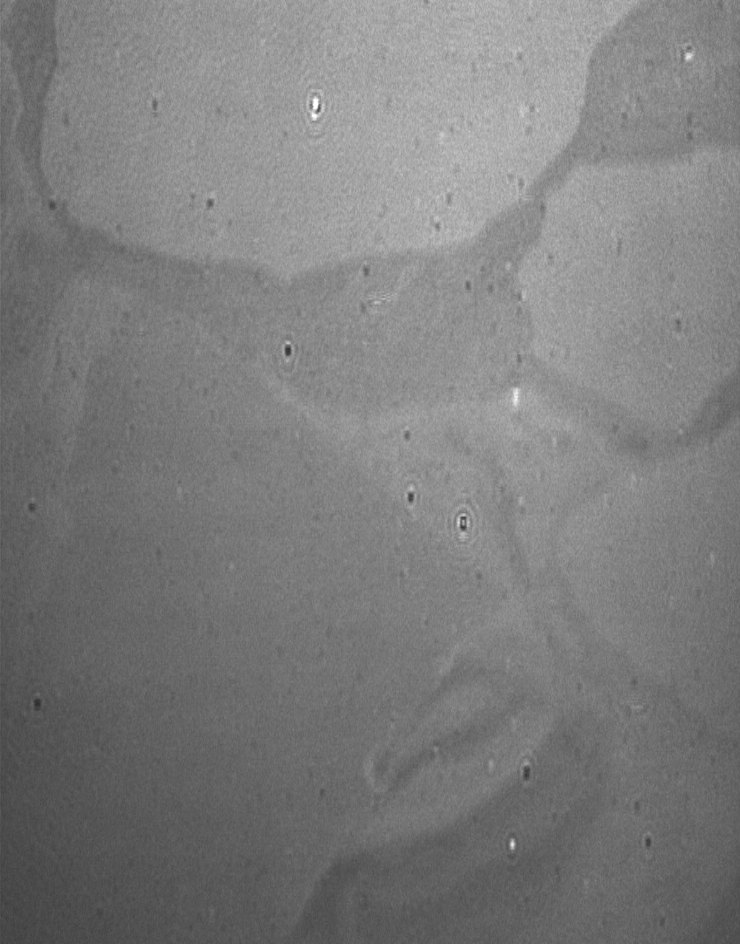

Supplement: Source Data Fig. 1 — SP traces, AFM images, BAM images. [file 41557_2023_1221_MOESM2_ESM.zip › Figure1/Figure1_b_c_d/All Data/FUS_PBS_237min.png]

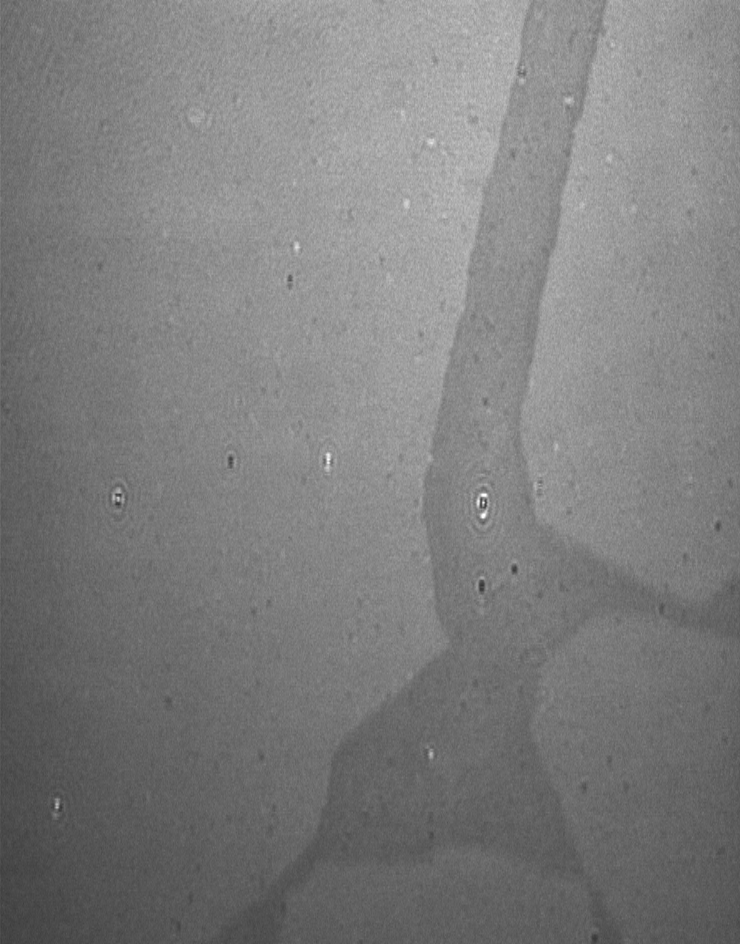

Supplement: Source Data Fig. 1 — SP traces, AFM images, BAM images. [file 41557_2023_1221_MOESM2_ESM.zip › Figure1/Figure1_b_c_d/All Data/FUS_PBS_238min.png]

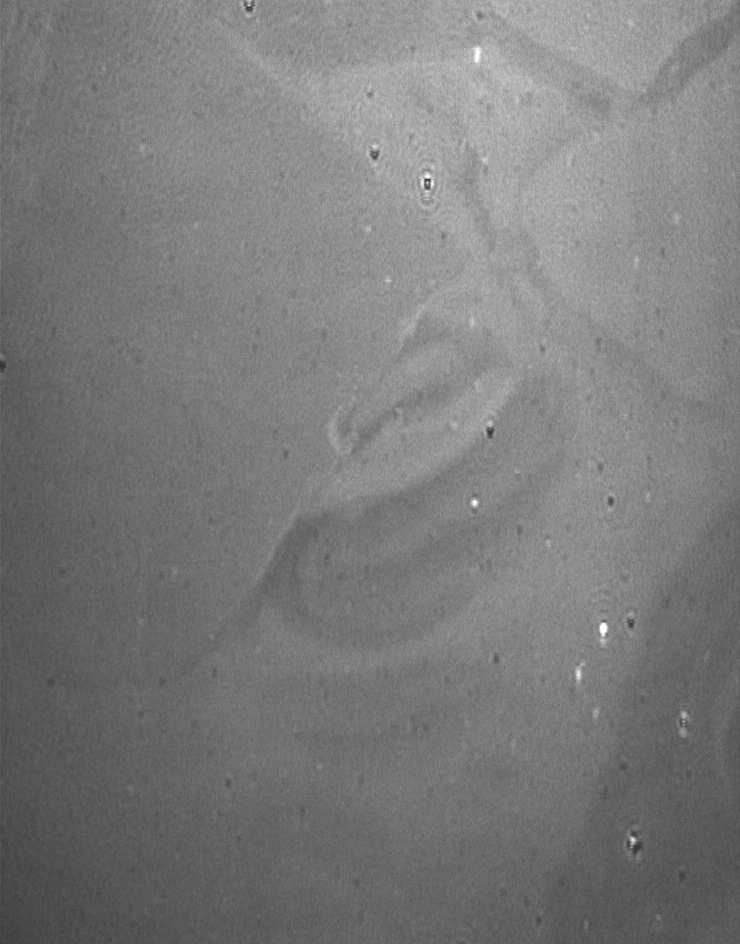

Supplement: Source Data Fig. 1 — SP traces, AFM images, BAM images. [file 41557_2023_1221_MOESM2_ESM.zip › Figure1/Figure1_b_c_d/All Data/FUS_PBS_239min.png]

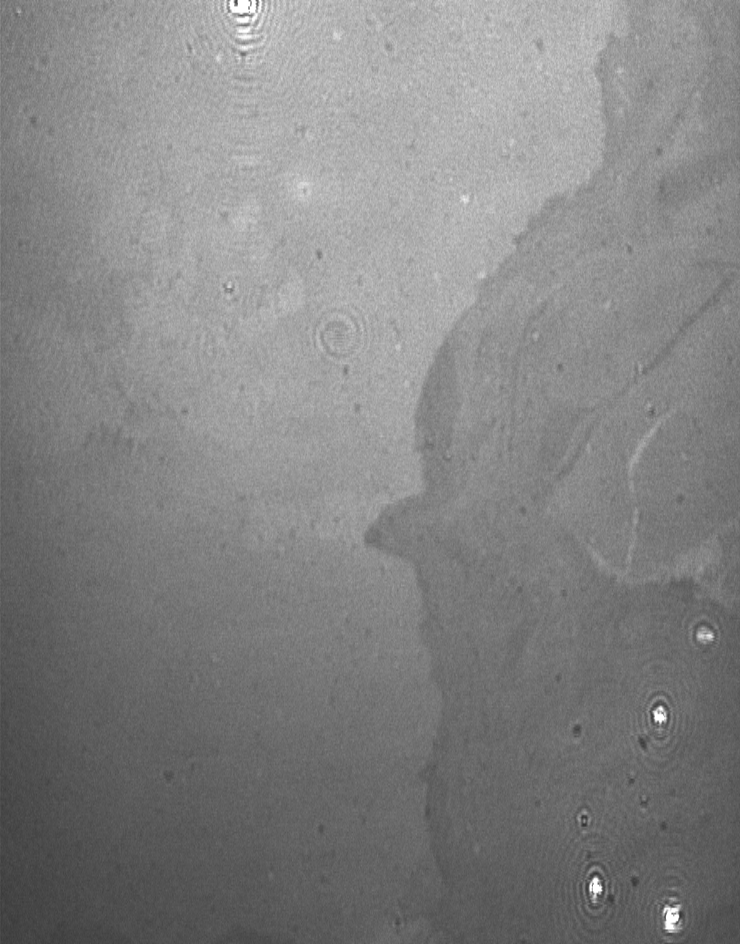

Supplement: Source Data Fig. 1 — SP traces, AFM images, BAM images. [file 41557_2023_1221_MOESM2_ESM.zip › Figure1/Figure1_b_c_d/All Data/FUS_PBS_241min.png]

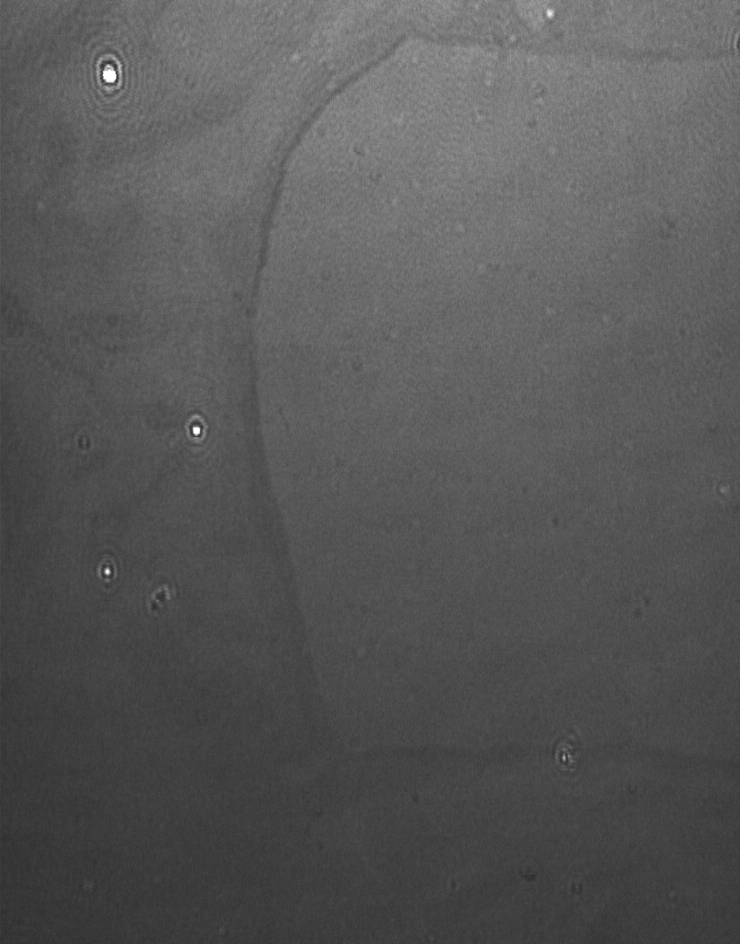

Supplement: Source Data Fig. 1 — SP traces, AFM images, BAM images. [file 41557_2023_1221_MOESM2_ESM.zip › Figure1/Figure1_b_c_d/All Data/FUS_PBS_262min.png]

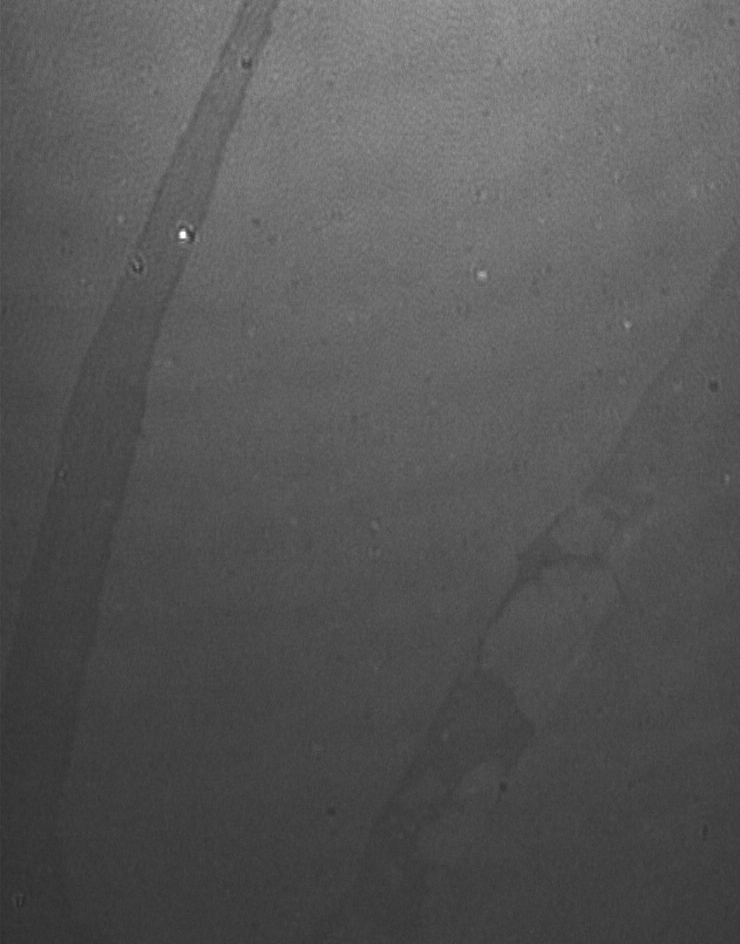

Supplement: Source Data Fig. 1 — SP traces, AFM images, BAM images. [file 41557_2023_1221_MOESM2_ESM.zip › Figure1/Figure1_b_c_d/All Data/FUS_PBS_269min.png]

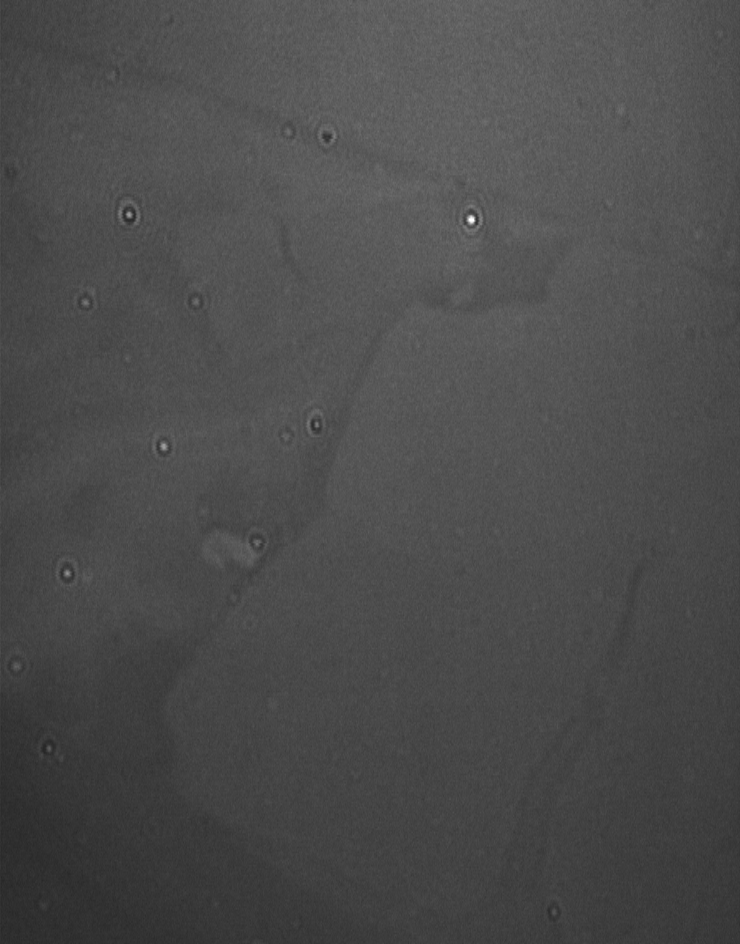

Supplement: Source Data Fig. 1 — SP traces, AFM images, BAM images. [file 41557_2023_1221_MOESM2_ESM.zip › Figure1/Figure1_b_c_d/All Data/FUS_PBS_26min.png]

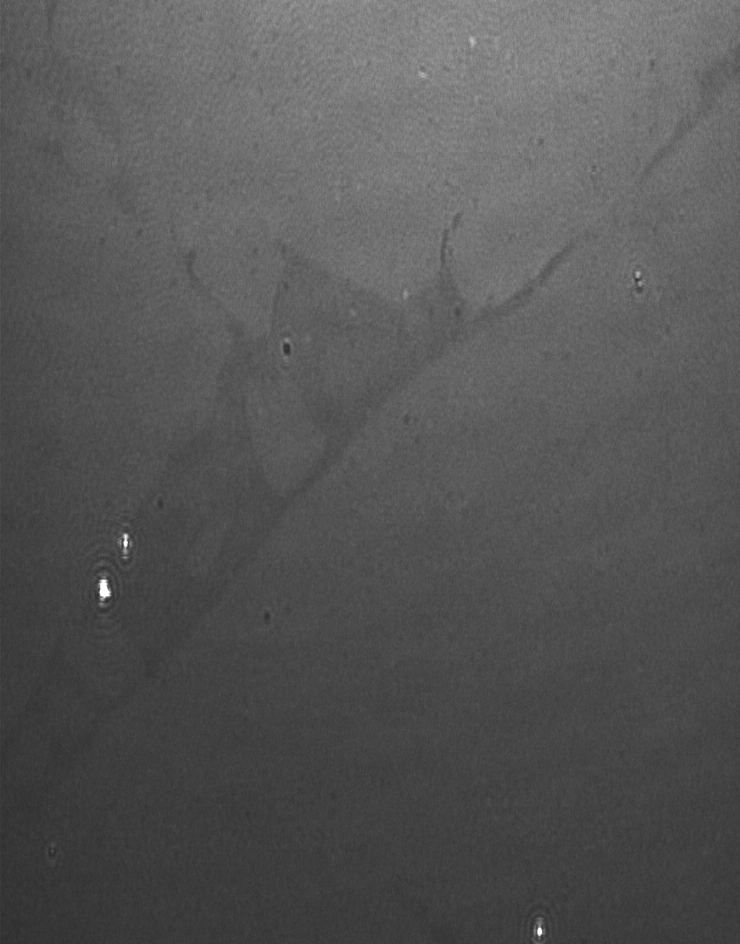

Supplement: Source Data Fig. 1 — SP traces, AFM images, BAM images. [file 41557_2023_1221_MOESM2_ESM.zip › Figure1/Figure1_b_c_d/All Data/FUS_PBS_270min.png]

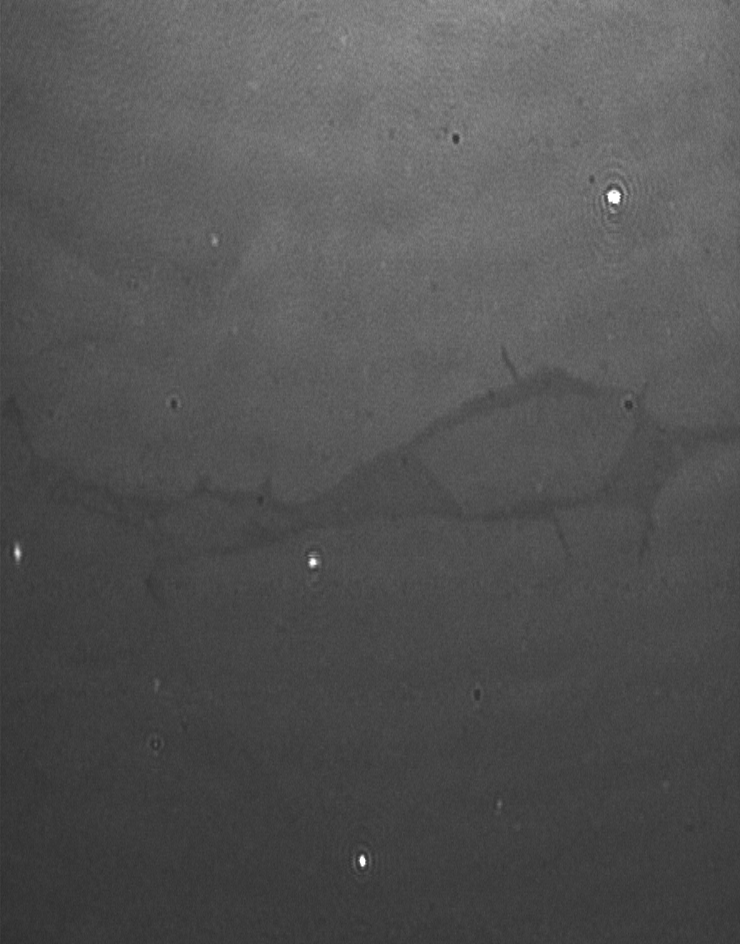

Supplement: Source Data Fig. 1 — SP traces, AFM images, BAM images. [file 41557_2023_1221_MOESM2_ESM.zip › Figure1/Figure1_b_c_d/All Data/FUS_PBS_276min.png]

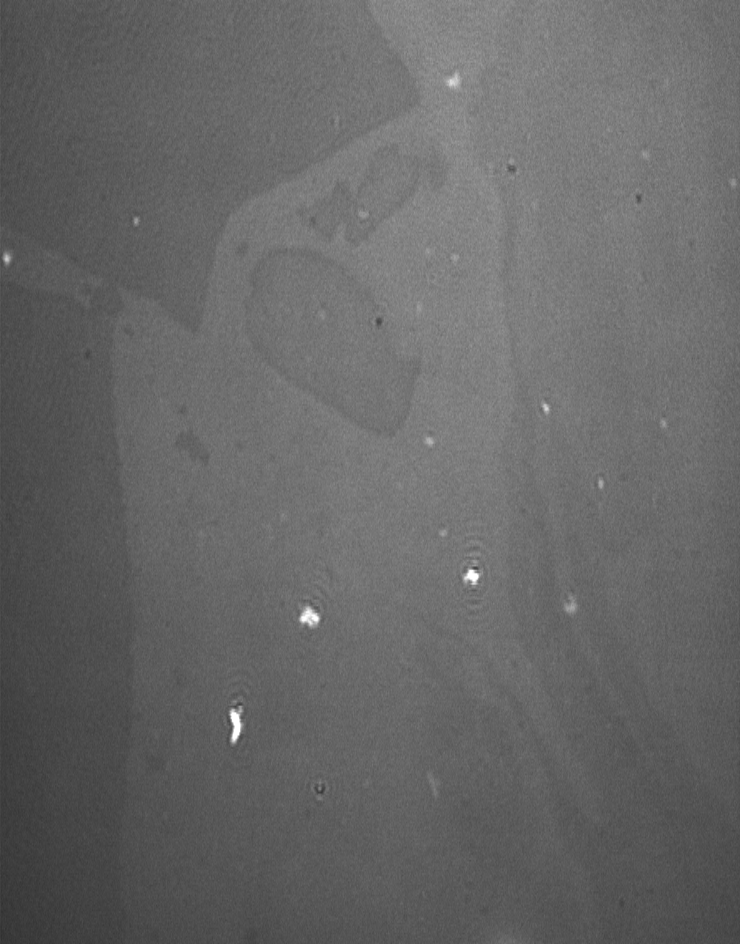

Supplement: Source Data Fig. 1 — SP traces, AFM images, BAM images. [file 41557_2023_1221_MOESM2_ESM.zip › Figure1/Figure1_b_c_d/All Data/FUS_PBS_28min.png]

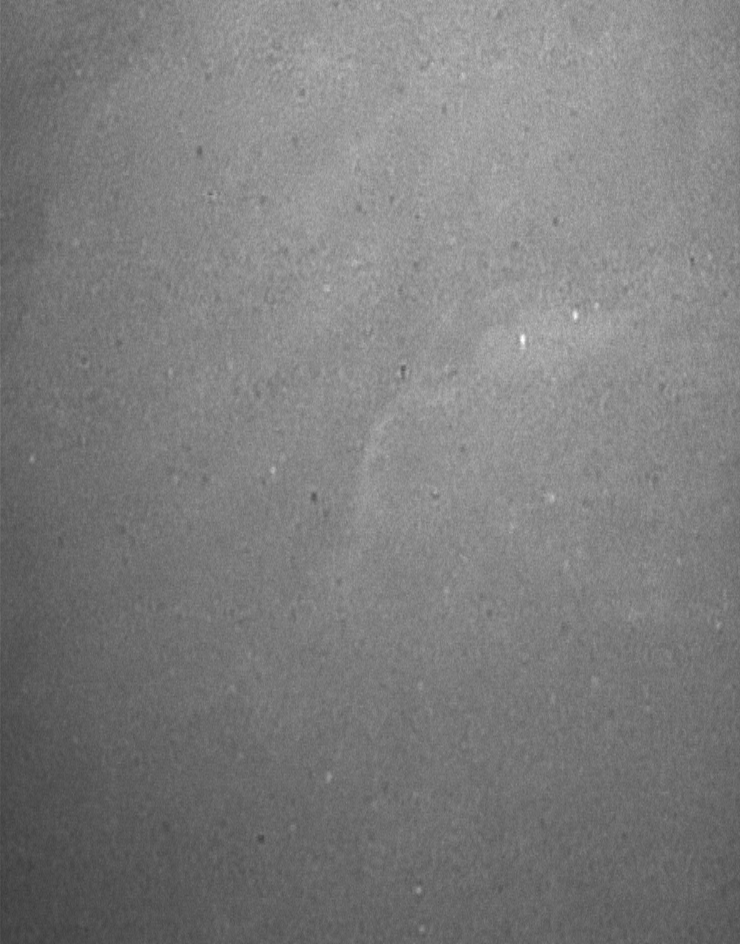

Supplement: Source Data Fig. 1 — SP traces, AFM images, BAM images. [file 41557_2023_1221_MOESM2_ESM.zip › Figure1/Figure1_b_c_d/All Data/FUS_PBS_299min.png]

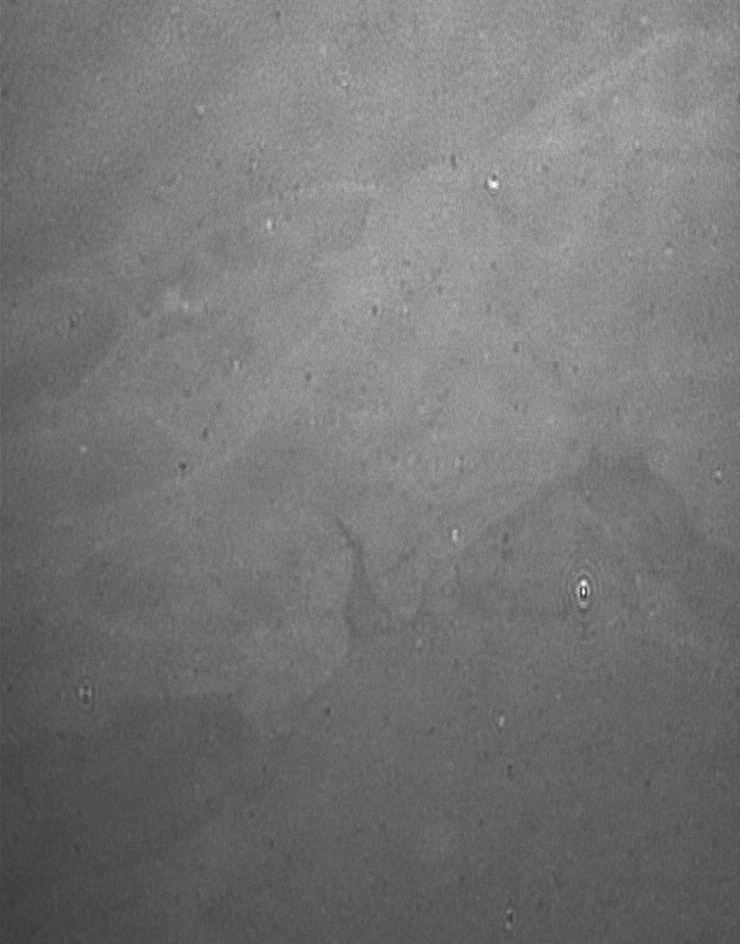

Supplement: Source Data Fig. 1 — SP traces, AFM images, BAM images. [file 41557_2023_1221_MOESM2_ESM.zip › Figure1/Figure1_b_c_d/All Data/FUS_PBS_300min.png]

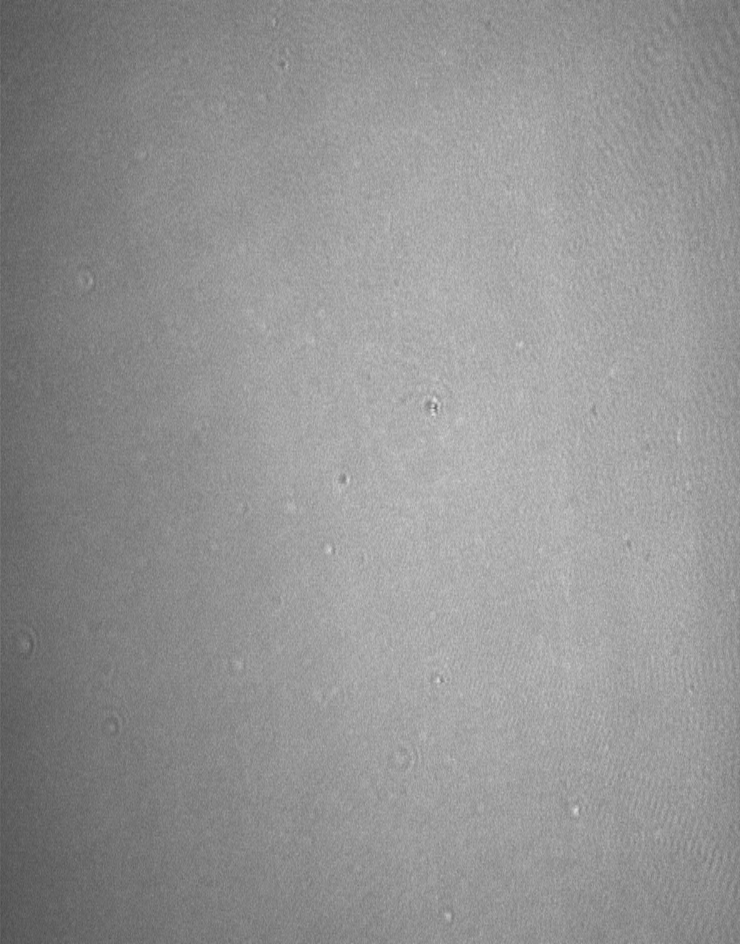

Supplement: Source Data Fig. 1 — SP traces, AFM images, BAM images. [file 41557_2023_1221_MOESM2_ESM.zip › Figure1/Figure1_b_c_d/All Data/FUS_PBS_303min.png]

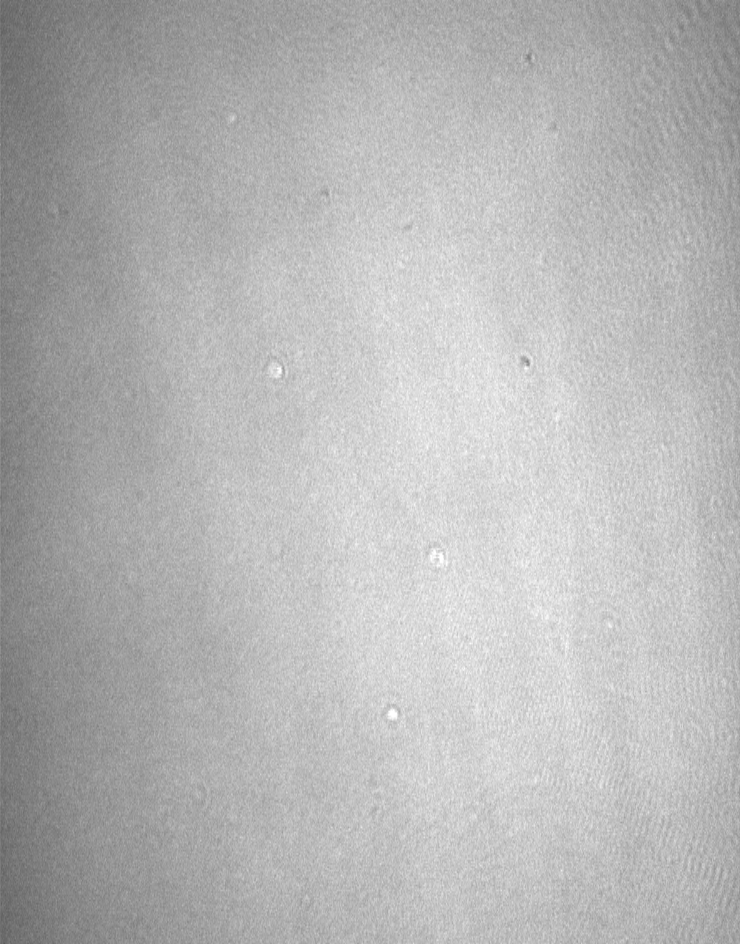

Supplement: Source Data Fig. 1 — SP traces, AFM images, BAM images. [file 41557_2023_1221_MOESM2_ESM.zip › Figure1/Figure1_b_c_d/All Data/FUS_PBS_304min.png]

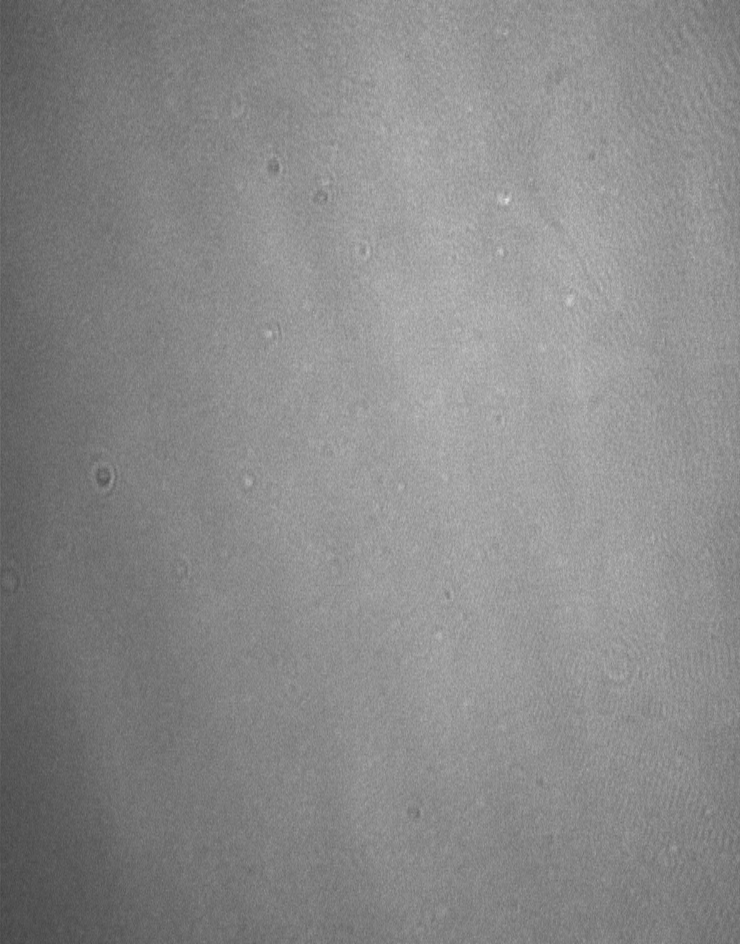

Supplement: Source Data Fig. 1 — SP traces, AFM images, BAM images. [file 41557_2023_1221_MOESM2_ESM.zip › Figure1/Figure1_b_c_d/All Data/FUS_PBS_305min.png]

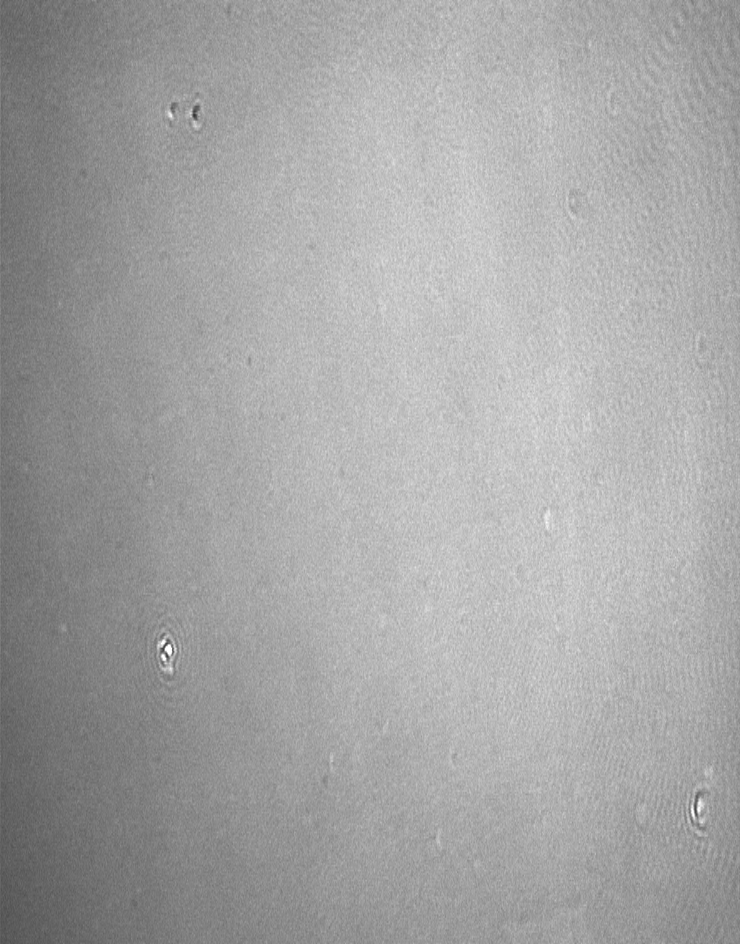

Supplement: Source Data Fig. 1 — SP traces, AFM images, BAM images. [file 41557_2023_1221_MOESM2_ESM.zip › Figure1/Figure1_b_c_d/All Data/FUS_PBS_307min.png]

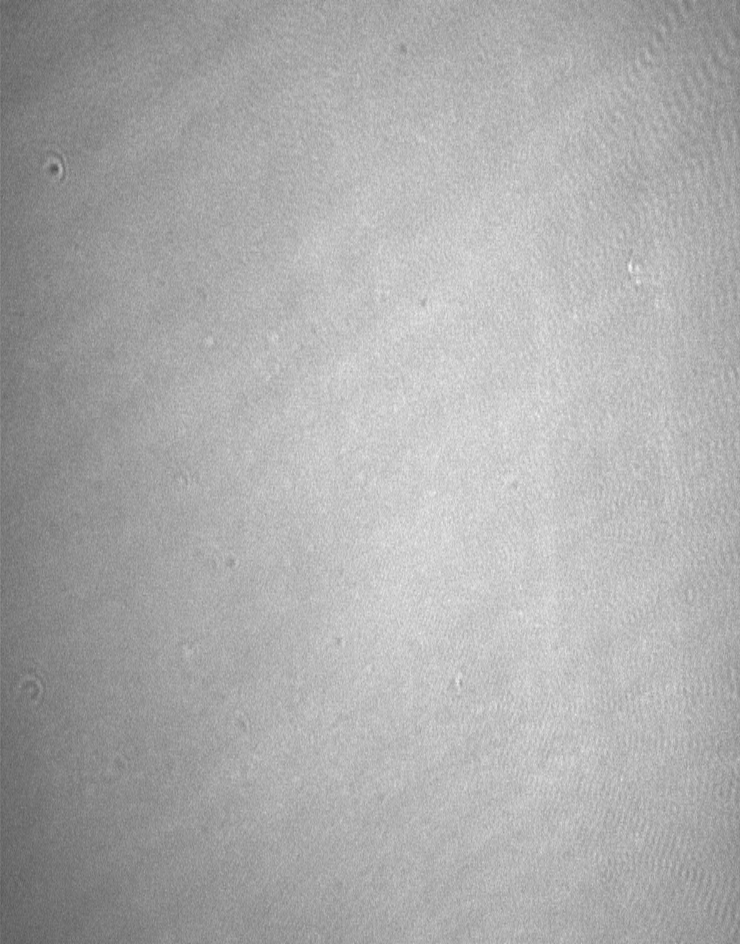

Supplement: Source Data Fig. 1 — SP traces, AFM images, BAM images. [file 41557_2023_1221_MOESM2_ESM.zip › Figure1/Figure1_b_c_d/All Data/FUS_PBS_308min.png]

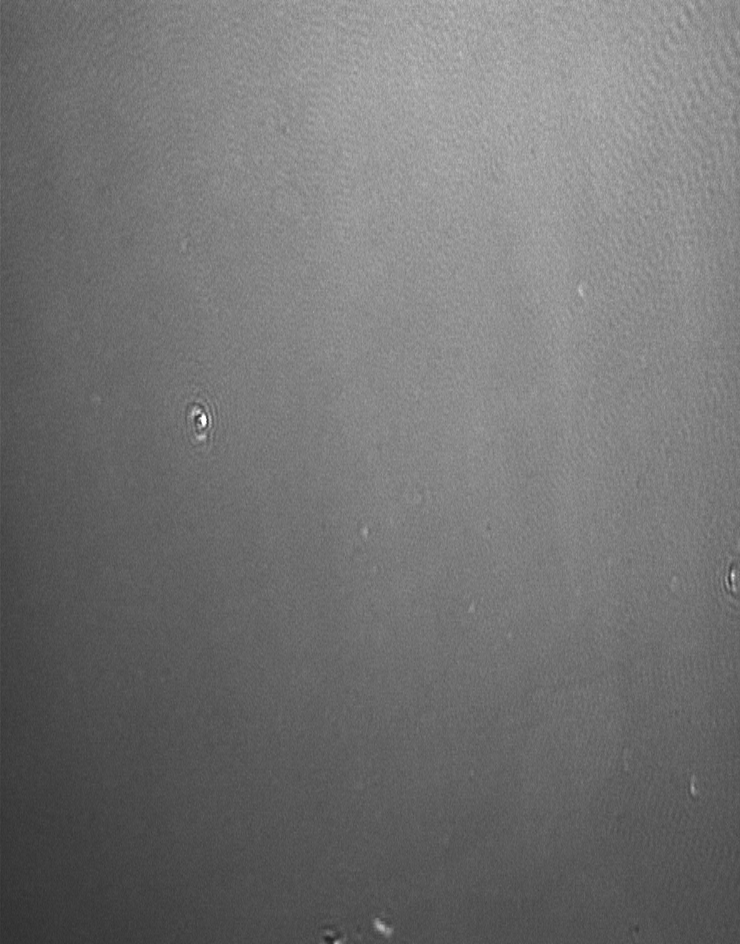

Supplement: Source Data Fig. 1 — SP traces, AFM images, BAM images. [file 41557_2023_1221_MOESM2_ESM.zip › Figure1/Figure1_b_c_d/All Data/FUS_PBS_309min.png]

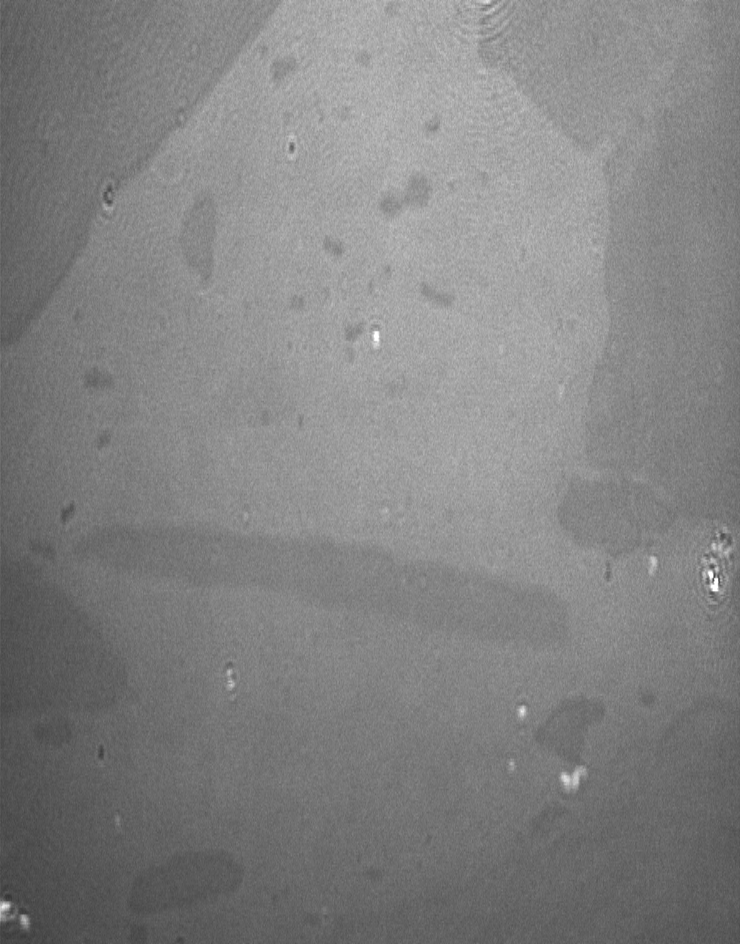

Supplement: Source Data Fig. 1 — SP traces, AFM images, BAM images. [file 41557_2023_1221_MOESM2_ESM.zip › Figure1/Figure1_b_c_d/All Data/FUS_PBS_30min.png]

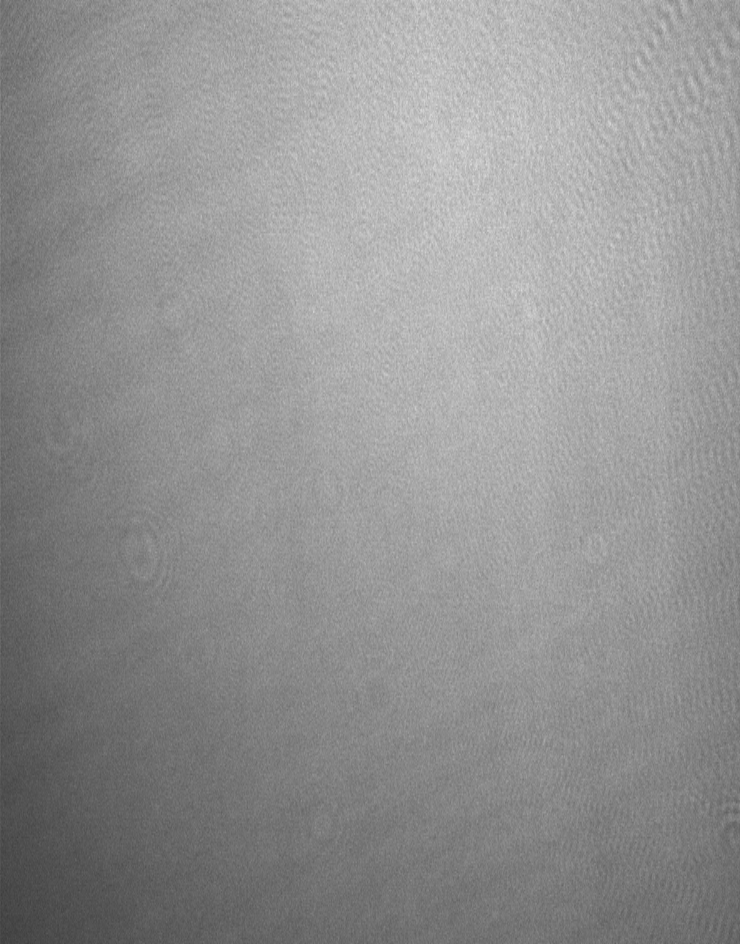

Supplement: Source Data Fig. 1 — SP traces, AFM images, BAM images. [file 41557_2023_1221_MOESM2_ESM.zip › Figure1/Figure1_b_c_d/All Data/FUS_PBS_310min.png]

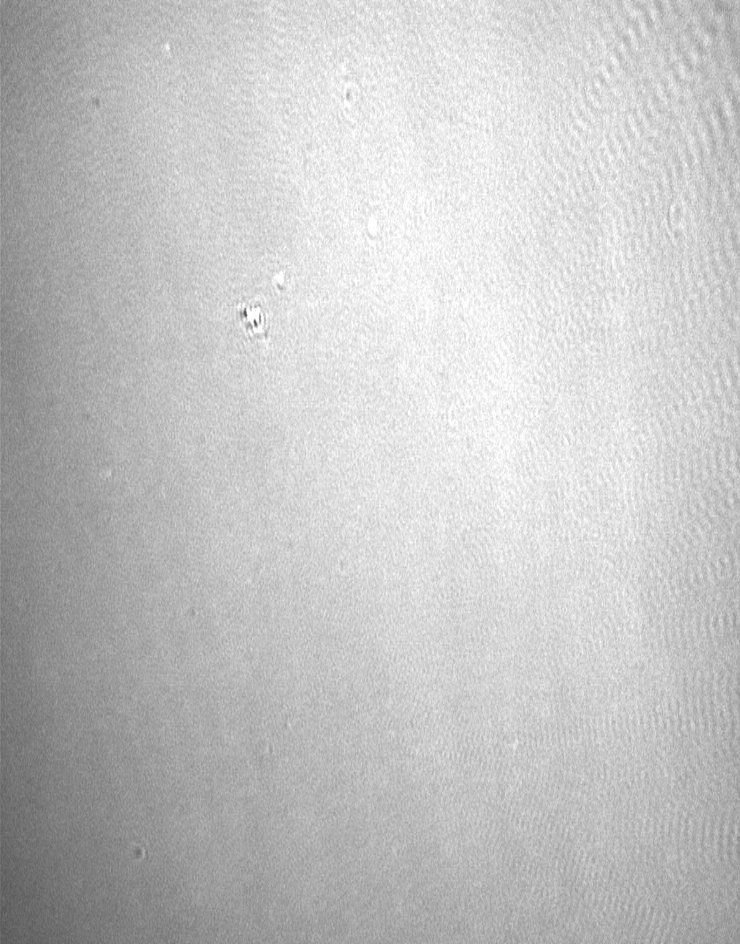

Supplement: Source Data Fig. 1 — SP traces, AFM images, BAM images. [file 41557_2023_1221_MOESM2_ESM.zip › Figure1/Figure1_b_c_d/All Data/FUS_PBS_311min.png]

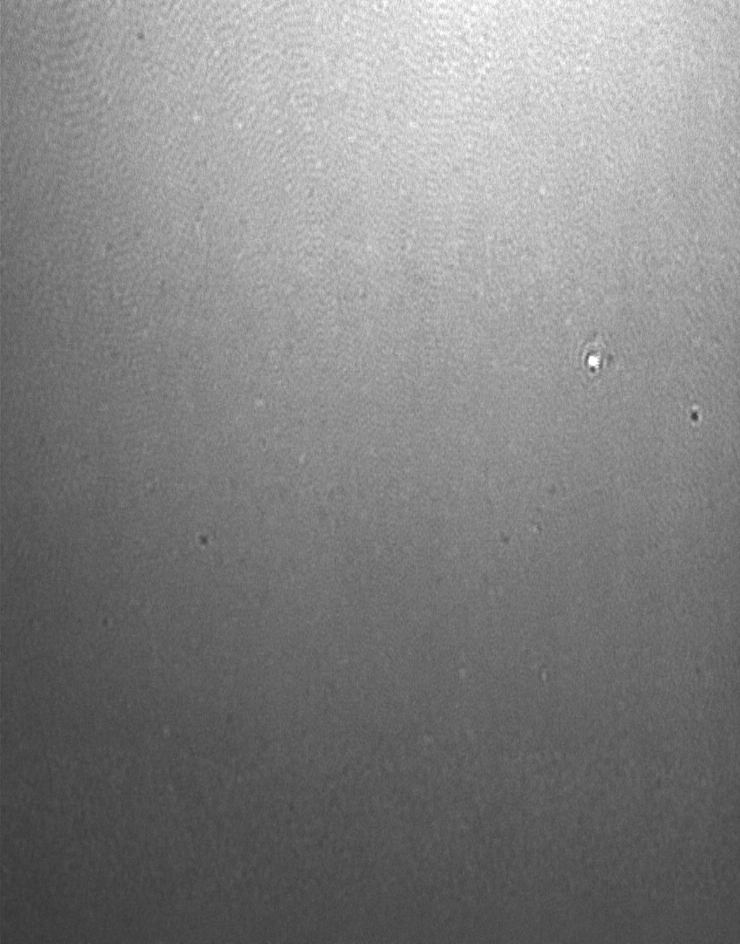

Supplement: Source Data Fig. 1 — SP traces, AFM images, BAM images. [file 41557_2023_1221_MOESM2_ESM.zip › Figure1/Figure1_b_c_d/All Data/FUS_PBS_314min.png]

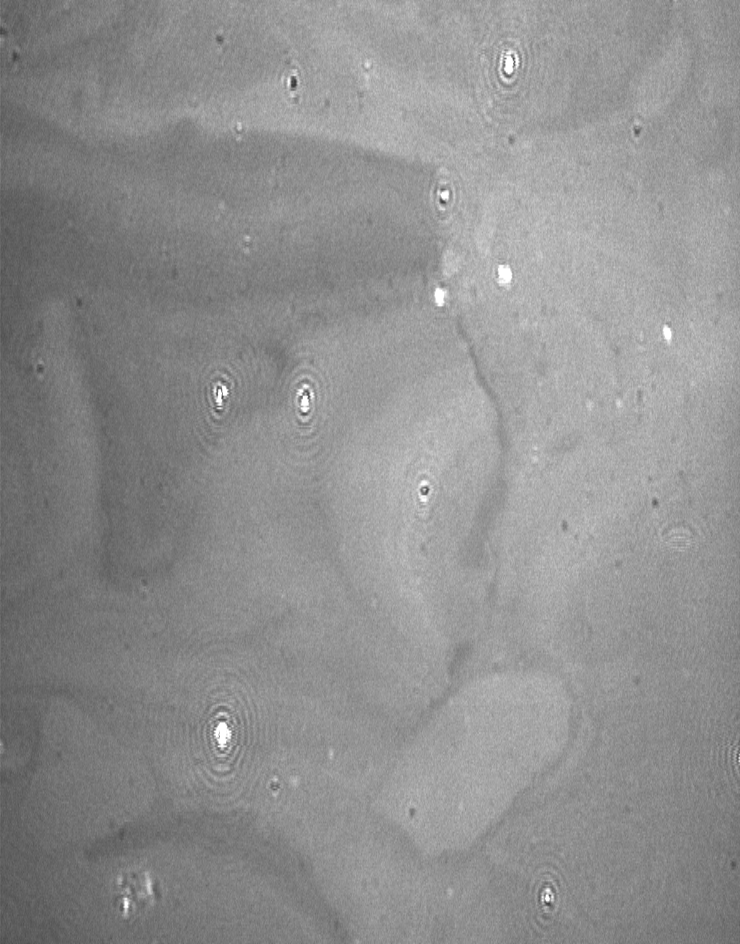

Supplement: Source Data Fig. 1 — SP traces, AFM images, BAM images. [file 41557_2023_1221_MOESM2_ESM.zip › Figure1/Figure1_b_c_d/All Data/FUS_PBS_50min.png]

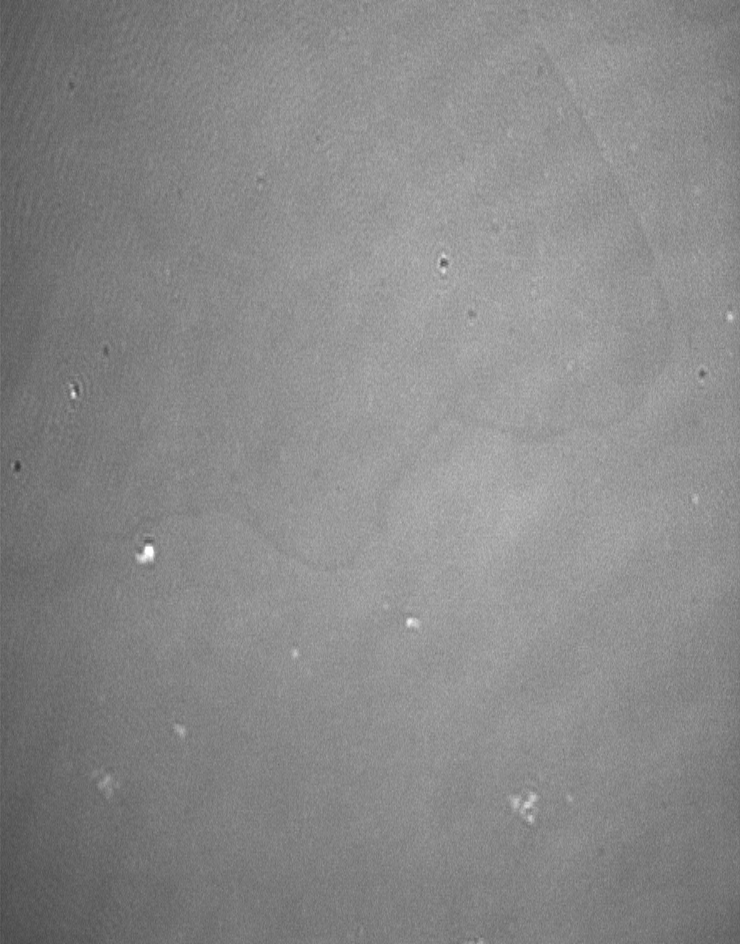

Supplement: Source Data Fig. 1 — SP traces, AFM images, BAM images. [file 41557_2023_1221_MOESM2_ESM.zip › Figure1/Figure1_b_c_d/All Data/FUS_PBS_52min.png]

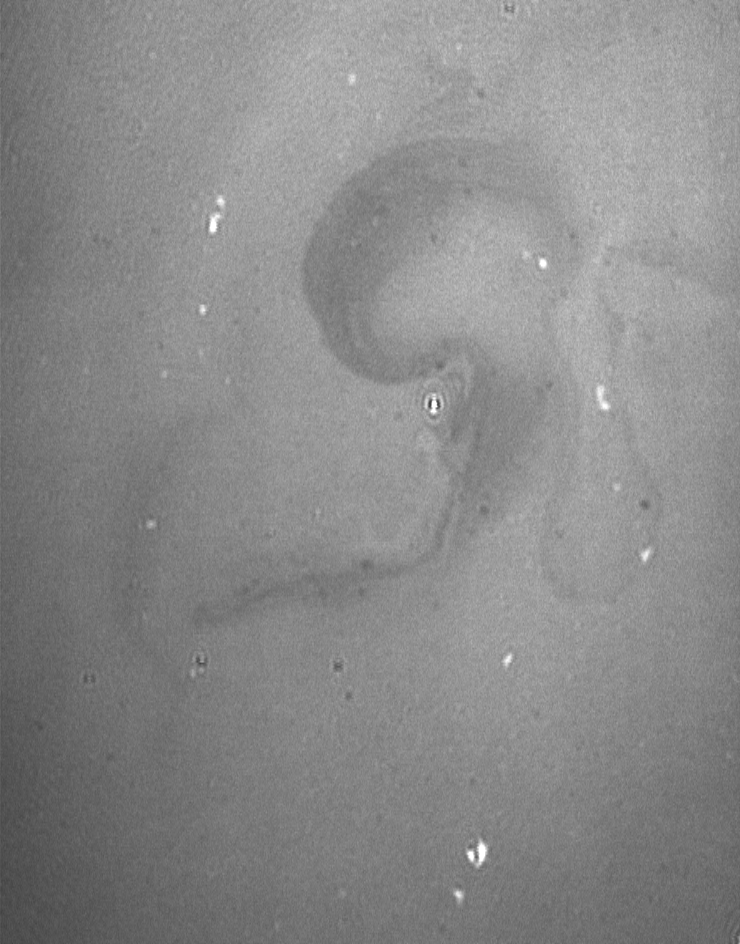

Supplement: Source Data Fig. 1 — SP traces, AFM images, BAM images. [file 41557_2023_1221_MOESM2_ESM.zip › Figure1/Figure1_b_c_d/All Data/FUS_PBS_59min.png]

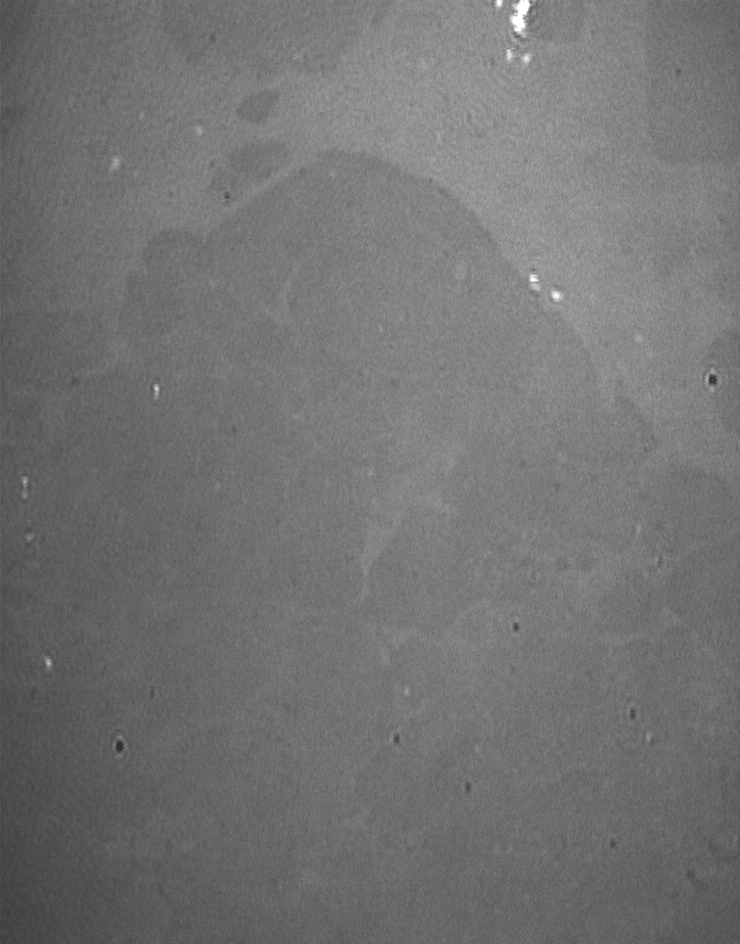

Supplement: Source Data Fig. 1 — SP traces, AFM images, BAM images. [file 41557_2023_1221_MOESM2_ESM.zip › Figure1/Figure1_b_c_d/All Data/FUS_PBS_74min.png]

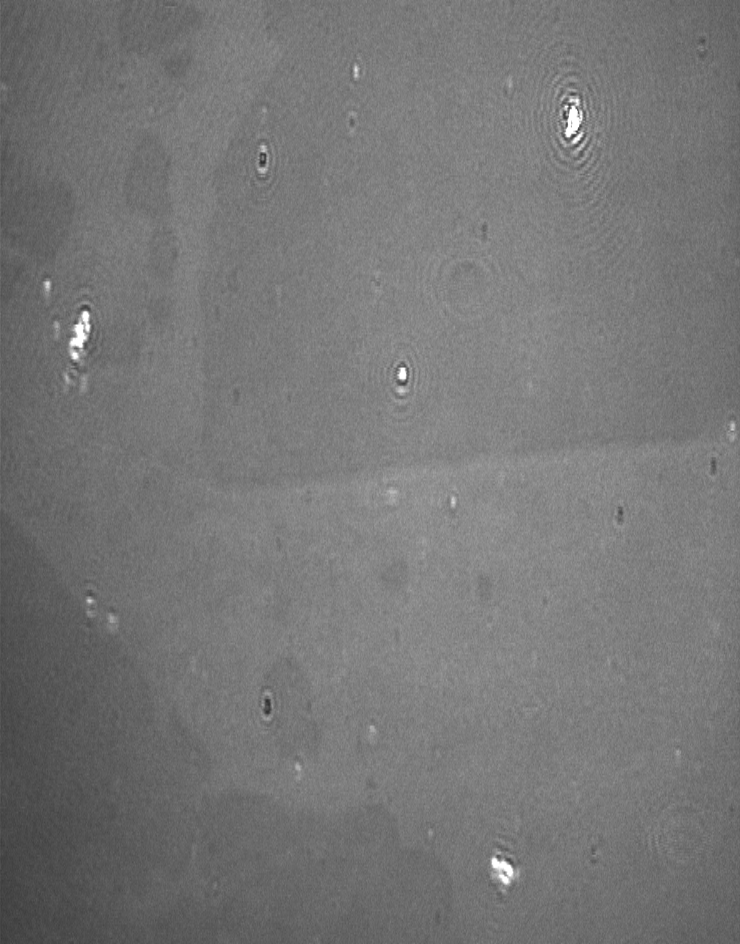

Supplement: Source Data Fig. 1 — SP traces, AFM images, BAM images. [file 41557_2023_1221_MOESM2_ESM.zip › Figure1/Figure1_b_c_d/All Data/FUS_PBS_75min.png]

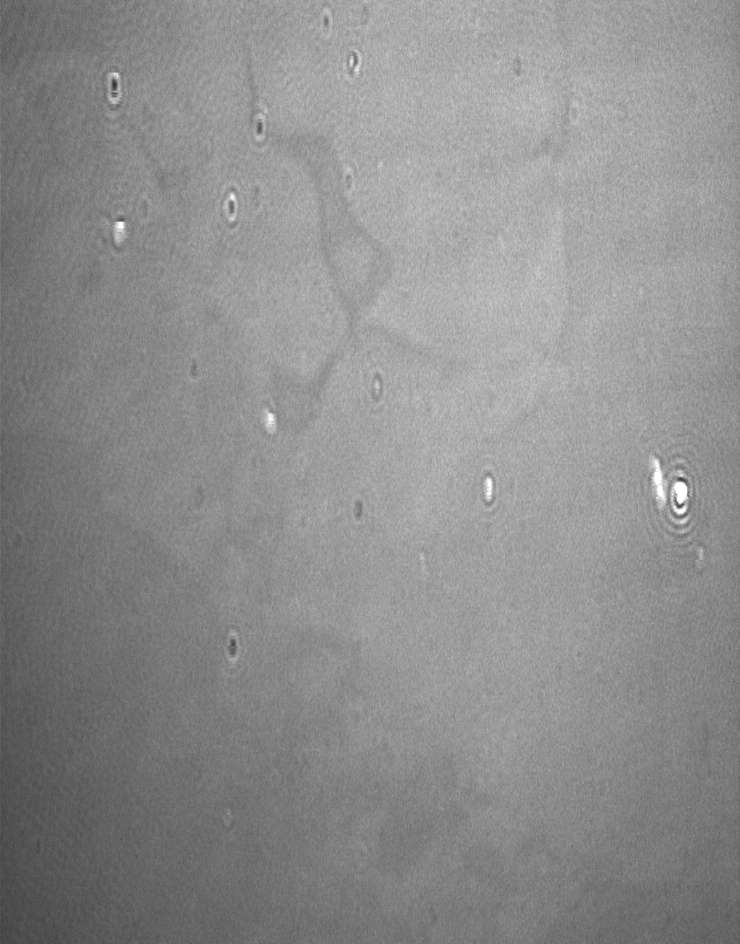

Supplement: Source Data Fig. 1 — SP traces, AFM images, BAM images. [file 41557_2023_1221_MOESM2_ESM.zip › Figure1/Figure1_b_c_d/All Data/FUS_PBS_77min.png]

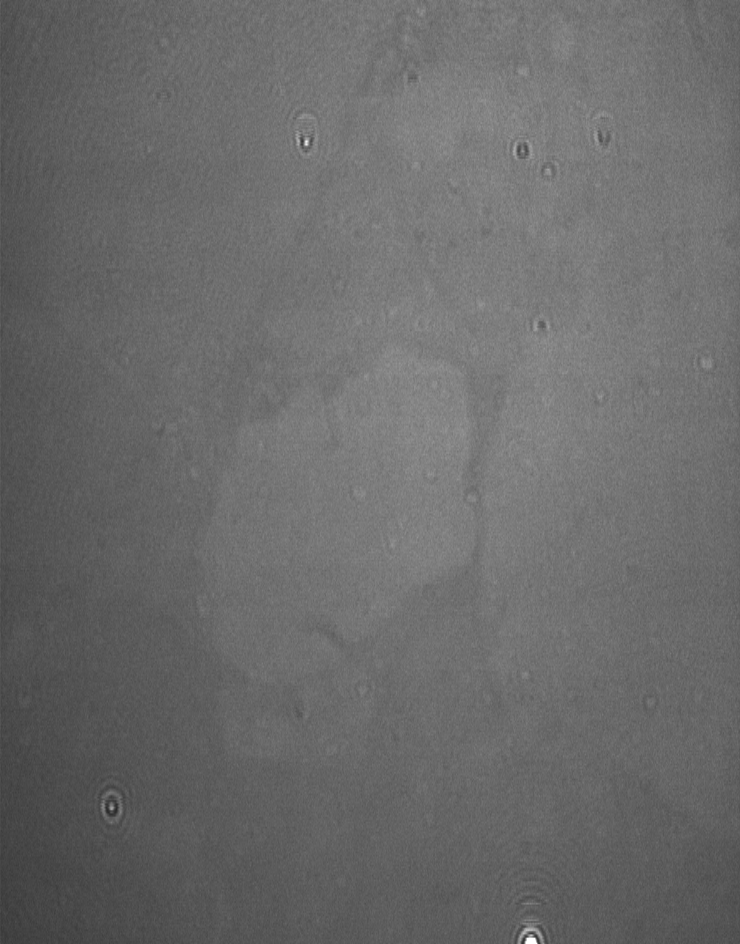

Supplement: Source Data Fig. 1 — SP traces, AFM images, BAM images. [file 41557_2023_1221_MOESM2_ESM.zip › Figure1/Figure1_b_c_d/All Data/FUS_PBS_80min.png]

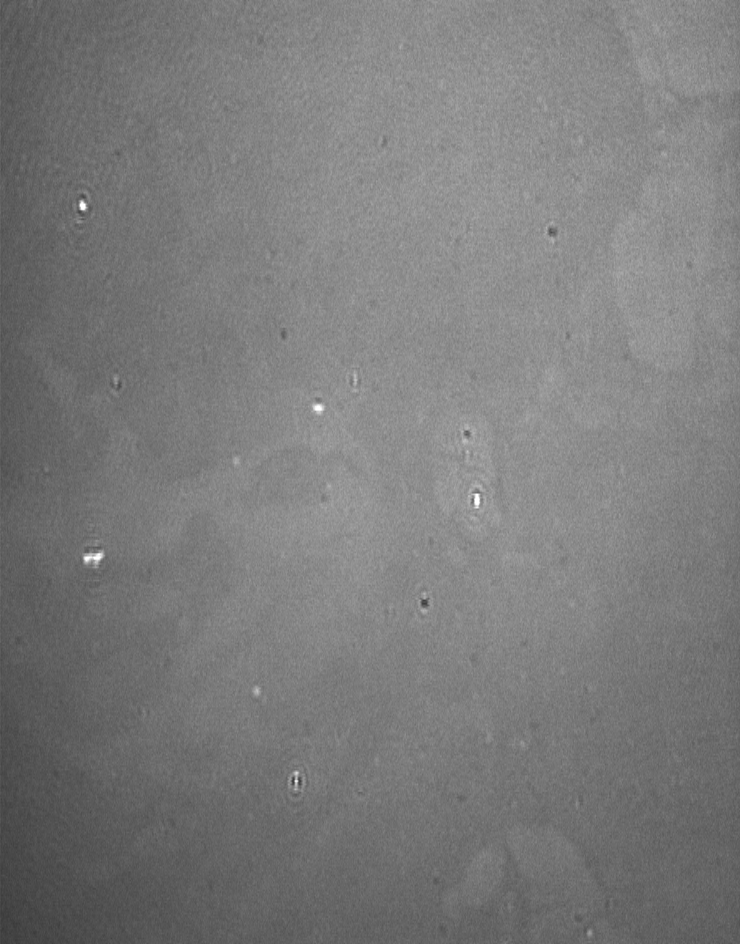

Supplement: Source Data Fig. 1 — SP traces, AFM images, BAM images. [file 41557_2023_1221_MOESM2_ESM.zip › Figure1/Figure1_b_c_d/All Data/FUS_PBS_81min.png]

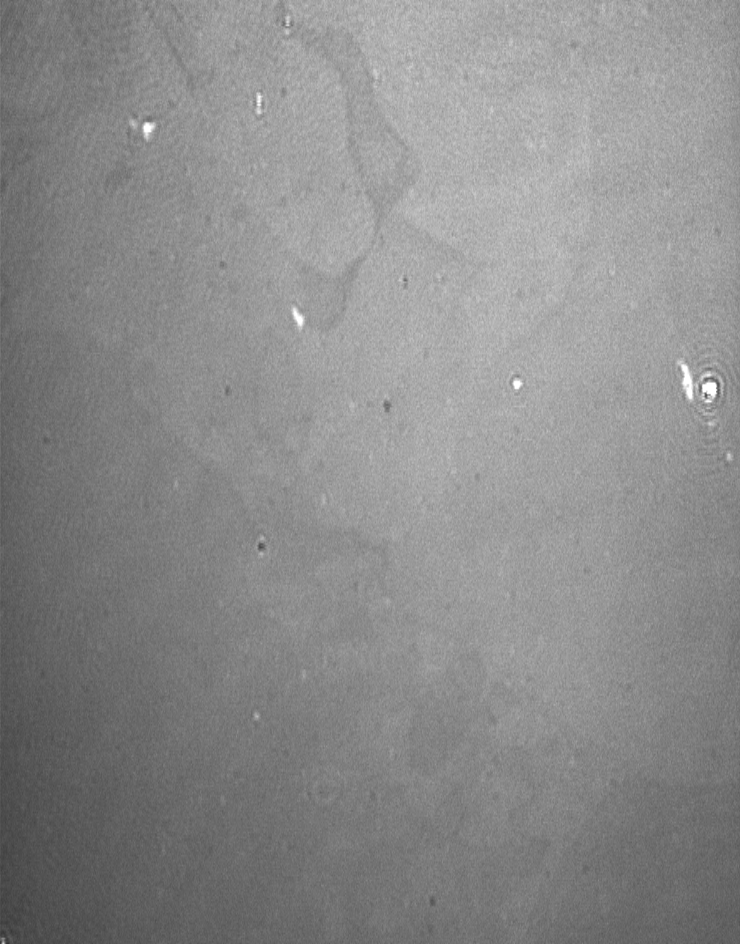

Supplement: Source Data Fig. 1 — SP traces, AFM images, BAM images. [file 41557_2023_1221_MOESM2_ESM.zip › Figure1/Figure1_b_c_d/All Data/FUS_PBS_83min.png]

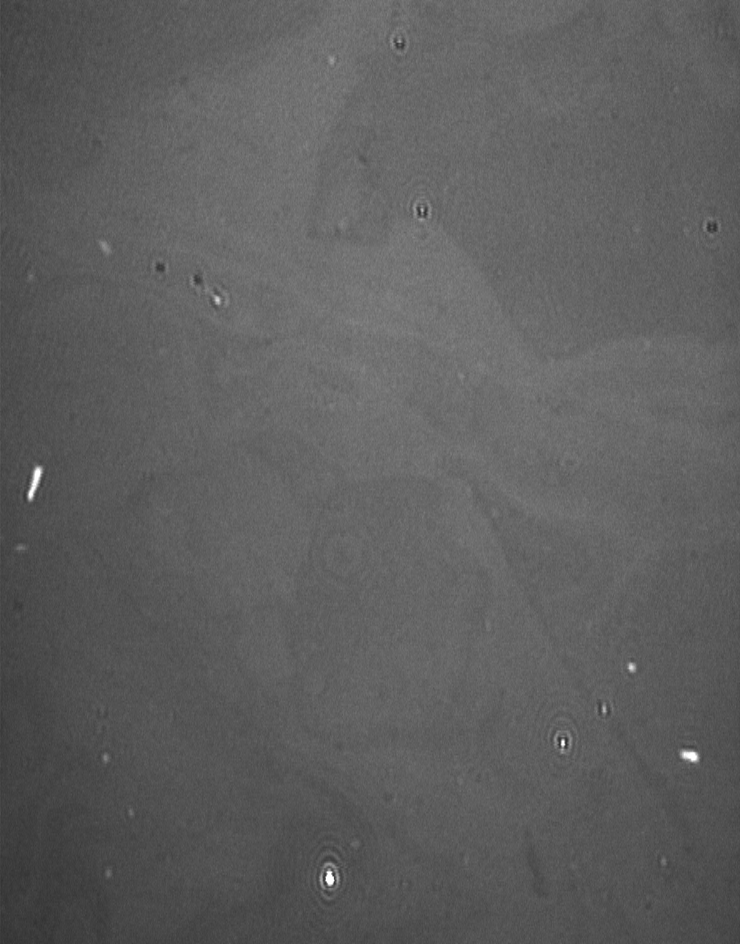

Supplement: Source Data Fig. 1 — SP traces, AFM images, BAM images. [file 41557_2023_1221_MOESM2_ESM.zip › Figure1/Figure1_b_c_d/All Data/FUS_PBS_85min.png]
